# Three LIF-dependent signatures and gene clusters with atypical expression profiles, identified by transcriptome studies in mouse ES cells and early derivatives

**DOI:** 10.1186/1471-2164-10-73

**Published:** 2009-02-09

**Authors:** Marina Trouillas, Claire Saucourt, Bertrand Guillotin, Xavier Gauthereau, Li Ding, Frank Buchholz, Michael Xavier Doss, Agapios Sachinidis, Jurgen Hescheler, Oliver Hummel, Norbert Huebner, Raivo Kolde, Jaak Vilo, Herbert Schulz, Hélène Bœuf

**Affiliations:** 1 Université Bordeaux, Bordeaux, France; 2 CNRS-UMR-5164-CIRID, Bordeaux, France; 3 CTSA, Département Recherches et Thérapies Cellulaires, Clamart, France; 4 MPI-CBG, Pfotenhauer Str. 108, 01307 Dresden, Germany; 5 Center of physiology and Pathophysiology, Institute of Neurophysiology and Center of Molecular Medecine, University of Cologne (CMMC), Robert Koch Strasse 39, 50931 Cologne, Germany; 6 Max-Delbrück Center for Molecular Medecine, MDC Berlin, Buch Robert Roessle, Strasse 10, D13125- Berlin, Germany; 7 Institute of Computer Science, University of Tartu, Liivi 2, 50409 Tartu, Estonia and Quretec Ltd, Ulikooli 6a, Tartu, Estonia

## Abstract

**Background:**

Mouse embryonic stem (ES) cells remain pluripotent *in vitro *when grown in the presence of the cytokine Leukaemia Inhibitory Factor (LIF). Identification of LIF targets and of genes regulating the transition between pluripotent and early differentiated cells is a critical step for understanding the control of ES cell pluripotency.

**Results:**

By gene profiling studies carried out with mRNAs from ES cells and their early derivatives treated or not with LIF, we have identified i) LIF-dependent genes, highly expressed in pluripotent cells, whose expression level decreases sharply upon LIF withdrawal [*Pluri *genes], ii) LIF induced genes [*Lifind *genes] whose expression is differentially regulated depending upon cell context and iii) genes specific to the reversible or irreversible committed states. In addition, by hierarchical gene clustering, we have identified, among eight independent gene clusters, two atypical groups of genes, whose expression level was highly modulated in committed cells only. Computer based analyses led to the characterization of different sub-types of *Pluri *and *Lifind *genes, and revealed their differential modulation by *Oct4 *or *Nanog *master genes. Individual knock down of a selection of *Pluri *and *Lifind *genes leads to weak changes in the expression of early differentiation markers, in cell growth conditions in which these master genes are still expressed.

**Conclusion:**

We have identified different sets of LIF-regulated genes depending upon the cell state (reversible or irreversible commitment), which allowed us to present a novel global view of LIF responses. We are also reporting on the identification of genes whose expression is strictly regulated during the commitment step. Furthermore, our studies identify sub-networks of genes with a restricted expression in pluripotent ES cells, whose down regulation occurs while the master knot (composed of OCT4, SOX2 and NANOG) is still expressed and which might be down-regulated together for driving cells towards differentiation.

## Background

Mouse embryonic stem cells (ES), which are derived from the inner cell mass of blastocysts, are valuable for studying pluripotency. Indeed, these cells recapitulate the complete mouse developmental program when injected into fertilized eggs and are widely used to introduce targeted mutations in mice [1,2]. In addition, they can be induced to differentiate *in vitro *to various cell types of the three germ layers and are therefore of great interest for study signalling pathways leading to specialized cell differentiation [3-5]. Knowledge of molecular determinants that regulate stem cell fate and understanding their functions are key challenges for future therapy based on stem cells in which their plasticity has to be properly controlled.

Two major pathways have been characterized so far, leading to the identification of "master genes" critical for the maintenance of mouse ES cell pluripotency: the LIF/STAT3 pathway, which synergizes with BMP2/4 and/or Wnt family members (as Wnt3a, Wnt5a and Wnt6, [6-8]), to maintain ES cell pluripotency alone [9-14], and the OCT4/SOX2 and NANOG pathways, the last one identified in cells in which the LIF pathway has been knocked-down [15-20]. The many known regulators of ES cell pluripotency, like *c-yes*, [21], *c-myc*, [22], *foxd3 *[20] and those characterized by gene profiling studies [23-26] might be classified in one or the other pathway. However, the exhaustive characterisation of transcriptional targets of LIF in ES cells and in their early differentiated derivatives has not been reported and the specificity of LIF targets versus those of OCT4 or NANOG has not yet been investigated [27-29].

LIF is a member of the Interleukin-6 (IL-6) family of cytokines, which displays pleiotropic functions, depending on both cell maturity and cell type [30-32]. Indeed, while LIF maintains ES cell pluripotency and is a critical cell survival factor in myocytes, embryonic germ cells, particular subtypes of neurons and osteoblasts, it induces differentiation of the M1 leukemic myeloid cell line, can switch the identity of neurons [33], induces cell cycle arrest in cancer cells [34] and has a key pro-apoptotic role during the post-gestation process of mammary gland involution [35-37].

In mouse ES cells, LIF induces signalling pathways including JAK1/STAT3/MYC/CD9/SOCS3/PI3K and ERK/RSK/CREB leading to activation of both anti- and pro-differentiative signals [23,38-44]. The transcriptional targets of LIF are the outcomes of these signalling cascades and their identification is a critical step for understanding the control of ES cell pluripotency. Also, LIF-dependent gene profiling performed in various cell states (pluripotent, reversible and irreversible commitment), should help to define different groups of LIF responsive genes, leading to a better understanding of pleiotropic effects of this cytokine.

The aim of this study was to characterize the LIF transcriptome of mouse ES cells as well as to identify novel regulators of the transition from pluripotent to irreversibly committed cells. By gene profiling studies, performed on ES cells cultured under various conditions, we have identified eight independent gene clusters including a novel gene category whose expression level varies transiently in reversibly committed cells only. Moreover, our strategy allowed us to identify different sets of LIF-regulated genes and to define those ones also regulated by OCT4 or NANOG. The effects of selected genes on ES cell differentiation were also investigated.

## Results

### 1) – LIF targets in mouse ES cells: experimental design

**Figure 1:**
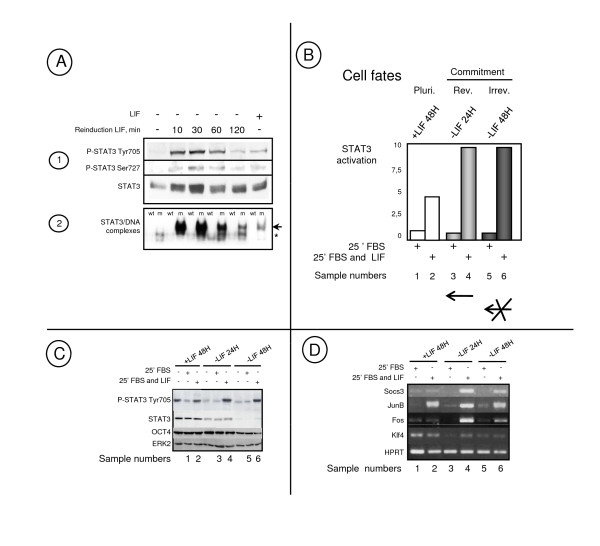
**The experimental strategy**. **A1) **Western blot analyses showing STAT3 and P-STAT3 and **A2) **band shift analyses of specific LIF-dependent complexes in ES cells depleted of LIF for 24 h and reinduced with LIF for 10 to 120 min. or in pluripotent cells (+ LIF) as indicated. LIF specific complexe containing STAT3 (arrow) and non specific complexe (asterisk) are indicated. **B) **Diagram summarising the cell growth conditions used for microarray experiments: Mouse ES cells, in the pluripotent state (grown with LIF for 48 h, Pluri.) or induced to differentiate by LIF withdrawal for 24 h [reversible commitment (Rev.)] or 48 h [irreversible commitment (Irrev.)], have been treated for 25 min. with 10% FBS or 10% FBS and 500 pM LIF (+), as indicated, before harvesting. Sample numbers correspond to the conditions compared in Tables 1 to 7 (see Additional file 1). **C) **Quality controls of LIF response: Protein RIPA cell extracts from ES cells grown with LIF for 48 h or without LIF for 24 or 48 h and not treated (-) or treated for 25 min with FBS or FBS and LIF as in **B)**. Western blot analysis were performed with antibodies reacting against all forms of STAT3 (STAT3), the activated STAT3 (P-STAT3 Tyr705) or with OCT4 and ERK2. **D) **Semi-quantitative RT-PCR performed with total RNAs from ES cells grown as indicated in **B)**, have been performed with primers corresponding to the indicated genes.

To identify LIF targets in pluripotent and committed-derived ES cells as well as key genes regulated during the transition from pluripotent to early differentiated cells, we carried out an experimental strategy based on these three facts i) A complete change of cell culture medium (including serum and LIF) is required every 48 h to induce a boost in STAT3 phosphorylation allowing cells to remain pluripotent, ii) 24 h after LIF withdrawal, STAT3 is inactivated by dephosphorylation and if incubated for longer in the absence of LIF, ES cells will differentiate. However, if LIF is added back 24 h after its withdrawal, differentiation is prevented and cells maintain their pluripotent state. Cells generated following 24 h of LIF starvation can therefore be categorised as in a state of reversible commitment, iii) 48 h following LIF withdrawal, while remaining LIF-sensitive, cells are irreversibly committed to differentiate. Indeed, even if LIF is added back, cells differentiate and a proportion of them die by apoptosis [24,38,45-47]. The LIF-dependent kinetic experiments of STAT3 activation showed a maximal stimulation at 30 min., which corresponds to the maximal level of phospho-tyr705 and phospho-ser727 STAT3 protein leading to the formation of active STAT3-DNA complexes (Figure 1A).

Gene profiling experiments were carried out with mRNA extracted from the CGR8 mouse ES cell line cultured under growth conditions as defined in Figure 1B. Cells in Pluripotent (Pluri), reversible (Rev.) or irreversible (Irrev.) commitment states were treated with serum or serum and LIF, 25 min. before harvesting. Protein lysates were also prepared in parallel with RNA samples. Analysis of LIF-dependent induction of STAT3, as detected by the extent of its phosphorylation, confirmed LIF responsiveness of pluripotent, reversibly, and irreversibly committed cells. In addition, we observed a progressive decrease in the levels of STAT3 protein after 24 h and 48 h of LIF starvation, which correlates with the loss of cell pluripotency. In contrast, no changes in OCT4 protein level was observed at these early times of cell differentiation. As expected, the amount of ERK2 proteins was unaltered under these various conditions and therefore, it was used as a protein loading control (Figure 1C). The quality of mRNA hybridized to microarrays was checked by RT-PCR experiments on a control (*Hprt*) and known LIF-induced genes as *Socs3, JunB *and *Fos *and the pluripotent *Klf4 *gene, [23,48], (Figure 1D).

### 2) Gene expression profiling in the absence or presence of LIF

**Figure 2:**
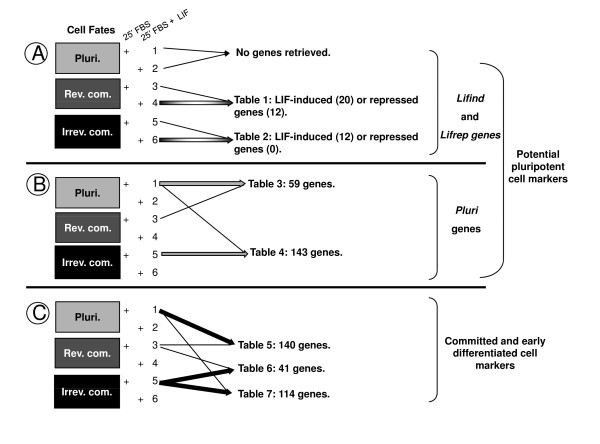
**Summary of pair-wise comparisons**. **A) **LIF-induced (*Lifind*) or repressed (*Lifrep*) genes, in pluripotent cells (samples 1 and 2) or after 24 h (samples 3 and 4) or 48 h (samples 5 and 6) of LIF withdrawal, **B) ***Pluri*. genes and **C) **Markers from reversibly and irreversibly committed cells, were retrieved by pair-wise comparisons after a student t-test analysis with the p-value and fold change cut off as indicated for each Table, see Additional file 1. Each thick line indicated the condition for which the number of genes has been filtered, after t-test analysis, versus the other condition. Pluri: Pluripotent; Rev.com.: Reversible commitment; Irrev. com.: Irreversible commitment.

Five independent total RNA preparations from each condition, (Figure 1B), were processed and hybridized on the Mouse Genome 430 2.0 Array (Affymetrix) which includes 45101 probe sets. After RMA normalisation [49] and outlier removal, pairwise comparisons have been performed using the paired Student t-test (summarized in Figure 2). This allows the classification of genes into 7 tables (Tables 1 to 7, see Additional file 1). In addition, multiple testing correction has been performed using the Benjamini and Hochberg procedure [50,51], as described in the Methods section.

Tables 1 and 2 show the sets of the LIF-regulated genes detected in ES cells reversibly and irreversibly committed to differentiate following acute treatment with LIF. Genes whose expression was induced by LIF, named hereafter *Lifind *genes, belong to various gene families including the Early growth response genes (*Egr1,2*), members of Immediate early response (*Ier2 *and *3*), and of Kruppel-related gene (*Klf4 and 5*) families, *Ras-dex1*, *Ypel2 *and the PI3K regulator *Dapp1*. In addition, an EST of unknown function, *1459961_at*, encoded by a gene localized at proximity of the *Stat3 *locus (5' to the promoter) has also been retrieved (named *Stat3Loc*, see Additional file 2). A subgroup of genes induced after 24 h of LIF withdrawal was also induced after a 48 h period of LIF starvation (compare Tables 1 and 2). In addition, few LIF-repressed genes (*Lifrep *genes), with the extent of repression less than 2- fold, were retrieved 24 h after LIF withdrawal (*Ddh2*, *Mtss1*, *Chd8 *and ESTs), and no repressed genes were identified after 48 h of LIF starvation. While our stringent criteria of gene selection by the t-test analyses do not lead to the identification of LIF-modulated genes in pluripotent cells (+ LIF conditions), we detected a LIF-dependent increased expression of some genes, including *Ypel2*, *Plscr1*, *Dapp1*, *JunB*, *Stat3Loc *and *Klf5*, in pluripotent cells, by semi-quantitative RT-PCR analysis (see below, Figure 4). Altogether, these results showed that the LIF response was different in pluripotent, reversibly and irreversibly committed cell populations, suggesting that LIF outcomes do not rely on the sole activation of STAT3, similarily induced in these three conditions.

Tables 3 and 4 depict pair-wise comparisons of genes whose expression was significantly diminished after 24 h (Table 3) or 48 h of LIF starvation (Table 4) in comparison to pluripotent cells (+LIF conditions). In this analysis, *Lifind *genes, as defined in Table 1, were also retrieved (as *Socs3*, *Junb *and *Klf4*) indicating that some cell fate-regulated genes could also be reinducible by LIF, a property not shared by the majority of genes retrieved in these Tables. This analysis allowed to identify the core "*Pluri*" genes including known pluripotent markers such as *Stat3*, *Nanog*, *Klf4*, *Yes*, *Zfp42/rex1*, *Bcl3*, *Spp1*, *Cd9, Esrrb, Tbx3, Tcl1 *[25,47] and members of gene families not yet described to be regulated in the ES cell model. We identified genes encoding for proteins involved in cell adhesion (as *Ceacam1 *and *2*), Wnt (*Fzd5*, *Mitf*, *Aes*), Ras (*Mras*, *Ulk1*), Notch (*Notch4*), Interleukin1 (*Irak3*) and Tgfb (*Bmp4*, *Inhbb*) signalling pathways, in cytoskeleton organization (*Mapt*/*Tau*, *Myosin IF*, *Pdgfa*), and in lipid (*Abca1*, *Cpt1a*) and superoxide anion metabolism (*Sod2*). Many transcription factors (*Tcfcp2like1*, *Trps1*, *Sox21*, *Arid5b*, *Nr0b1*, *Zfp57*, *Musculin*) and chromatin regulators (*Myst4*) were also identified.

**Figure 3:**
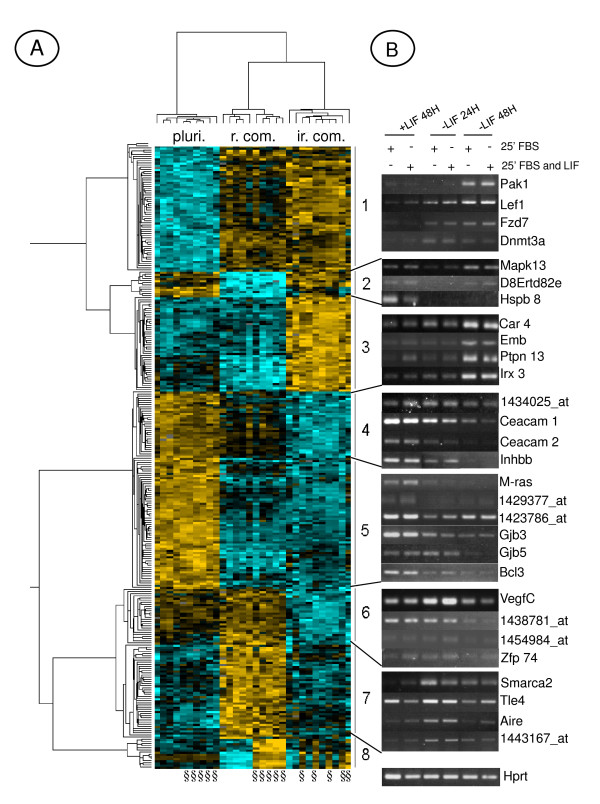
**Identification of eight independent gene clusters. A)** Each probe set is represented by a single row of colored boxes and single column represents each microarray. Cells with increasing up regulation colored with yellow of increasing intensity and increasing downregulation is colored with blues of increasing intensity. The similarity trees on the top (pluri.: Pluripotent; r. com.: reversible commitment; ir. com.: irreversible commitment) and on the left side, represent the similarity matrix of probe sets or microarrays. §: Samples treated 25 min. with FBS and LIF. **B)** Semi-quantitative RT-PCR of a selection of genes from clusters 1 to 7. Representative results, from 4 independent experiments, are shown.

Tables 5, 6 and 7 gather genes whose expression increases upon LIF withdrawal. Twenty genes, whose expression level was up regulated in irreversibly committed cells versus pluripotent cells, were also expressed in reversibly committed cells (genes quoted as "YES" in Table 5, like *Lef1, Wt1*, *Dnmt3a *and *b*, *Oct6 *and *Otx2*). This findings suggest that irreversible commitment maybe linked to the sustained expression of a set of genes expressed at earlier time following LIF withdrawal. In table 5, we noted that the majority of genes (120 out of 140) has a particular expression profile characterized by low level of expression in pluripotent cells, high level of expression in reversibly committed cells and low level of expression again in irreversibly committed cells ("low-high-low" profile, quoted as "NO", in Table 5). These genes, including *Bhc80*, *Bicd1*, *Six4*, *Bmi1, Aire, Jmjd1c, Jmjd2c *and *Tle4*, define a new category of genes, whose expression is transiently induced in reversibly committed cells (see also below, in Figure 3). Table 7 includes genes induced at 48 h versus 24 h of LIF starvation. Some of these genes are specific of the irreversibly committed cells.

### 3) Gene clustering of all conditions

We performed an ANOVA (F-test) on data obtained under the different cell growth conditions. The expression profiles of highly significant differentially expressed genes [pvalue < 10^-6 ^with a FDR (False Discovery Rate) of 1.48. 10^-4^; n = 292] have been categorized using hierarchical clustering. This allowed to group genes with similar expression profiles, an indication of potential co-regulation and common functions [52]. Tree view representation of the clusters led to the identification of eight independent groups of genes (Figure 3). The upper dendrogram shows the expected clustering of the different cell growth conditions with the pluripotent state in one branch (pluri.), and the reversibly (r. com.) and irreversibly (ir. com.) committed conditions in the other branch. The complete list of genes of the cluster analysis is available (see Additional file 3).

Cluster 1 (similarity score = 0.7547) includes genes whose expression, induced at 24 h of LIF starvation, is sustained at 48 h (*Foxp1*, *Enpp3*, *Otx2*, *Pak1*, *Wt1*, *Gja1*, *Lef1 *and *Fzd7*). Cluster 3 (similarity score = 0.8354) defined the differentiated markers specific of the irreversibly committed cells, like *Car4*, *Embigin*, *Fgf5*, *Ptpn13*, *Irx3*, *Fgfr11*, *Rbp7*, some of them corresponding to known cell lineage markers such as *Fgf5*.

Clusters 2 (similarity score = 0.7938) and 7 (similarity score = 0.7109), are the mirror image from each other and define new atypical gene behaviors. Modulation in the expression level of these genes was only detected in the reversibly committed cell population. Indeed, the expression profile of these genes is: "high-low-high" (Cluster 2) or "low-high-low" (Cluster 7) in "pluri – r.com – ir.com" cell growth conditions. Among genes from Cluster 2 we identified *Vimentin*, *Hspb8 *and *Jund2*. Genes from Cluster 7 include transcription factors like *Mlr2*, *Aire, Tle4 *and chromatin regulators as *Bhc80, Smarca2*. Genes from these clusters might be necessary to regulate the transition from pluripotent to irreversibly committed cells and their identification would not have been possible without the clustering of all data obtained in our experimental conditions.

Clusters 4 (similarity score = 0.8109), 5 (similarity score = 0.8170) and 6 (similarity score = 0.7163) define genes highly expressed in pluripotent cells and whose expression level, was i) abruptly decreased 24 h following LIF starvation (in Cluster 5: *Pim3*, *Fzd5*, *Sod2*, *Esrrb*, *Tcfcp2l1*, *Mras*, *Gjb3 *and *5*, *Bcl3*, *Stat3*, *Smarcd3*, *Yes *and *Cd9*), ii) progressively decreased, (in Cluster 4: *Stmn1 *and *2*, *Scarb2*, *Timp1*, *Pcolce, Tcl1 and Trps1*) or iii) decreased only 48 h after LIF starvation (in Cluster 6: *Lgals3*, *Cordon bleu*, *Vegfc*, *Zfp74*, *Hist1h1c*). Genes from cluster 5 are named "*Pluri*" genes thereafter.

Cluster 8 (similarity score = 0.7488) includes genes whose expression was induced by LIF in both reversibly or irreversibly committed cells. Only one gene (probe set 1446583_at) was found repressed by LIF in this analysis.

**Figure 4:**
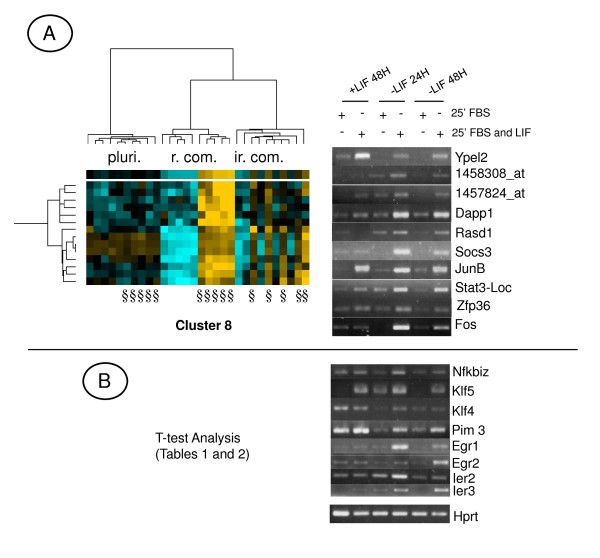
**Highlights on Cluster 8. A)** Tree view representation of Lifind genes, regulated similarly by LIF in r. com. (reversible commitment) and ir. com. (irreversible commitment) conditions and validation of a selection of genes by semi-quantitative RT-PCR, **B)** Validation of a selection of *Lifind* genes retrieved by the t-test analysis. §: Samples treated 25 min. with FBS and LIF. Representative results, from 4 independent experiments, are shown.

The regulated expression of a selection of genes from each cluster and Tables 1 and 2 (see Additional file 1) was validated by semi-quantitative RT-PCR (Figures 3B, 4A and 4B).

### 4) Cell fate-independent and cell-specific *Lifind *genes

**Figure 5:**
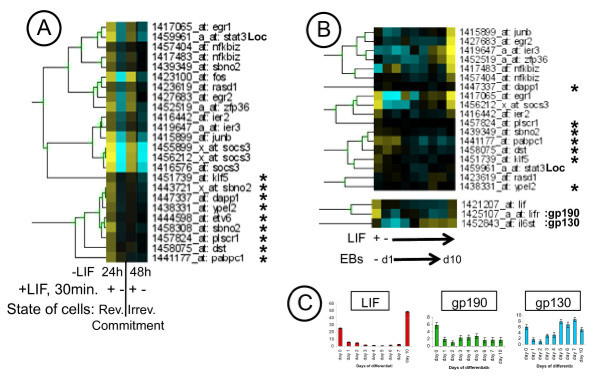
***In silico* analyses of *Lifind* genes in various microarray experiments.** Tree view representation of Heatmapper analyses of *Lifind* genes retrieved from Table 1 and analysed with Affymetrix data from reversibly (rev.) and irreversibly (irrev.) committed cell populations **A)**, and in a kinetic of LIF withdrawal [day 1 (d1) to day 10 (d10) in EBs], **B)**. The validation of the expression of LIF cytokine and of its composite receptor (gp130 and gp190), performed by RT-qPCR on three independent experiments, is shown as histograms with standard deviations in **C)**. The asterisks point to genes not reinduced by LIF in differentiated cells (d10 EBs).

To determine whether the *Lifind *genes from Table 1 (see Additional file 1) are modulated by LIF in other cellular context, we analysed their expression profile with the heatmapper tool, under our set of conditions (Figure 5A), and in an embryoid body (EB) kinetic analysis following LIF withdrawal, from day 1 to day 10 (Figure 5B). Indeed, we had previously shown that some LIF targets (like *Socs3*, *Fos *and *JunB*) are expressed, several days after LIF withdrawal, in differentiated cells [46]. In this study, we extended this analysis and showed that almost all the genes induced by LIF in reversibly and irreversibly committed cells, are re-expressed, concomitantly with the re-expression of LIF and of its receptors (gp130 and gp190) at day 10 of EB differentiation (Figures 5B and 5C). In contrast, genes marked with an asterisk in Figure 5A are not re-expressed (see Figure 5B). Therefore, we have characterized two types of *Lifind *genes: those induced by LIF at 24, 48 h and 10 days after LIF withdrawal and which correspond to cell fate-independant LIF targets [named hereafter "*Pleio-Lifind" *genes and including *Socs3, Fos *and *JunB*] and genes whose induction by LIF is cell-restricted [named hereafter "*Spe-Lifind"*genes] and which were not known, so far, as LIF targets.

### 5) Regulation of "*Pluri*" genes in embryoid bodies kinetic following LIF withdrawal

**Figure 6:**
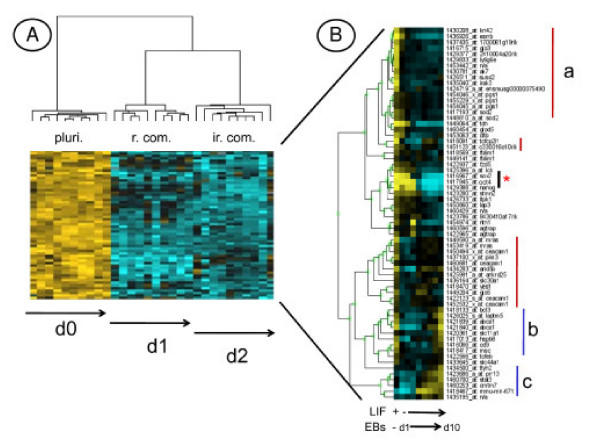
***In silico* analysis of *Pluri* genes.** Heatmapper analysis of genes from Cluster 5 (as defined in Figure 3 and represented here in panel **A)** has been performed with Affymetrix data taken from the kinetic of LIF withdrawal (d1 to d10 of EBs differentiation), **B)**. Relevant clusters are highlighted by red (a) or blue lines (b and c). The asterisks point to three master genes, *sox2*, *oct4* and *nanog* manually included in this analysis.

Genes from Cluster 5 (represented in Figure 6A), were also analysed with the heatmapper tool in the time course of EB differentiation (Figure 6B). Master genes, *Oct4*, *Sox2 *and *Nanog*, whose expression is stable up to day 2 (*Sox2*) or day 3 (*Oct4 *and *Nanog*) and which are therefore not present in cluster 5, were manually added for this analysis. Three groups of genes were identified: i) those strictly expressed in pluripotent cells (red line, Cluster a), including pluripotent cell marker like *Esrrb*; ii) those re-expressed at day 10 of the EB diffentiation kinetic (blue line, Cluster b), including the LIF-dependent *Cd9 *gene [39], and iii) genes whose expression was higher in differentiated than in pluripotent cells (blue line, Cluster c), including *Stat3*. This analysis helps to identify relevant new genes potentially involved in the maintenance of ES cell pluripotency, since known pluripotent markers are present in each of these clusters.

### 6) Analyses of *Lifind *and *Pluri *gene expression in *Oct4 *and *Nanog *knock-down experiments

**Figure 7:**
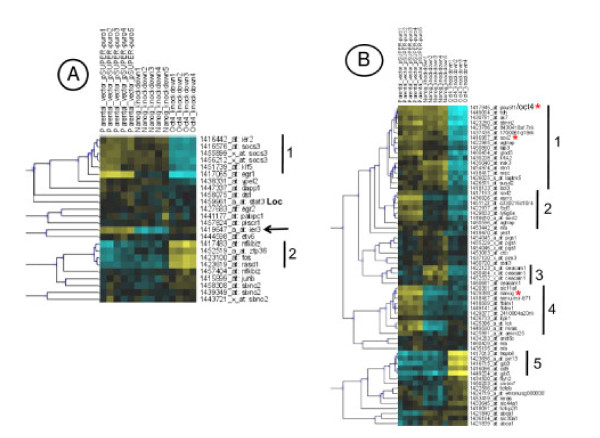
***In silico* analysis of *Lifind* and *Pluri* genes: Regulation by OCT4 or NANOG.** Heatmapper analyses performed respectively with *Lifind***A)** and *Pluri***B)** genes whose level of expression was analysed in microarray experiments taken from Loh et al, [19]. Relevant clusters are marked with black lines and numbered. *Color code* is like in Figure 3. yellow: expression above the median; black: median expression; blue: expression below the median.

The overall expression level of *Oct4 *and *Nanog *was unaltered up to three days upon LIF withdrawal in the kinetic of embryoid bodies (Figure 6B). However, the recent finding that ES cells are heterogeneous for the expression of many markers, like NANOG but no OCT4, prompted us to determine whether the expression level of genes identified in this study could be directly modulated by these master genes. This should help to find a specificity of these core "pluripotent master genes" [53,54]. We have analysed expression profiles of *Lifind *and *Pluri *genes after knock-down of *Nanog *and *Oct4 *by RNAi. Availability of Affymetrix data, from Loh et al, [19], performed with mouse ES cells infected with lentivirus expressing sh*Nanog *or sh*Oct4 *silencing RNAs, allowed such an analysis. Heatmapper analyses were performed with *Lifind *(Figure 7A) or *Pluri *genes (Figure 7B). The expression of *Lifind *genes are either repressed like *Ier2*, *Socs3, klf5 or Egr1 *(Figure 7A, Cluster 1) or induced like *Zfp36*, *Fos *and *Rasd1 *(Figure 7A, Cluster 2) by *Oct4 *knock down. Remarkably, *Ier3 *is the only *Lifind *gene whose expression is repressed by *Nanog *silencing. In addition, this analysis emphasizes the particularity of some *Spe-Lifind *genes (like *Pabpc1, Plscr1, Dapp1 *and *Dyst*) and of *JunB*, *Sbno2 *and *Egr2 *whose expression is altered neither by *Oct4 *nor by *Nanog *silencing.

A similar analysis has also been conducted with the *Pluri *genes (Figure 7B), highlighting their differential regulation by *Oct4 *or *Nanog*. Indeed, we identified 5 groups of genes whose expression is down (like *Sox2*, *Irak3 *and *Susd2 *in Figure 7B, Cluster 1) or up (like *Cd9, Gjb3 *and *Gjb5 *in Figure 7B, Cluster 5) regulated following *Oct4 *but not *Nanog *silencing or down regulated by independent silencing of both genes (like *Esrrb, Ly6 *and *Sod2 *in Figure 7B, Cluster 2 and part of Cluster 4 including *Slc11a1*, *Nanog *and *Fblim1*). The expression of only one gene (*Ceacam1*) was increased by *Nanog *silencing (Figure 7B, Cluster 3). These analyses define novel gene categories, not similarly regulated by master genes, which might be part of novel sub-networks involved in the regulation of ES cell pluripotency.

### 7) Effects of knock-down of a selection of *Lifind *and *Pluri *genes in pluripotent cells

*In silico *analyses allowed us to select genes among the *Lifind *and *Pluri *genes for further functional studies. We focused our efforts on a selection of *Spe*-*Lifind *genes (*Dapp1*, *Plscr1*, *Dyst*, *Pabpc1*) and also included *Ier3 *owing to its particular expression profile in *Oct4 *and *Nanog *silencing experiments. Concerning the *Pluri *genes, we performed the experiments with genes specifically expressed in pluripotent cells such as *Irak3, Susd2 *and *Ly6 *and whose expression was affected by only one master gene (*Oct4*), except *Ly6*, which behaves as *Esrrb*, a recently identified pluripotent marker [25,55,56]. We have also included *Tcfcp2l1*, a transcription factor strictly expressed in pluripotent cells (Figure 6B, Cluster a).

We have tested the function of these genes using an RNA interference strategy. Endoribonuclease prepared (e) siRNAs [57] were generated, targeting the different genes. ES cells were transfected twice with each esiRNA and stained with Alkaline phosphatase (ALP) five days after the first transfection (see Additional file 4). Knock-down of *Stat3 *and *Oct4 *led to morphological cell differentiation (with loss of ALP staining), and proliferative defects similar to that observed when LIF was withdrawn for 4 to 5 days, consistent with their known function in maintaining pluripotency (positive controls). Knock-down of the various genes tested did not induce significant morphological changes, as shown for *Ier3 *as well as for the other genes tested (see Additional file 4 and results not shown). However, alteration in gene expression level (as shown in Figure 3) occurred much earlier than morphological changes, which are observed at three days upon LIF withdrawal (see Additional file 4A) and as morphological changes, which are observed at three days upon LIF withdrawal, as shown in Figure 3A and as reported in other ES cell lines, [47,58]. This prompted us to analyse the effects of gene silencing on the expression of early differentiation markers. Expression level of *Lef1, Pak1, Car4 *and *Dnmt3a*, all induced at 24 or 48 h of LIF withdrawal (see Figure 3), was analysed after 5 days of transfection in ES cells grown with LIF. The expression of *Nestin*, *Gata4*, *Gata6 *and *Sox17*, induced at later times of LIF withdrawal, was also studied. The efficiency of the silencing of each esiRNA was determined by RT-qPCR and shown to be in the range of 50 to 80% (see Additional file 5 and not shown). The expression of almost all the differentiation markers tested was modulated by the silencing of *Stat3 *or *Oct4*. However, individual silencing of the selection of *Lifind *or *Pluri *genes led to no effects or modest up regulation of some of the tested differentiation markers (see Additional file 5).

## Discussion

### From pluripotent to irreversibly committed cells: The LIF impact

**Figure 8:**
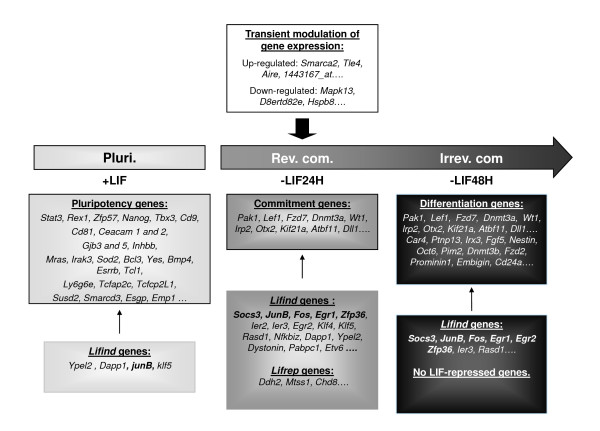
**Highlights of microarray analyses performed in pluripotent, reversibly and irreversibly ES-derived committed cells, treated or not with LIF.** A selection of genes regulated in each cell state is presented. Bolded genes are the *pleio-Lifind* genes, identified in the ES cell system or in other LIF sensitive cell types.

By gene profiling studies performed with pluripotent murine ES cells and with reversibly or irreversibly ES-derived committed cells, treated or not with LIF, we end up with a new global view of the genetic program leading from pluripotent to differentiated cells, as summarized in Figure 8: When LIF is withdrawn for 24 h, cells enter the commitment phase and the expression of 59 genes declines (like *Bcl3*, *Klf4*, *Stat3*, *Yes*, *Esrrb*, *Nanog*, *M-ras, Fzd5*), while the expression of 140 genes increases (like *Lef1*, *Otx2*, *Wt1*, *Dnmt3a*, *b and *Fzd7). When LIF is added back at this stage, twenty genes are induced (like *Socs3*, *Klf4 and 5*, *JunB, Egr1, 2, 3, Ier 1, 2, 3; Zfp36*) and twelve are repressed. This type of treatment allows cells to maintain their pluripotent state, as illustrated by their ability to colonize blastocysts and produce chimera almost as efficiently as cells kept under the continuous presence of LIF [24,38,45,47,59]. When LIF is withdrawn for 48 h, the set of genes repressed, in comparison with the pluripotent cells, is larger than at 24 h of LIF starvation. Some of these genes (16 genes, like *Oct6*, *Lef1*, *Otx2*, *Wt1*, *Dnmt3a*) are already expressed by reversibly committed cells suggesting that their sustained expression might be critical for the establishment of the differentiated state. Among them, *Otx2 *was recently shown to be a critical regulator of early cell differentiation [25]. LIF treatment of cells starved of LIF for 48 h does not reset the stemness program as shown by their inefficiency to form teratomas or colonize blastocysts [45,59]. Our statistical study reveals differences in the set of LIF targets induced after a 24 h or a 48 h period of LIF starvation. Indeed, only a portion of the genes identified in reversibly committed cells was induced by LIF in irreversibly committed cell population. In addition, the extent of LIF induction was less than half that observed in reversibly committed cells, despite the fact that STAT3 phosphorylation and activity remained very high in both situations. This suggests that activated STAT3 could regulate a cell-state-dependent pathway which locks cells in a differentiated state.

### Three LIF-dependent signatures

**Figure 9:**
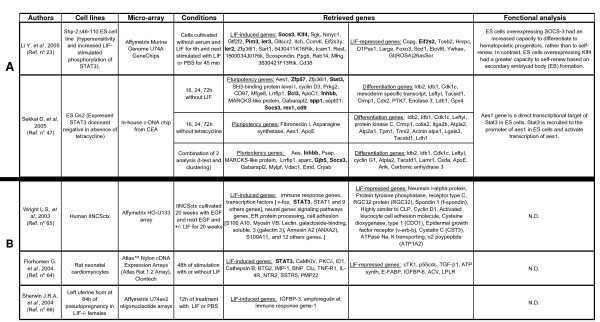
**A) Compilation of a selection of microarray data and comparison with our results.** Microarray experiments have been compared with our analysis. Genes in common with those identified in our analysis are bolded. **B) Comparison of *Lifind* genes in ES cells and other cell types and organ**. This table is a compilation of results obtained in various LIF-responsive cell types (cardiomyocytes and neurons) and organ (uterus).

Our analysis has allowed also to distinguish between S*pe-Lifind *(genes induced by LIF in a restricted cellular context), *Pleio-Lifind *(genes induced by LIF in various cellular context and potentially involved in pleiotropic effects of LIF) and *Pluri *genes. This last category corresponds to LIF-dependent genes, not reinducible by LIF in the first 48 h of differentiation (summary in Figure 8). Some of the *Pleio-Lifind *genes are known for their impact on stemness, [like *Klf4 *or *Socs3 *[23,41,48]], in trophoblast differentiation (like *Socs3*, [60]) and for their activation in the M1 myeloid cell line, in which LIF is pro-differentiative (like *JunB *and *Egr1 *[61-63]). However, none of the *Spe-Lifind *genes have previously been identified as LIF targets. Many of these genes are not well characterized, and their potential responsiveness to LIF could help unravelling their function. In addition, the comparison of LIF regulated genes identified in our study with those reported in microarray analyses, performed on other cell types or tissue [cardiomyocytes [64], neurons [65] and uterus [66]], shows no overlap except *stat3 *(summary in Figure 9). However, both the different timing of LIF induction (25 min. in our study versus several hours in these other studies) and cell context, [48,67,68], might account for this absence of overlap. Our screen reveals also a partial overlap between LIF targets identified in this study, and STAT3 targets, previously reported [47,69] suggesting that LIF and STAT3 pathways might have various outcomes which are not yet fully identified, and that LIF-dependent response might not be under the sole control of STAT3.

Our screen revealed that there are few LIF-repressed genes. This emphasizes the point that LIF effects are regulated through the synthesis of repressors (e.g., *Socs3*). Mereover, new LIF-induced negative regulators might be critical for maintenance of cell pluripotency. Such a candidate could be *Zfp36*, which encodes for a protein involved in mRNA stabilization through the ARE (Au Rich Element) sequence [70,71]. We found that a proper level of this protein was necessary to ensure survival of pluripotent cells since its overexpression led to cell death (our unpublished results). Along with the recent finding that ZFP36 regulates directly the stability of *Ier3 *mRNA [72], a new *Lifind *gene identified in that study, it is tempting to propose that differential stabilisation of mRNAs could have an impact on the survival of pluripotent ES cells.

### Lifind genes regulation by *OCT4 *or *NANOG*

**Figure 10:**
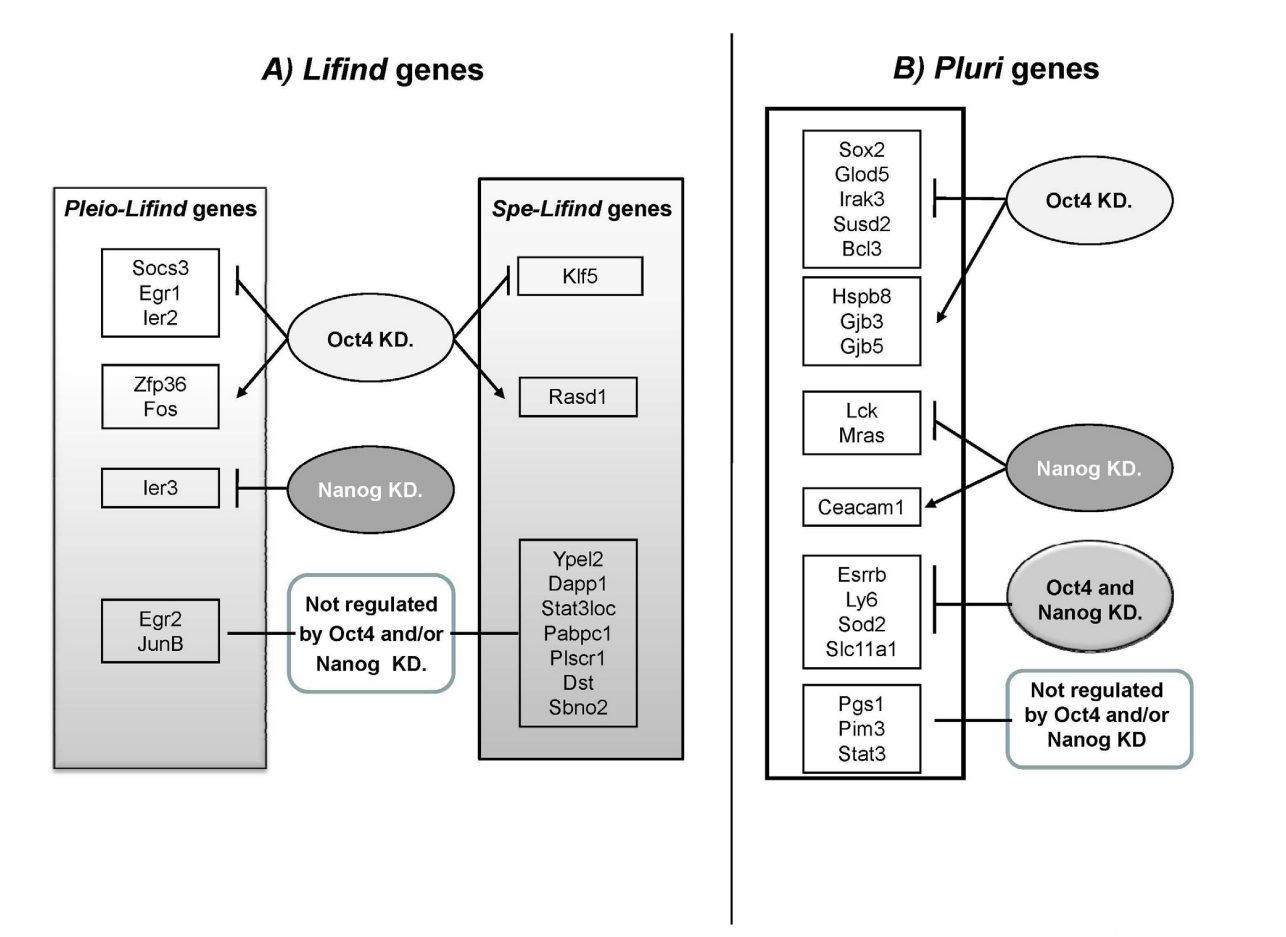
***Lifind* and *Pluri* gene expression in ES cells in which the expression of *Oct4* or *Nanog* has been knocked-down.** Summary of *Pleio-* and *Spe-Lifind* gene regulation **A)**, and of *Pluri* gene regulation **B)**, by *Oct4* and/or *Nanog*, based on the heatmapper analysis.

We have classified *Lifind *genes into different sub-groups depending on their differential regulation by *Oct4 *or *Nanog *(summary in Figure 10). Expression of almost none of the *Spe-Lifind *genes is modulated by the silencing of these two master genes, emphasizing their belonging to a LIF specific pathway [18,20,73]. However, the individual knock-down of four of these genes (*Dapp1, Plscr1, Dst *and *Pabpc1*) does not lead to morphological changes nor to a significant alteration in the expression of a selection of early differentiation markers. Disruption of all four genes together might be required before any effect becomes apparent. Alternatively, these genes could be involved in the reversibility of committed cells grown without LIF for 24 h, [38,74], a potentiality not tested in the present study.

### Novel characteristics of *Pluri *genes and differential regulation by *OCT4 *or *NANOG*

The *Pluri *genes retrieved in this analysis include genes previously shown to maintain cell pluripotency like *Yes*, *Cd9*, *Esrrb*, *Tcl1 *and *Tbx3 *[21,25,33,39]) and many novel genes (summary in Figure 8). We show that *Pluri *genes could be endowed with new features, which might be relevant to explain cell fates. Indeed, while expression of known master genes (*Oct4, Sox2 *and *Nanog*) is maintained for 2 or 3 days following LIF withdrawal, expression of almost all the genes identified in our analysis (Figure 3A, Cluster 5) is decreased on the first day of LIF withdrawal like *Esrrb *or *Pim3*, recently identified as new pluripotent genes [25,75]. The direct targets of *Esrrb *and *Tcfcp2l1 *(both in Cluster 5) was recently characterized, allowing the identification of novel regulatory complexes and stressing the potential importance of sub-networks to regulate cell pluripotency [55]. Also, some of these genes are re-expressed 10 days after LIF withdrawal, therefore becoming LIF responsive in these differentiated cells. In addition, the silencing of *Oct4 *or *Nanog *decreases the expression of many of these genes together, potentially explaining why the individual knock-down of some of these *Pluri *genes does not affect cell pluripotency.

We have identified genes whose expression depends of *Oct4 *only (like *Irak3*, *Susd2*) or of both *Oct4 *and *Nanog *(like *Esrrb, Fzd5, Ly6 *or *Sod2*) or of none of them. Genes whose expression is induced by *Oct4 *(including *Gjb3, Gjb5 *and *Cd9*) or *Nanog *silencing (*Ceacam1*), have also been identified (data sumarized in Figure 10). However, for unknown reasons, these genes were almost not expressed in pluripotent cells used in the study of Loh et al., [19] while highly expressed in the CGR8 cells used in our study and could be among genes whose expression fluctuates between various types of ES cells, as already described [76].

### 24 h without LIF: a critical window for cell fate choices

In this study, we also demonstrated that upon LIF withdrawal, cells go through a transition phase in which the expression of a new set of genes is modulated. Hierarchical clustering analysis was of fundamental importance for the characterization of two types of gene clusters, which displayed "high-low-high" (Figure 3A, cluster 2) or "low-high-low" (Figure 3A, cluster 7) expression levels in the three cell fates analysed "pluripotent, reversibly and irreversibly committed". Some of these genes, (Table 5, see additional file 1), from the Jumonji family, encoding for specific Histone demethylase, have recently been reported as being essential for the maintenance of ES cell pluripotency [77]. In addition, among genes from cluster 7, transcription factors (*Tcfec*, *Zfp532*) and chromatin regulators (*Bhc80*, *Smarca2 *and *Tle4*) might be critical for the regulation of the expression of genes of cluster 2 (*Mapk13*, *Hspb8*, *Vim *and *Jundm2*) [78]. The functional role of these new gene clusters, to drive the transition from pluripotent to irreversible differentiation, will be a future goal.

## Conclusion

Our work has provided a novel global view of LIF reponses in mES cells and in their early-derived derivatives and allowed the identification of various sets of LIF-induced (*Lifind*) and Pluripotency (*Pluri*) genes. We have also characterized sub-networks of genes whose expression is modulated while the master knot (composed of OCT4, SOX2 and NANOG) is still expressed, and which are partly under *Oct4 *and/or *Nanog *control. Furthermore, our study has led to the identification of gene clusters with atypical expression profiles, which desserve to be studied in details for their potential function in ES cell physiology. The recent demonstration that mouse ES cells are heterogeneous for the expression of different genes, like *Rex1 *[79], *Nanog *[53] and many others characterized by in situ hybridization [54], along with the identification of new genes from this study, open new roads for understanding and controlling ES cell plasticity.

## Methods

### Cell culture, treatments for microarrays and reagents

The CGR8 (feeder free) ES cell line (from 129SV mouse) was grown in DMEM, high glucose (Gibco), supplemented with 0.1 mM β2Mercaptoethanol, 10% Foetal Bovine Serum (FBS), 400 μg/ml gentamycin and human LIF (500 pM). Cell medium was changed every other days.

For microarray experiments, plated ES cells were diluted at 10^5 ^cell/ml in ES cell medium with or without LIF. The plating efficiency was similar in the presence and absence of LIF and cells have not been diluted during the 48 h of the experiment. At 24 or 48 h upon LIF withdrawal, cells were treated for 25 min., with medium containing FBS or FBS and LIF, before harvesting. Cells grown in the complete medium with LIF were treated similarly after 48 h of cell growth.

The anti P-STAT3 Tyr705 (Cell signalling), P-STAT3 Ser 727 (gift from Dr. Frank, [80], anti-STAT3 (F-2, Santa Cruz), anti-OCT4 (Abcam) and anti-ERK2 (Santa Cruz) antibodies were used as recommended by the manufacturer.

For the differentiation of CGR8 ES cells by LIF withdrawal (embryoid body formation), hanging drop protocol was executed as described previously [81]. Briefly, an ES cell suspension of 2.5 × 10^4 ^cells/ml was prepared in Iscove's modified Dulbecco's Medium (IMDM) supplemented with 20% FBS, 1% non-essential amino acids (vol/vol), 2 mM L-glutamine, and 100 μM β-ME. Of this ES cell suspension, 20 μl was spotted on the inside of the upper lid of a 10 cm bacteriologic dish and then covered over its bottom dish containing 5 ml phosphate-buffered saline. On day 2, the formed multicellular Embryoid Bodies (EBs) were transferred into suspension in a new dish with IMDM supplemented with 20% FBS, 1% non-essential amino acids (vol/vol), 2 mM L-glutamine, and 100 μM β-ME. On day 7, the EBs were plated on gelatin coated tissue culture dishes. RNA was isolated from ES cells (maintained with LIF, d0) and from differentiating EBs [cultured in the absence of LIF for one day (d1) to day 10 (d10)] on an exact regular interval of 24 hours.

### Microarray experiments: Hybridization and data analysis

Total RNAs were prepared with the Qiagen column kit (Qiagen) and treated with DNAse (5 U/100 μg RNA, Sigma). Biotinylated cRNA was prepared according to the standard Affymetrix protocol (Expression Analysis Technical Manual, 1999; Affymetrix). In brief, double-stranded cDNA was synthesized from 10 μg total RNA using the SuperScript Choice System from InVitrogen and the Affymetrix T7-(dT)_24 _primer which contains a T7 RNA polymerase promoter attached to a poly-dT sequence. Following phenol/chloroform extraction and ethanol precipitation, the cDNA was transcribed into biotin-labeled cRNA using the Retic Lysate IVT™ kit (Ambion Inc., Woodward Austin, TX, USA). The cRNAs produced were purified using RNeasy kit (Qiagen) and fragmented to an average size of 30–50 bases according to Affymetrix recommendations. 15 μg of fragmented cRNAs were hybridized for 16 hr at 45°C on the Mouse Genome 430 2.0 Array. This array integrates 45101 probe sets, including 26275 sequences characterised in Entrez databases and 5069 anonymous ESTs. Arrays have been washed and stained in the Affymetrix Fluidics Station 450 and further scanned using the Affymetrix Gene-Chip scanner 30007 G. The image data were analyzed with the GeneChip^® ^Operating Software (GCOS) using Affymetrix default analysis settings. Arrays – after passing the quality control – have been commonly normalized by the log scale robust multi-array analysis (RMA, [49]). After outlier removal using the Nalimov test at p < 0.001, a parametric ANOVA (F-test) has been applied to get global expression differences between the six different conditions. Multiple testing correction has been performed using the Benjamini-Hochberg procedure [50]. Hierarchical clustering of expression values of probe sets highly significant, differentially expressed in the ANOVA (n = 292; pvalue < 10^-6 ^and FDR < 1,48. 10^-4^) was performed by using Cluster version 2.11 [52] applying mean-centering and normalization of genes and arrays before average linkage clustering with uncentered correlation. To detect distinct expression differences between the growth conditions, paired Student t-tests of transcripts differentially expressed in ANOVA (FDR < 0,05) have been applied. Student t-test p-values and fold changes between the conditions, averages and standard deviations for each condition have been documented.

### Heatmapper analysis

Heatmapper tool allows a graphical representation of the level of expression of many selected genes across a number of samples from various experimental conditions, as they have been obtained with similar DNA microarrays (eg: A430 2.0) [82]. The mean "signal" value from 3 independent experiments obtained from Affymetrix DNA analysis [(arrays A430 2.0: d0 to d10 of LIF withdrawal kinetic on embryoid bodies, (Sachinidis, A. et al, unpublished results)] have been compared with our current data analysis results. A Web-based tool that allows i) quick browsing of user-provided gene sets, ii) standard hierarchical clustering [52] and iii) heatmap-visualisation across multiple data sets, has been created and used for performing this study. This data analysis environment is currently being developped further into a systematic mouse stem cell data analysis atlas (R. Kolde and J. Vilo, unpublished results). Similarly, samples from Affymetrix data of Loh et al, [19], available at GEO data base under the access number: GSE4189, have been added in the interface created, as a way to perform analyses as shown in figures 7.

### Cell lysates, Western blots and Band Shift experiments

Plated cells were rinced twice with room temperature PBS and lysed directly in mild RIPA buffer (PBS, 1% triton, 1% NP40, 0.05% SDS, 1 μg/ml protease inhibitor cocktail, 1 mM Pefabloc, 20 mM NaF, 1 mM Na vanadate) and centrifuged 10 min at 10000 rpm. Cell lysates were resolved by SDS-PAGE and electro-transferred onto nitrocellulose membranes. Proteins were reacted with the different antibodies, as recommended by the manufacturers. Band shift experiments have been performed with nuclear cell lysates as described [38], with the SIE DNA probe, a high affinity DNA binding site for STAT3. Band shifs were performed in the presence of a 100× molar excess of unlabelled wild type (wt) or mutated (m) competitor oligonucleotides encompassing the SIE sequence.

### Semi-quantitative RT-PCR and RT-qPCR

Total RNAs from adherent ES cells were prepared with the Qiagen column kit (Qiagen) and treated with DNAse (5 U/100 μg RNA, Sigma). Total RNAs (5 μg) were reverse-transcribed (RT) with random hexameric primers and the MLV Reverse Transcriptase (Sigma). The RT reaction products were used for PCR with specific primer sets as described [48]. Sequences of primers used for all the genes tested in semi-quantitative RT-PCR (shown in Figure 3) are in Additional file 6.

Quantification, of the extend of Knock-down and of the expression of differentiation markers (see Additional file 5), was performed by RT-qPCR with the MX4000 system (Stratagene) as previously described [83]. The relative expression of the genes of interest was deduced of the Ct using the ΔΔCt method with *Hprt *house-keeping gene as the reference. Sequences of primers used in RTq-PCR are in Additional file 7.

The validation of the expression of LIF cytokine and of its receptors by RT-qPCR analysis (shown in Figure 5C) was performed with the ABI Prism 7500 Fast System (Applied Biosystems, Foster City, CA). 1 μg total mRNAs of ES cells and of EBs, obtained from 3 independent experiments (biological triplicates), were reverse transcribed with ThermoScript™ Reverse Transcriptase (Invitrogen, Karlsruhe, Germany). Then real-time PCR was performed with technical triplicates for every sample using TaqMan Gene Expression Assays (Applied Biosystems, Foster City, CA). The Gene Expression Assays used were *Lif *(Mm00434761_m1), *Lif receptor (gp190) *(Mm00442940_m1), *Interleukin 6 signal transducer (gp130) *(Mm00439668_m1) and *Gapdh *(Mm99999915_g1). C_t _values of each qPCR reaction from the target gene were normalized with the respective C_t _values of the housekeeping gene, *Gapdh*, that ran in the same reaction plate to obtain the Δ C_t _value. The fold change was calculated as follows: fold change = 2^-(ΔCt gene1 - ΔCt gene2)^. The ΔC_t _of the gene in the sample with the lowest expression is used as ΔCt gene2 to calculate the fold change using the above formula. The resulting fold change is expressed as mean ± SD.

### RNAi experiments

Endoribonuclease-prepared short interfering RNA (esiRNA) was synthesized according to the protocol described in [57]. Briefly, selected transcripts were analysed with the software program Deqor [84] to identify the best region for esiRNA synthesis. Primers with appending T7-RNA polymerase-tags were designed and used to amplify gene specific PCR products of about 500 bp in length. These fragments were used to generate dsRNA that was subsequently digested to esiRNAs with *E. coli *RNaseIII. The esiRNA was purified and used to transfect ES cells using Lipofectamine 2000. ES cells were plated in complete medium without antibiotics, on gelatinized 24-wells dishes at day 0, to achieve 60–70% confluency on the day of transfection (day 1). 150 ng of esiRNA (diluted in qsp 50 μl of serum-free DMEM), was mixed with 1 μl of Lipofectamine 2000 (diluted in qsp 50 μl of serum-free DMEM). The mixtures were incublated at room temperature for 20 min. and then added to freshly trypsinised ES cell culture, in suspension in 400 μl of complete medium (with serum and LIF) without antibiotics. Medium was changed on day 2 and cells were re-transfected on day 3. Cells were harvested for RNA preparation or fixed on day 5, 10 min. at 4°C in 2% formaldehyde/0.2% glutaraldehyde in PBS and stained with the Alkaline Phosphatase kit (Sigma-Aldrich, 86R-1KT) according to the manufacturer.

Primer sequences used for esiRNA synthesis are available on request.

## Abbreviations

BMP: Bone Morphogenetic Protein; Car4: Carbonic anhydrase 4; CREB: cAMP Responsive Element Binding; Dapp1: Dual adaptator for PY and PI3K; Dnmt3a: DNA methyltransferase 3a; Dst: Dystonin; ERK: Extracellular signal Regulated Kinase; EB: Embryoid Body; EST: Expressed Sequence Tag; esiRNA: endoribonuclease-prepared short interfering (esi) RNA; FBS: Fœtal Bovine Serum; Ier3: Immediate early response 3; Irak3: Interleukin-1 receptor-associated kinase 3; Lef1: Lymphoid enhancer binding factor 1; LIF: Leukemia Inhibitory Factor; Ly6g6e (Ly6): Lymphocyt antigen 6 complex; mES: mouse Embryonic Stem; Oct4: Octamer 4; Pak1: P21 (cdkn1a)-activated kinase 1; PI3K: Phosphatidyl-Inositol 3-Kinase; Pabpc1: PolyA binding protein, cytoplasmic1; Plscr1: Phospholipid scramblase 1; RSK: Ribosomal S6 Kinase; Susd2: Sushi domain containing 2; Socs3: Suppressor of cytokine signalling 3; Sox2: Sry-related high mobility group (HMG)-box protein-2; STAT3: Signal Transducer and Activator of Transcription 3; Tcfcp2l1 (Tcf): Transcription factor CP2-like 1.

## Authors' contributions

MT, CS, BG, XG participated in cell culture, RNA preparations for affymetrix experiments, validation of the data and functional tests, LD, FB provided the esiRNA materials and elaborated the transfection procedure for esiRNA transfection, MXD, AS, JH provided data for the EBs experiments and did validation of expression profiles of LIF and LIFR genes, OH, NB, HS performed the Affymetrix experiments, the statistical analyses and the cluster analysis, RK, JV provided the WEB site for heatmapper analysis, HB conceived the global affymetrix strategy, designed experiments, organized the coordination and wrote the manuscript. All authors read, corrected and approved the final manuscript.

## Supplementary Material

Additional file 1 **Tables 1 to 7: listing of significant regulated genes following t-test analyses.** Click here for file

Additional file 2 **Characterisation of *Stat3Loc: *probe set number: 1459961_a_at.** Localisation of the sequence corresponding to this probe set, on the mouse genome. Click here for file

Additional file 3 **Complete list of genes shown in the Figure **3A. Click here for file

Additional file 4 **Morphological changes of Alkaline phosphatase stained cells in various cell growth conditions (LIF withdrawal kinetic and knock-down of *Oct4*, *Stat3 *and *Ier3 *in the presence of LIF).** Click here for file

Additional file 5 **Effects of the knock-down of a selection of *Lifind *and *Pluri *genes on the expression level of various committed and differentiation markers.** Click here for file

Additional file 6 **List of primers used for semi-quantitative RT-PCR analysis.** Click here for file

Additional file 7 **List of primers used for RT-qPCR experiments.** Click here for file
