# Supplementary material for: Three LIF-dependent signatures and gene clusters with atypical expression profiles, identified by transcriptome studies in mouse ES cells and early derivatives
Source: BMC Genomics. 2009 Feb 9;10:73. doi: 10.1186/1471-2164-10-73 (PMC2674464; doi:10.1186/1471-2164-10-73)
Supplement: Additional file 1 — Tables 1 to 7: listing of significant regulated genes following t-test analyses. [file 1471-2164-10-73-S1.pdf]

Table 1

| Probe set | p-value | fc | Gene Symbol | Gene Title | Gene Ontology Biological Process |
|---|---|---|---|---|---|
| | Samples 3-4 | | | | |
| ***LIF-induced genes*** | | | | | |
| 1456212_x_at | 2,1214E-06 | 26,69 | Socs3 | suppressor of cytokine signaling 3 | 7242 // intracellular signaling cascade // inferred from electronic annotation /// 1558 // regulation of cell growth // inferred from electronic |
| 1455899_x_at | 9,61616E-06 | 25,71 | Socs3 | suppressor of cytokine signaling 3 | 7242 // intracellular signaling cascade // inferred from electronic annotation /// 1558 // regulation of cell growth // inferred from electronic |
| 1416576_at | 3,64506E-06 | 16,83 | Socs3 | suppressor of cytokine signaling 3 | 7242 // intracellular signaling cascade // inferred from electronic annotation /// 1558 // regulation of cell growth // inferred from electronic |
| 1423100_at | 7,03917E-05 | 11,06 | Fos | FBJ osteosarcoma oncogene | 8151 // cell growth and/or maintenance // inferred from electronic annotation /// 7399 // neurogenesis // inferred from mutant phenotype |
| 1415899_at | 2,28315E-05 | 8,66 | Junb | Jun-B oncogene | 8151 // cell growth and/or maintenance // inferred from direct assay /// 74 // regulation of cell cycle // inferred from direct assay /// 6355 |
| 1459961_a_at | 5,41643E-06 | 5,41 | Stat3Loc | --- | --- |
| 1452519_a_at | 3,03454E-06 | 4,59 | Zfp36 | zinc finger protein 36 | 6402 // mRNA catabolism // inferred from sequence or structural similarity |
| 1417065_at | 0,001515205 | 4,06 | Egr1 | early growth response 1 | 6355 // regulation of transcription, DNA-dependent // inferred from mutant phenotype /// 46652 // thymocyte differentiation // inferred fro |
| 1427683_at | 0,001121372 | 3,45 | Egr2 | early growth response 2 | 42552 // myelination // inferred from mutant phenotype /// 6355 // regulation of transcription, DNA-dependent // inferred from mutant ph |
| 1458308_at | 0,001864448 | 2,41 | Sbno2 | cDNA sequence BC019206 | --- |
| 1416442_at | 0,009872621 | 2,26 | Ier2 | immediate early response 2 | --- |
| 1417483_at | 0,000599049 | 2,25 | Nfkbiz | nuclear factor of kappa light polypeptide | 6954 // inflammatory response // inferred from direct assay /// 6355 // regulation of transcription, DNA-dependent // inferred from seque |
| 1419647_a_at | 0,000199306 | 2,04 | Ier3 | immediate early response 3 | --- |
| 1423619_at | 0,000481877 | 2,03 | Rasd1 | RAS, dexamethasone-induced 1 | 7264 // small GTPase mediated signal transduction // inferred from sequence or structural similarity |
| 1443721_x_at | 0,00027872 | 1,99 | Sbno2 | cDNA sequence BC019206 | --- |
| 1447337_at | 0,000435249 | 1,92 | Dapp1 | Dual adaptator for PY and PI3K | PI3K regulator |
| 1439349_at | 4,97613E-05 | 1,78 | Sbno2 | cDNA sequence BC019206 | --- |
| 1438331_at | 0,001060547 | 1,77 | Ypel2 | Yippee-like 2 (Drosophila) | Nucleus |
| 1457824_at | 0,004058413 | 1,66 | Plscr1 | --- | --- |
| 1451739_at | 0,001302309 | 1,58 | Klf5 | Kruppel-like factor 5 | 6355 // regulation of transcription, DNA-dependent // inferred from electronic annotation |
| 1457404_at | 0,004809154 | 1,52 | Nfkbiz | nuclear factor of kappa light polypeptide | 6954 // inflammatory response // inferred from direct assay /// 6355 // regulation of transcription, DNA-dependent // inferred from seque |
| 1458075_at | 0,011830034 | 1,51 | Dst | Dystonin | 7155 // cell adhesion // inferred from electronic annotation /// 7050 // cell cycle arrest // inferred from sequence or structural similarity /// |
| 1444598_at | 0,000468387 | 1,51 | Etv6 | Ets variant 6 (Tel oncogen) | Transcription factor |
| 1441177_at | 0,00069422 | 1,50 | Pabpc1 | Poly A binding protein, cytoplasmic 1 | --- |

| *LIF-repressed genes* | | | | | |
|---|---|---|---|---|---|
| | | | | | |
| 1459137_at | 0,000442857 | -1,92 | --- | --- | --- |
| 1444320_at | 0,013829571 | -1,90 | Ddhd2 | DDHD domain containing 2 | --- |
| 1457304_at | 0,044704995 | -1,68 | --- | --- | --- |
| 1441333_at | 0,037545575 | -1,67 | Trps1 | Trichorhinophalangeal syndrome I (hum | 6355 // regulation of transcription, DNA-dependent // inferred from electronic annotation |
| 1442445_at | 0,010491985 | -1,63 | 2610027H17Rik | RIKEN cDNA 2610027H17 gene | --- |
| 1438824_at | 0,002016909 | -1,62 | Slc20a1 | Solute carrier family 20, member 1 | Receptor activity, phosphate transport, extracellular membrane |
| 1440717_at | 0,014256571 | -1,58 | --- | --- | --- |
| 1443729_at | 0,024234385 | -1,58 | Mtss1 | metastasis suppressor 1 | 7015 // actin filament organization // inferred from direct assay /// 30041 // actin filament polymerization // inferred from direct assay |
| 1457712_at | 0,025091997 | -1,56 | Chd8 | chromodomain helicase DNA binding pr | Embryonic development |
| 1443088_at | 0,03757275 | -1,53 | 9930031P18Rik | RIKEN cDNA 9930031P18 gene | --- |
| 1458031_at | 0,001767302 | -1,52 | B230315F11Rik | RIKEN cDNA B230315F11 gene | 6810 // transport // inferred from sequence or structural similarity |
| 1438896_at | 0,042316337 | -1,51 | DNajc6 | Adult male pituitary gland cDNA | Phosphatase activity |

| Table 2 | | | | | |
|---|---|---|---|---|---|
| | | | | | |
| Probe set | pvalue | fc | Gene Symbol | Gene Title | Gene Ontology Biological Process |
| | Samples 5-6 | | | | |
| ***LIF-induced genes*** | | | | | |
| 1456212_x_at | 6,56769E-06 | 17,32 | Socs3 | suppressor of cytokine signaling 3 | 7242 // intracellular signaling cascade // inferred from electronic annotation /// 1558 // regulation of cell growth // inferred fror |
| 1455899_x_at | 1,72347E-05 | 16,97 | Socs3 | suppressor of cytokine signaling 3 | 7242 // intracellular signaling cascade // inferred from electronic annotation /// 1558 // regulation of cell growth // inferred fror |
| 1416576_at | 4,24304E-05 | 9,23 | Socs3 | suppressor of cytokine signaling 3 | 7242 // intracellular signaling cascade // inferred from electronic annotation /// 1558 // regulation of cell growth // inferred fror |
| 1423100_at | 0,000524225 | 4,44 | Fos | FBJ osteosarcoma oncogene | 8151 // cell growth and/or maintenance // inferred from electronic annotation /// 7399 // neurogenesis // inferred from mutant |
| 1415899_at | 0,000428514 | 3,41 | Junb | Jun-B oncogene | 8151 // cell growth and/or maintenance // inferred from direct assay /// 74 // regulation of cell cycle // inferred from direct ass |
| 1459961_a_at | 0,001754324 | 3,03 | Stat3Loc | --- | --- |
| 1452519_a_at | 0,000126269 | 2,48 | Zfp36 | zinc finger protein 36 | 6402 // mRNA catabolism // inferred from sequence or structural similarity |
| 1417065_at | 0,004307178 | 2,37 | Egr1 | early growth response 1 | 6355 // regulation of transcription, DNA-dependent // inferred from mutant phenotype /// 46652 // thymocyte differentiation // |
| 1416442_at | 0,027960612 | 1,83 | Ier2 | immediate early response 2 | --- |
| 1427683_at | 0,010852284 | 1,80 | Egr2 | early growth response 2 | 42552 // myelination // inferred from mutant phenotype /// 6355 // regulation of transcription, DNA-dependent // inferred from |
| 1451739_at | 0,000710647 | 1,63 | Klf5 | Kruppel-like factor 5 | 6355 // regulation of transcription, DNA-dependent // inferred from electronic annotation |
| 1423619_at | 0,000183761 | 1,63 | Rasd1 | RAS, dexamethasone-induced 1 | 7264 // small GTPase mediated signal transduction // inferred from sequence or structural similarity |
| 1417395_at | 0,018605917 | 1,54 | Klf4 | Kruppel-like factor 4 (gut) | 6355 // regulation of transcription, DNA-dependent // inferred from electronic annotation |
| 1419647_a_at | 0,000271929 | 1,52 | Ier3 | immediate early response 3 | --- |

# Table 3

| Probe set | pvalue | fc | Gene Symbol | Gene Title | Gene Ontology Biological Process | |
|---|---|---|---|---|---|---|
| | **Samples 3-1** | | | | | |
| 1455899_x_at | 5,68149E-05 | 11,11 | *Socs3* | suppressor of cytokine signaling 3 | 7242 // intracellular signaling cascade // inferred from electronic annotation /// 1558 // regulation of cell growth // inf | |
| 1456212_x_at | 3,69929E-05 | 9,77 | *Socs3* | suppressor of cytokine signaling 3 | 7242 // intracellular signaling cascade // inferred from electronic annotation /// 1558 // regulation of cell growth // inf | |
| 1416576_at | 0,000256498 | 7,08 | *Socs3* | suppressor of cytokine signaling 3 | 7242 // intracellular signaling cascade // inferred from electronic annotation /// 1558 // regulation of cell growth // inf | |
| 1418133_at | 7,88695E-05 | 4,58 | Bcl3 | B-cell leukemia/lymphoma 3 | 6355 // regulation of transcription, DNA-dependent // inferred from sequence or structural similarity | |
| 1429833_at | 0,000836675 | 3,75 | Ly6g6e | lymphocyte antigen 6 complex, locus G6E | Transmembrane receptor/TGFb receptor activity | |
| 1416715_at | 0,000528589 | 3,08 | Gjb3 | gap junction membrane channel protein bet | 7154 // cell communication // inferred from electronic annotation /// 7267 // cell-cell signaling // inferred from electro | |
| 1429377_at | 0,00344185 | 2,99 | 2410004A20Rik | RIKEN cDNA 2410004A20 gene | --- | |
| 1416454_s_at | 0,009344918 | 2,92 | Acta2 /// 0610041G0 | actin, alpha 2, smooth muscle, aorta /// RIK | 7010 // cytoskeleton organization and biogenesis // inferred from electronic annotation /// 7517 // muscle developm | |
| 1430208_at | 0,011955854 | 2,82 | 2410039E07Rik | RIKEN cDNA 2410039E07 gene | --- | |
| 1418569_at | 0,003580766 | 2,77 | 2410043F08Rik | RIKEN cDNA 2410043F08 gene | --- | |
| 1449590_a_at | 0,001071447 | 2,70 | Mras | muscle and microspikes RAS | 7264 // small GTPase mediated signal transduction // inferred from electronic annotation | |
| 1415899_at | 0,010046099 | 2,66 | *Junb* | Jun-B oncogene | 8151 // cell growth and/or maintenance // inferred from direct assay /// 74 // regulation of cell cycle // inferred from | |
| 1424719_a_at | 0,005072824 | 2,59 | Mapt | microtubule-associated protein tau | 7026 // microtubule stabilization // inferred from electronic annotation /// 7017 // microtubule-based process // infer | |
| 1418467_at | 0,002263181 | 2,56 | Smarcd3 | SWI/SNF related, matrix associated, actin d | 18342 // protein prenylation // inferred from sequence or structural similarity | |
| 1449141_at | 0,000642355 | 2,56 | 2410043F08Rik | RIKEN cDNA 2410043F08 gene | --- | |
| 1448213_at | 0,010601507 | 2,56 | Anxa1 | annexin A1 | 50482 // arachidonic acid secretion // inferred from mutant phenotype /// 7049 // cell cycle // inferred from mutant p | |
| 1449204_at | 0,00011577 | 2,51 | Gjb5 | gap junction membrane channel protein bet | 7154 // cell communication // inferred from electronic annotation /// 7267 // cell-cell signaling // inferred from electro | |
| 1431057_a_at | 0,001819217 | 2,49 | 2310046G15Rik | RIKEN cDNA 2310046G15 gene | 6508 // proteolysis and peptidolysis // inferred from electronic annotation | |
| 1417395_at | 0,003998489 | 2,49 | *Klf4* | Kruppel-like factor 4 (gut) | 6355 // regulation of transcription, DNA-dependent // inferred from electronic annotation | |
| 1417013_at | 0,000397666 | 2,38 | Hspb8 * | heat shock 27kDa protein 8 | 6457 // protein folding // inferred from electronic annotation /// 6986 // response to unfolded protein // inferred from | |
| 1418091_at | 0,00315329 | 2,38 | Tcfcp2l1 | transcription factor CP2-like 1 | 122 // negative regulation of transcription from Pol II promoter // inferred from direct assay | |
| 1421830_at | 0,000833105 | 2,28 | Ak3l1 | Adenylate kinase 3-like 1 | Nucleic acid metabolism/ Mitochondria | |
| 1418470_at | 0,000531266 | 2,26 | Yes | Yamaguchi sarcoma viral (v-yes) oncogene | 8151 // cell growth and/or maintenance // inferred from electronic annotation /// 15758 // glucose transport // inferre | |
| 1439757_s_at | 0,000335774 | 2,20 | Epha4 | Eph receptor A4 | 7628 // adult walking behavior // inferred from mutant phenotype /// 7411 // axon guidance // inferred from mutant p | |
| 1433977_at | 0,008966506 | 2,20 | AW536289 | expressed sequence AW536289 | --- | |
| 1417394_at | 0,004902775 | 2,18 | *Klf4* | Kruppel-like factor 4 (gut) | 6355 // regulation of transcription, DNA-dependent // inferred from electronic annotation | |
| 1434500_at | 0,00191481 | 2,14 | Ttyh2 | Tweety homolog-like 2 (Drsophila) | Integral to membrane | |
| 1450387_s_at | 0,000766493 | 2,11 | Ak3l1 | Adenylate kinase 3-like 1 | Nucleic acid metabolism/ Mitochondria | |
| 1460700_at | 0,001390044 | 2,10 | Stat3 | signal transducer and activator of transcript | 7259 // JAK-STAT cascade // inferred from direct assay /// 6953 // acute-phase response // inferred from electronic | |
| 1423669_at | 0,009401474 | 2,10 | Col1a1 | procollagen, type I, alpha 1 | 7155 // cell adhesion // inferred from electronic annotation /// 6817 // phosphate transport // inferred from electronic | |

| | | | | | | |
|---|---|---|---|---|---|---|
| 1450641_at | 3,91911E-05 | 2,09 | Vim * | vimentin | 45103 // intermediate filament-based process // inferred from mutant phenotype | |
| 1437277_x_at | 0,013512365 | 2,06 | Tgm2 | transglutaminase 2, C polypeptide | 18149 // peptide cross-linking // inferred from electronic annotation /// 6508 // proteolysis and peptidolysis // inferre | |
| 1442434_at | 0,009138947 | 2,05 | D8Ertd82e * | NA segment, Chr 8, ERATO Doi 82, express | --- | |
| 1436926_at | 0,003857986 | 2,02 | Esrrb | estrogen related receptor, beta | 6605 // protein targeting // inferred from sequence or structural similarity /// 6355 // regulation of transcription, DNA | |
| 1422914_at | 0,008086304 | 1,99 | Sp5 | trans-acting transcription factor 5 | 6355 // regulation of transcription, DNA-dependent // inferred from electronic annotation | |
| 1427238_at | 0,008815546 | 1,96 | Fbxo15 | F-box protein 15 | 6512 // ubiquitin cycle // inferred from electronic annotation | |
| 1423786_at | 0,00826878 | 1,96 | 8430410A17Rik | RIKEN cDNA 8430410A17 gene | --- | |
| 1453419_at | 0,00408252 | 1,95 | 2900078C09Rik | RIKEN cDNA 2900078C09 gene | --- | |
| 1437247_at | 0,001893341 | 1,93 | Fosl2 | fos-like antigen 2 | 6355 // regulation of transcription, DNA-dependent // inferred from electronic annotation | |
| 1422965_at | 0,001686477 | 1,93 | Agtrap | angiotensin II, type I receptor-associated pr | 8217 // regulation of blood pressure // inferred from mutant phenotype | |
| 1449254_at | 0,002689271 | 1,91 | Spp1 | secreted phosphoprotein 1 | 7155 // cell adhesion // inferred from electronic annotation /// 1503 // ossification // inferred from electronic annotati | |
| 1421829_at | 0,016071757 | 1,91 | Ak3l1 | Adenylate kinase 3-like 1 | Nucleic acid metabolism/ Mitochondria | |
| 1435040_at | 0,005097718 | 1,91 | Irak3 | interleukin-1 receptor-associated kinase 3 | 6915 // apoptosis // inferred from sequence or structural similarity /// 6468 // protein amino acid phosphorylation // i | |
| 1422937_at | 0,008822928 | 1,90 | Fzd5 | frizzled homolog 5 (Drosophila) | 7186 // G-protein coupled receptor protein signaling pathway // inferred from electronic annotation /// 16055 // Wnt | |
| 1422912_at | 0,002271737 | 1,89 | Bmp4 | bone morphogenetic protein 4 | 30509 // BMP signaling pathway // inferred from direct assay /// 1525 // angiogenesis // inferred from mutant phenc | |
| 1420973_at | 0,005555001 | 1,88 | Arid5b | AT rich interactive domain 5B (Mrf1 like) | 6355 // regulation of transcription, DNA-dependent // inferred from electronic annotation | |
| 1456434_x_at | 0,00652875 | 1,88 | Hspb8 * | heat shock 27kDa protein 8 | 6457 // protein folding // inferred from electronic annotation /// 6986 // response to unfolded protein // inferred from | |
| 1460454_at | 0,004294003 | 1,86 | 2010001H14Rik | RIKEN cDNA 2010001H14 gene | --- | |
| 1449090_a_at | 0,011111787 | 1,86 | Yes | Yamaguchi sarcoma viral (v-yes) oncogene | 8151 // cell growth and/or maintenance // inferred from electronic annotation /// 15758 // glucose transport // inferre | |
| 1437100_x_at | 6,01632E-05 | 1,84 | Pim3 | proviral integration site 3 | 6468 // protein amino acid phosphorylation // inferred from electronic annotation | |
| 1417500_a_at | 0,010379111 | 1,84 | Tgm2 | transglutaminase 2, C polypeptide | 18149 // peptide cross-linking // inferred from electronic annotation /// 6508 // proteolysis and peptidolysis // inferre | |
| 1421929_at | 0,00525225 | 1,81 | Epha4 | Eph receptor A4 | 7628 // adult walking behavior // inferred from mutant phenotype /// 7411 // axon guidance // inferred from mutant p | |
| 1456292_a_at* | 0,005481956 | 1,81 | Vim* | vimentin | 45103 // intermediate filament-based process // inferred from mutant phenotype | |
| 1451123_at | 0,001313395 | 1,79 | C330016O10Rik | RIKEN cDNA C330016O10 gene | 6605 // protein targeting // inferred from sequence or structural similarity | |
| 1450781_at | 0,000705393 | 1,79 | Hmga2 | high mobility group AT-hook 2 | 6323 // DNA packaging // inferred from electronic annotation /// 7001 // chromosome organization and biogenesis ( | |
| 1438684_at | 0,000101018 | 1,78 | Nuak1 | Znuak- SNF1-like kinase 1 | Kinase activity | |
| 1438118_x_at | 0,001001756 | 1,78 | Vim * | vimentin | 45103 // intermediate filament-based process // inferred from mutant phenotype | |
| 1460220_a_at | 0,014210962 | 1,78 | Csf1 | colony stimulating factor 1 (macrophage) | 8151 // cell growth and/or maintenance // inferred from electronic annotation /// 40018 // positive regulation of body | |
| 1417193_at | 0,001234577 | 1,78 | Sod2 | superoxide dismutase 2, mitochondrial | 6801 // superoxide metabolism // inferred from mutant phenotype | |
| 1448819_at | 0,003414435 | 1,76 | Eif2s2 | eukaryotic translation initiation factor 2, sub | 6412 // protein biosynthesis // inferred from electronic annotation /// 6413 // translational initiation // inferred from el | |
| 1438175_x_at | 0,001734614 | 1,75 | Myom2 | myomesin 2 | 6936 // muscle contraction // inferred from physical interaction /// 7517 // muscle development // inferred from sequ | |
| 1429388_at | 0,004540293 | 1,75 | Nanog | Nanog homeobox | 17145 // stem cell division // inferred from direct assay | |
| | | | | | 902 // cellular morphogenesis // inferred from sequence or structural similarity /// 7010 // cytoskeleton organization | |

| Table 4 | | | | | |
|---|---|---|---|---|---|
| | | | | | |
| Probe set | pvalue | fc | Gene Symbol | Gene Title | Gene ontology Biological Process |
| | Samples 5-1 | | | | |
| 1456212_x_at | 4,90167E-05 | -14,21 | *Socs3* | suppressor of cytokine signaling 3 | 7242 // intracellular signaling cascade // inferred from electronic annotation /// 1558 // regulation of cell growth // inf |
| 1455899_x_at | 1,9022E-05 | -13,35 | *Socs3* | suppressor of cytokine signaling 3 | 7242 // intracellular signaling cascade // inferred from electronic annotation /// 1558 // regulation of cell growth // inf |
| 1426858_at | 0,00069156 | -7,86 | Inhbb | Inhibin beta-B | 8151 // cell growth and/or maintenance // inferred from electronic annotation /// 40007 // growth // inferred from elec |
| 1456242_at | 0,008279581 | -7,20 | Esgp | Embryonic stem cell and germ cell specific | --- |
| 1416576_at | 9,88461E-06 | -6,32 | *Socs3* | suppressor of cytokine signaling 3 | 7242 // intracellular signaling cascade // inferred from electronic annotation /// 1558 // regulation of cell growth // inf |
| 1417394_at | 0,002060175 | -5,74 | *Klf4* | Kruppel-like factor 4 (gut) | 6355 // regulation of transcription, DNA-dependent // inferred from electronic annotation |
| 1416529_at | 0,001312135 | -5,70 | Emp1 | epithelial membrane protein 1 | 16049 // cell growth // inferred from sequence or structural similarity |
| 1418133_at | 1,11764E-05 | -5,41 | Bcl3 | B-cell leukemia/lymphoma 3 | 6355 // regulation of transcription, DNA-dependent // inferred from sequence or structural similarity |
| 1425538_x_at | 0,001222694 | -5,34 | Ceacam1 | CEA-related cell adhesion molecule 1 | --- |
| 1417395_at | 0,002966638 | -5,25 | *Klf4* | Kruppel-like factor 4 (gut) | 6355 // regulation of transcription, DNA-dependent // inferred from electronic annotation |
| 1449590_a_at | 0,000317026 | -5,19 | Mras | muscle and microspikes RAS | 7264 // small GTPase mediated signal transduction // inferred from electronic annotation |
| 1418091_at | 0,001331556 | -4,89 | Tcfcp2l1 | transcription factor CP2-like 1 | 122 // negative regulation of transcription from Pol II promoter // inferred from direct assay |
| 1429377_at | 0,000785436 | -4,84 | 2410004A20Rik | RIKEN cDNA 2410004A20 gene | --- |
| 1436905_x_at | 0,001865856 | -4,40 | Laptm5 | lysosomal-associated protein transmembra | --- |
| 1429366_at | 0,003097511 | -4,28 | Lrrc34 | Leucin rich repeat containing 34 | --- |
| 1431416_a_at | 0,003572981 | -4,26 | Jam2 | junction adhesion molecule 2 | --- |
| 1435040_at | 0,000609101 | -4,21 | Irak3 | interleukin-1 receptor-associated kinase 3 | 6915 // apoptosis // inferred from sequence or structural similarity /// 6468 // protein amino acid phosphorylation // i |
| 1436568_at | 0,003603632 | -4,11 | Jam2 | junction adhesion molecule 2 | --- |
| 1437165_a_at | 0,000700267 | -4,10 | Pcolce | procollagen C-proteinase enhancer protein | 6508 // proteolysis and peptidolysis // inferred from direct assay |
| 1436926_at | 0,002052319 | -4,09 | Esrrb | estrogen related receptor, beta | 6605 // protein targeting // inferred from sequence or structural similarity /// 6355 // regulation of transcription, DNA- |
| 1418569_at | 0,001666767 | -4,08 | Fblim1 | Filamin binding LIM 1 protein | --- |
| 1427630_x_at | 0,002191884 | -3,78 | Ceacam1 | CEA-related cell adhesion molecule 1 | Positive regulation of MAPK activity/ Receptor activity |
| 1422458_at | 0,003825208 | -3,76 | Tcl1 | T-cell lymphoma breakpoint 1 | --- |
| 1449408_at | 0,004782744 | -3,68 | Jam2 | junction adhesion molecule 2 | --- |
| 1434025_at | 0,003231977 | -3,64 | --- | --- | --- |
| 1436291_a_at | 0,001913821 | -3,59 | Dpys | dihydropyrimidinase | --- |
| 1416715_at | 0,000235973 | -3,53 | Gjb3 | gap junction membrane channel protein be | 7154 // cell communication // inferred from electronic annotation /// 7267 // cell-cell signaling // inferred from electro |
| 1448029_at | 0,004623186 | -3,49 | Tbx3 | T-box 3 | 7569 // cell aging // inferred from direct assay /// 7275 // development // inferred from electronic annotation /// 1648 |
| 1460682_s_at | 0,000704938 | -3,43 | Ceacam2 | CEA-related cell adhesion molecule 2 | --- |
| 1448433_a_at | 0,000370218 | -3,41 | Pcolce | procollagen C-proteinase enhancer protein | 6508 // proteolysis and peptidolysis // inferred from direct assay |

| | | | | | |
|---|---|---|---|---|---|
| 1455604_at | 0,000902695 | -3,37 | --- | --- | --- |
| 1434917_at | 0,001386305 | -3,34 | Cobl | cordon-bleu | 1843 // neural tube closure // inferred from genetic interaction |
| 1421840_at | 0,000503398 | -3,29 | Abca1 | ATP-binding cassette, sub-family A (ABC1 | 8203 // cholesterol metabolism // inferred from direct assay /// 30301 // cholesterol transport // inferred from direct a |
| 1429833_at | 0,001107136 | -3,29 | Ly6g6e | lymphocyte antigen 6 complex, locus G6E | --- |
| 1424719_a_at | 0,004445954 | -3,26 | Mapt | microtubule-associated protein tau | 7026 // microtubule stabilization // inferred from electronic annotation /// 7017 // microtubule-based process // inferr |
| 1435374_at | 0,000462542 | -3,26 | --- | --- | --- |
| 1449530_at | 0,001257967 | -3,24 | Trps1 | trichorhinophalangeal syndrome I (human) | 6355 // regulation of transcription, DNA-dependent // inferred from electronic annotation |
| 1449141_at | 0,0005966 | -3,23 | Fblim1 | Filamin binding LIM 1 protein | --- |
| 1431417_at | 0,00154475 | -3,23 | Jam2 | junction adhesion molecule 2 | --- |
| 1449090_a_at | 0,001960297 | -3,21 | Yes | Yamaguchi sarcoma viral (v-yes) oncogen | 8151 // cell growth and/or maintenance // inferred from electronic annotation /// 15758 // glucose transport // inferre |
| 1422986_at | 0,002795399 | -3,14 | Esrrb | estrogen related receptor, beta | 6605 // protein targeting // inferred from sequence or structural similarity /// 6355 // regulation of transcription, DNA |
| 1427238_at | 0,005693231 | -3,13 | Fbxo15 | F-box protein 15 | 6512 // ubiquitin cycle // inferred from electronic annotation |
| 1418470_at | 0,004845082 | -3,06 | Yes | Yamaguchi sarcoma viral (v-yes) oncogen | 8151 // cell growth and/or maintenance // inferred from electronic annotation /// 15758 // glucose transport // inferre |
| 1455300_at | 0,005488317 | -3,05 | E130014J05Rik | RIKEN cDNA E130014J05 gene | --- |
| 1422123_s_at | 0,000461485 | -3,05 | Ceacam2 /// Ceacam | CEA-related cell adhesion molecule 2 /// C | --- |
| 1449254_at | 0,001719978 | -3,04 | Spp1 | secreted phosphoprotein 1 | 7155 // cell adhesion // inferred from electronic annotation /// 1503 // ossification // inferred from electronic annotati |
| 1444390_at | 0,005246027 | -3,02 | Prdm14 | PR domain containing 14 | Transcription factor |
| 1430125_s_at | 0,006272053 | -3,00 | Pqlc1 | PQ loop repeat containing 1 | --- |
| 1416808_at | 0,003817271 | -2,95 | Nid1 | nidogen 1 | 7155 // cell adhesion // inferred from electronic annotation /// 7160 // cell-matrix adhesion // inferred from direct ass |
| 1420619_a_at | 0,002084343 | -2,90 | Aes1/2 | amino-terminal enhancer of split | 16055 // Wnt receptor signaling pathway // inferred from electronic annotation /// 16481 // negative regulation of tra |
| 1456521_at | 0,00704939 | -2,86 | --- | --- | --- |
| 1450624_at | 0,001014706 | -2,85 | Bhmt | betaine-homocysteine methyltransferase | 9086 // methionine biosynthesis // traceable author statement |
| 1433735_a_at | 0,003455084 | -2,81 | Tmem64 | Transmembrane protein 64 | --- |
| 1430208_at | 0,000507045 | -2,80 | 2410039E07Rik | RIKEN cDNA 2410039E07 gene | --- |
| 1450494_x_at | 0,000230263 | -2,80 | Ceacam1 | CEA-related cell adhesion molecule 1 | --- |
| 1440739_at | 0,004191212 | -2,79 | Vegfc | vascular endothelial growth factor C | 1525 // angiogenesis // inferred from electronic annotation /// 8151 // cell growth and/or maintenance // inferred fror |
| 1419417_at | 0,003048127 | -2,78 | Vegfc | vascular endothelial growth factor C | 1525 // angiogenesis // inferred from electronic annotation /// 8151 // cell growth and/or maintenance // inferred fror |
| 1418362_at | 0,008414517 | -2,78 | Zfp42 | zinc finger protein 42 | 6355 // regulation of transcription, DNA-dependent // inferred from electronic annotation |
| 1422912_at | 0,000914813 | -2,74 | Bmp4 | bone morphogenetic protein 4 | 30509 // BMP signaling pathway // inferred from direct assay /// 1525 // angiogenesis // inferred from mutant pheno |
| 1423508_at | 0,001766282 | -2,73 | Myst4 | MYST histone acetyltransferase monocytic | 16573 // histone acetylation // inferred from direct assay |
| 1449204_at | 0,000151742 | -2,72 | Gjb5 | gap junction membrane channel protein be | 7154 // cell communication // inferred from electronic annotation /// 7267 // cell-cell signaling // inferred from electro |
| 1435084_at | 0,005389269 | -2,72 | C730049O14Rik | RIKEN cDNA C730049O14 gene | --- |
| 1434286_at | 0,007516694 | -2,72 | Trps1 | trichorhinophalangeal syndrome I (human) | 6355 // regulation of transcription, DNA-dependent // inferred from electronic annotation |
| 1425675_s_at | 0,000944016 | -2,71 | Ceacam1 | CEA-related cell adhesion molecule 1 | --- |

| | | | | | |
|---|---|---|---|---|---|
| 1419288_at | 0,005219055 | -2,70 | Jam2 | junction adhesion molecule 2 | --- |
| 1421375_a_at | 0,009926975 | -2,70 | S100a6 | S100 calcium binding protein A6 (calcyclin | 7049 // cell cycle // inferred from electronic annotation /// 8283 // cell proliferation // inferred from electronic annotat |
| 1420973_at | 0,0010852 | -2,69 | Arid5b | AT rich interactive domain 5B (Mrf1 like) | 6355 // regulation of transcription, DNA-dependent // inferred from electronic annotation |
| 1438214_at | 0,006528131 | -2,67 | Trps1 | trichorhinophalangeal syndrome I (human) | 6355 // regulation of transcription, DNA-dependent // inferred from electronic annotation |
| 1418467_at | 0,005009153 | -2,67 | Smarcd3 | SWI/SNF related, matrix associated, actin | 18342 // protein prenylation // inferred from sequence or structural similarity |
| 1423786_at | 0,000188725 | -2,63 | 8430410A17Rik | RIKEN cDNA 8430410A17 gene | --- |
| 1451021_a_at | 0,000767349 | -2,62 | *Klf5* | Kruppel-like factor 5 | 6355 // regulation of transcription, DNA-dependent // inferred from electronic annotation |
| 1417760_at | 0,002974581 | -2,61 | Nr0b1 | clear receptor subfamily 0, group B, membe | 16481 // negative regulation of transcription // inferred from direct assay /// 6355 // regulation of transcription, DNA- |
| 1423281_at | 0,005197813 | -2,57 | Stmn2 | stathmin-like 2 | 7242 // intracellular signaling cascade // inferred from electronic annotation |
| 1419418_a_at | 0,004558273 | -2,57 | Morc | microrchidia | 7283 // spermatogenesis // inferred from mutant phenotype |
| 1421830_at | 0,004155014 | -2,56 | Ak3l1 | Adenylate kinase 3 alpha-like 1 | Nucleic acid metabolism |
| 1430781_at | 0,00067255 | -2,56 | Ak7 | adenylate kinase 7 | --- |
| 1454974_at | 0,006595416 | -2,55 | Ntn1 | netrin 1 | 6915 // apoptosis // inferred from electronic annotation /// 7411 // axon guidance // inferred from mutant phenotype |
| 1429525_s_at | 0,004447781 | -2,54 | Myo1f | myosin IF | 7010 // cytoskeleton organization and biogenesis // inferred from electronic annotation |
| 1433596_at | 0,007070887 | -2,53 | Dnajc6 | DnaJ (Hsp40) homolog, subfamily C, mem | --- |
| 1436419_a_at | 0,008144789 | -2,53 | 1700097N02Rik | RIKEN cDNA 1700097N02 gene | --- |
| 1449187_at | 0,004908424 | -2,50 | Pdgfa | platelet derived growth factor, alpha | 30036 // actin cytoskeleton organization and biogenesis // inferred from direct assay /// 8151 // cell growth and/or m |
| 1426587_a_at | 0,002388451 | -2,49 | Stat3 | signal transducer and activator of transcrip | 7259 // JAK-STAT cascade // inferred from direct assay /// 6953 // acute-phase response // inferred from electronic |
| 1429388_at | 0,005157615 | -2,48 | Nanog | Nanog homeobox | 17145 // stem cell division // inferred from direct assay |
| 1451835_at | 0,000457924 | -2,47 | Sox21 | SRY-box containing gene 21 | 6355 // regulation of transcription, DNA-dependent // inferred from direct assay |
| 1445669_at | 0,007603938 | -2,44 | Spry4 | sprouty homolog 4 (Drosophila) | 7275 // development // inferred from electronic annotation /// 9966 // regulation of signal transduction // inferred fron |
| 1424067_at | 0,001793668 | -2,43 | Icam1 | intercellular adhesion molecule | 7155 // cell adhesion // inferred from direct assay /// 16337 // cell-cell adhesion // inferred from electronic annotatio |
| 1453419_at | 0,000938459 | -2,42 | 2900078C09Rik | RIKEN cDNA 2900078C09 gene | --- |
| 1437435_at | 0,000408331 | -2,42 | 1700061G19Rik | RIKEN cDNA 1700061G19 gene | 8152 // metabolism // inferred from sequence or structural similarity |
| 1417073_a_at | 0,006763115 | -2,40 | Qk | quaking | 7626 // locomotory behavior // inferred from electronic annotation /// 42692 // muscle cell differentiation // inferred fr |
| 1429021_at | 0,009751397 | -2,40 | Epha4 | Eph receptor A4 | 7628 // adult walking behavior // inferred from mutant phenotype /// 7411 // axon guidance // inferred from mutant p |
| 1438781_at | 0,008461031 | -2,40 | --- | Adult male aorta and vein cDNA, RIKEN fu | --- |
| 1452242_at | 0,005550964 | -2,40 | Cep55 | Centrosomal protein 55 | --- |
| 1432229_a_at | 0,000112905 | -2,39 | Cdyl2 | chromodomain protein, Y chromosome-like | --- |
| 1453683_a_at | 0,005072065 | -2,38 | Cep55 | Centrosomal protein 55 | --- |
| 1422937_at | 0,000885572 | -2,35 | Fzd5 | frizzled homolog 5 (Drosophila) | 7186 // G-protein coupled receptor protein signaling pathway // inferred from electronic annotation /// 16055 // Wnt |
| 1452532_x_at | 0,001729847 | -2,35 | Ceacam1 | CEA-related cell adhesion molecule 1 | --- |
| 1450387_s_at | 0,000552887 | -2,34 | Ak3l1 | Adenylate kinase 3 alpha-like 1 | Nucleic acid metabolism |
| 1429399_at | 0,002536554 | -2,34 | Rnf125 | ring finger protein 125 | --- |

| | | | | | |
|---|---|---|---|---|---|
| 1460700_at | 0,000758697 | -2,34 | Stat3 | signal transducer and activator of transcrip | 7259 // JAK-STAT cascade // inferred from direct assay /// 6953 // acute-phase response // inferred from electronic |
| 1420410_at | 0,002645406 | -2,31 | Nr5a2 | nuclear receptor subfamily 5, group A, mei | 8206 // bile acid metabolism // inferred from mutant phenotype /// 42632 // cholesterol homeostasis // inferred from |
| 1429802_at | 0,004729104 | -2,27 | Dhrs10 | dehydrogenase/reductase (SDR family) me | --- |
| 1427004_at | 0,000660207 | -2,26 | Fbxo2 | F-box only protein 2 | 30163 // protein catabolism // inferred from electronic annotation /// 6512 // ubiquitin cycle // inferred from electronic |
| 1457445_at | 0,003639341 | -2,26 | Trps1 | Trichorhinophalangeal syndrome I (human | 6355 // regulation of transcription, DNA-dependent // inferred from electronic annotation |
| 1436932_at | 0,009428344 | -2,24 | Grhl3 | Grainy head-like 3 (Drosophila) | Transcription factor/ ectoderm/ Epiderm/ wound healing |
| 1423280_at | 0,00200439 | -2,24 | Stmn2 | stathmin-like 2 | 7242 // intracellular signaling cascade // inferred from electronic annotation |
| 1454984_at | 0,004898549 | -2,23 | --- | --- | --- |
| 1451782_a_at | 0,001654787 | -2,22 | Slc29a1 | solute carrier family 29 (nucleoside transpo | 15858 // nucleoside transport // inferred from direct assay /// 6810 // transport // inferred from sequence or structura |
| 1438223_at | 0,001358643 | -2,21 | Vps54 | Vacuolar protein sorting 54 (Yeast) | Protein binding |
| 1432227_at | 0,008582055 | -2,19 | Suv39h1 | suppressor of variegation 3-9 homolog 1 (I | 6323 // DNA packaging // traceable author statement /// 6333 // chromatin assembly or disassembly // inferred from |
| 1434283_at | 0,00164546 | -2,19 | Arid5b | AT-rich interacting domain 5B | --- |
| 1450929_at | 0,00373745 | -2,17 | Zfp57 | zinc finger protein 57 | 122 // negative regulation of transcription from Pol II promoter // inferred from direct assay /// 6355 // regulation of t |
| 1422965_at | 0,000412674 | -2,14 | Agtrap | angiotensin II, type I receptor-associated p | 8217 // regulation of blood pressure // inferred from mutant phenotype |
| 1431786_s_at | 0,003706514 | -2,14 | 1190003J15Rik | RIKEN cDNA 1190003J15 gene | 6810 // transport // inferred from electronic annotation |
| 1443621_at | 0,008138386 | -2,11 | --- | --- | --- |
| 1438474_at | 0,000593221 | -2,11 | Ankrd 35 | Ankyrin repeat domain 5 | --- |
| 1448610_a_at | 0,003206376 | -2,10 | Sod2 | superoxide dismutase 2, mitochondrial | 6801 // superoxide metabolism // inferred from mutant phenotype |
| 1417804_at | 0,007629084 | -2,10 | Rasgrp2 | RAS, guanyl releasing protein 2 | 7242 // intracellular signaling cascade // inferred from sequence or structural similarity /// 7264 // small GTPase me |
| 1420361_at | 0,002852296 | -2,09 | Slc11a1 | solute carrier family 11 (proton-coupled div | 6826 // iron ion transport // inferred from electronic annotation /// 6810 // transport // inferred from electronic annota |
| 1438672_at | 0,005246238 | -2,08 | Parvb | Parvin beta | Actin binding/ Cell adhesion |
| 1423686_a_at | 0,000223554 | -2,08 | 1110020C13Rik | Prr13 | Prolin rich 13 |
| 1441921_x_at | 0,001201237 | -2,08 | Esrrb | estrogen related receptor, beta | 6605 // protein targeting // inferred from sequence or structural similarity /// 6355 // regulation of transcription, DNA- |
| 1417193_at | 0,000412356 | -2,06 | Sod2 | superoxide dismutase 2, mitochondrial | 6801 // superoxide metabolism // inferred from mutant phenotype |
| 1431979_at | 0,00159004 | -2,06 | 4930444M15Rik | RIKEN cDNA 4930444M15 gene | --- |
| 1448469_at | 0,003256379 | -2,04 | Nid1 | nidogen 1 | 7155 // cell adhesion // inferred from electronic annotation /// 7160 // cell-matrix adhesion // inferred from direct ass |
| 1454946_at | 0,002289226 | -2,04 | Mybl2 | myeloblastosis oncogene-like 2 | 8151 // cell growth and/or maintenance // inferred from electronic annotation /// 74 // regulation of cell cycle // inferr |
| 1453063_at | 0,000449131 | -2,04 | Cltb | clathrin, light polypeptide (Lcb) | --- |
| 1454709_at | 0,000666778 | -2,04 | Tmem 64 | Transmembrane protein 64 | --- |
| 1448890_at | 0,004449204 | -2,02 | Klf2 | Kruppel-like factor 2 (lung) | 45941 // positive regulation of transcription // inferred from direct assay /// 6355 // regulation of transcription, DNA-c |
| 1448845_at | 0,0039053 | -2,02 | Rpp25 | ribonuclease P 25 subunit (human) | --- |
| 1415899_at | 0,000175953 | -2,01 | *Junb* | Jun-B oncogene | 8151 // cell growth and/or maintenance // inferred from direct assay /// 74 // regulation of cell cycle // inferred from c |
| 1444531_at | 0,001478207 | -2,01 | Sod2 | Superoxyde Dismutase 2 | --- |
| 1451123_at | 0,001730809 | -2,01 | C330016O10Rik | RIKEN cDNA C330016O10 gene | 6605 // protein targeting // inferred from sequence or structural similarity |

| | | | | | |
|---|---|---|---|---|---|
| 1460235_at | 0,001771319 | -2,00 | Scarb2 | scavenger receptor class B, member 2 | 7155 // cell adhesion // inferred from sequence or structural similarity |
| 1449231_at | 0,002307877 | -1,99 | Zfp296 | zinc finger protein 296 | --- |
| 1450860_at | 0,000610729 | -1,99 | Lap3 | leucine aminopeptidase 3 | 6508 // proteolysis and peptidolysis // inferred from electronic annotation |
| 1428092_at | 0,002493174 | -1,98 | Cdc5l | cell division cycle 5-like (S. pombe) | 910 // cytokinesis // inferred from electronic annotation /// 6605 // protein targeting // inferred from sequence or stru |
| 1423187_at | 0,00224195 | -1,98 | Gabarapl2 | gamma-aminobutyric acid (GABA-A) recep | 6891 // intra-Golgi transport // inferred from sequence or structural similarity /// 6886 // intracellular protein transpor |
| 1444779_s_at | 0,00648575 | -1,97 | Zfp59 | zinc finger protein 59 | --- |
| 1457435_x_at | 0,002031946 | -1,96 | Myom2 | myomesin 2 | 6936 // muscle contraction // inferred from physical interaction /// 7517 // muscle development // inferred from sequ |
| 1434307_at | 0,005028081 | -1,95 | Tmem 64 | Transmembrane protein 64 | --- |
| 1419896_at | 0,002145264 | -1,94 | BB001228 | Expressed sequence BB001228 | --- |
| 1451416_a_at | 0,002590676 | -1,94 | Tgm1 | transglutaminase 1, K polypeptide | 9887 // organogenesis // inferred from mutant phenotype /// 19538 // protein metabolism // traceable author statem |
| 1454666_at | 0,007951118 | -1,93 | Klf3 | Kruppel-like factor 3 (basic) | 6355 // regulation of transcription, DNA-dependent // inferred from electronic annotation |
| 1417656_at | 0,008468401 | -1,93 | Mybl2 | myeloblastosis oncogene-like 2 | 8151 // cell growth and/or maintenance // inferred from electronic annotation /// 74 // regulation of cell cycle // inferr |
| 1434836_at | 0,000635058 | -1,93 | Nfatc2ip | nuclear factor of activated T-cells, cytoplas | 6355 // regulation of transcription, DNA-dependent // inferred from direct assay |
| 1460429_at | 0,000419498 | -1,93 | Cdc5l | cell division cycle 5-like (S. pombe) | 910 // cytokinesis // inferred from electronic annotation /// 6605 // protein targeting // inferred from sequence or stru |
| 1416239_at | 0,00168276 | -1,93 | Ass1 | argininosuccinate synthetase 1 | 6526 // arginine biosynthesis // inferred from electronic annotation /// 50 // urea cycle // inferred from electronic ann |
| 1426511_at | 0,002454152 | -1,93 | Susd2 | sushi domain containing 2 | --- |
| 1428440_at | 0,00695347 | -1,92 | Slc25a12 | solute carrier family 25 (mitochondrial carri | 6810 // transport // inferred from sequence or structural similarity |
| 1425503_at | 0,00370915 | -1,92 | Gcnt2 | glucosaminyl (N-acetyl) transferase 2, I-bra | --- |
| 1426733_at | 0,000228377 | -1,92 | Itpk1 | inositol 1,3,4-triphosphate 5/6 kinase | --- |
| 1449064_at | 0,001820895 | -1,91 | Tdh | L-threonine dehydrogenase | --- |

| Table 5 | | | | | | |
|---|---|---|---|---|---|---|
| | | | | | Genes | |
| | p-value | | | | also expressed | |
| Probe set | Samples 3-1 | fc | Gene Symbol | Gene Title | in irrev. com. Cells | Gene Ontology Biological Process |
| 1436398_at | 0,000698423 | -3,89 | Lef1 | Lymphoid enhancer binding factor 1 | YES | 16055 // Wnt receptor signaling pathway // inferred from electronic annotation /// 42475 // odontogen |
| 1425995_s_at | 0,000139441 | -3,85 | Wt1 | Wilms tumor homolog | YES | 30855 // epithelial cell differentiation // inferred from mutant phenotype /// 1654 // eye morphogenesis |
| 1442655_at | 4,00665E-05 | -3,59 | Dnmt3b | DNA methyltransferase 3B | YES | Imprinting |
| 1436964_at | 7,40368E-05 | -3,59 | D7Ertd715e | DNA segment, Chr 7, ERATO Doi 715 | NO | --- |
| 1454734_at | 0,000525552 | -3,50 | Lef1 | lymphoid enhancer binding factor 1 | YES | 16055 // Wnt receptor signaling pathway // inferred from electronic annotation /// 42475 // odontogen |
| 1421299_a_at | 0,002056782 | -3,37 | Lef1 | lymphoid enhancer binding factor 1 | YES | 16055 // Wnt receptor signaling pathway // inferred from electronic annotation /// 42475 // odontogen |
| 1439260_a_at | 0,001747708 | -3,29 | Enpp3 | ectonucleotide pyrophosphtase/phosphod | NO | 9117 // nucleotide metabolism // inferred from sequence or structural similarity |
| 1441145_at | 0,004056552 | -3,20 | D030065N23Rik | RIKEN cDNA D030065N23 gene | NO | --- |
| 1460038_at | 0,005745518 | -3,19 | Oct6/Tst-1 | | YES | --- |
| 1427133_s_at | 2,81388E-05 | -3,17 | Lrp2 | Low density lipoprotein receptor-related p | YES | 6898 // receptor mediated endocytosis // inferred from mutant phenotype /// 6766 // vitamin metabolis |
| 1452320_at | 4,99127E-05 | -3,14 | Lrp2 | Low density lipoprotein receptor-related p | YES | 6898 // receptor mediated endocytosis // inferred from mutant phenotype /// 6766 // vitamin metabolis |
| 1438454_at | 0,00378768 | -3,10 | B430203M17Rik | RIKEN cDNA B430203M17 gene | YES | --- |
| 1425926_a_at | 0,003414321 | -2,98 | Otx2 | orthodenticle homolog 2 (Drosophila) | YES | 9952 // anterior/posterior pattern formation // inferred from mutant phenotype /// 45165 // cell fate cor |
| 1443167_at | 0,002470274 | -2,84 | Rnf12 | Ring finger protein 12 | NO | 16567 // protein ubiquitination // inferred from electronic annotation /// 6355 // regulation of transcripti |
| 1454866_s_at | 0,00687868 | -2,78 | Clic6 | chloride intracellular channel 6 | NO | 6821 // chloride transport // inferred from sequence or structural similarity /// 6811 // ion transport // in |
| 1453361_at | 0,008555241 | -2,76 | Hells | Helicase, lymphoid specific | NO | DNA methylation, chromatin silencing/ apoptosis |
| 1439123_at | 0,003963268 | -2,74 | Bhc80 ** | BRAF35/HDAC2 complex | NO | 6355 // regulation of transcription, DNA-dependent // inferred from sequence or structural similarity |
| 1455029_at | 0,001597536 | -2,74 | Kif21a | Kinesin family member 21A | YES | --- |
| 1419639_at | 0,001235348 | -2,74 | Ephb2 | EphrinB2 | YES | --- |
| 1439231_at | 0,001969269 | -2,73 | --- | --- | NO | --- |
| 1437548_at | 0,005915598 | -2,73 | Bicd1 | BicaudalD,homolog1( Drosophila) | NO | --- |
| 1423063_at | 0,005813525 | -2,72 | Dnmt3a | DNA methyltransferase 3A | YES | 6306 // DNA methylation // inferred from direct assay /// 6349 // imprinting // inferred from mutant phe |
| 1460324_at | 0,009058634 | -2,72 | Dnmt3a | DNA methyltransferase 3A | YES | 6306 // DNA methylation // inferred from direct assay /// 6349 // imprinting // inferred from mutant phe |
| 1446526_at | 0,003670747 | -2,70 | 9330199F22Rik | RIKEN cDNA 9330199F22 gene | NO | --- |
| 1460006_at | 0,001272593 | -2,68 | Atbf1 | AT motif binding factor 1 | YES | 6355 // regulation of transcription, DNA-dependent // inferred from electronic annotation |
| 1418391_at | 0,001112074 | -2,67 | Phf21a | PHD finger protein 21A | NO | --- |
| 1443526_at** | 0,004013711 | -2,66 | Bhc80** | BRAF35/HDAC2 complex | NO | 6355 // regulation of transcription, DNA-dependent // inferred from sequence or structural similarity |
| 1436766_at | 0,008667766 | -2,66 | Luc7l2 | LUC7-like 2 (S. cerevisiae) | NO | --- |
| 1459722_at | 0,002234879 | -2,65 | Zswim6 | Zinc finger Zwim domain containing 6 | YES | --- |
| 1444646_at | 0,00192628 | -2,61 | 8430420F16Rik | RIKEN cDNA 8430420F16 gene | NO | --- |

| | | | | | | |
|---|---|---|---|---|---|---|
| 1419204_at | 0,005644524 | -2,60 | Dll1 | delta-like 1 (Drosophila) | YES | 7154 // cell communication // inferred from electronic annotation /// 30154 // cell differentiation // infer |
| 1418390_at | 0,003680635 | -2,59 | Bhc80 ** | BRAF35/HDAC2 complex | NO | 6355 // regulation of transcription, DNA-dependent // inferred from sequence or structural similarity |
| 1455087_at | 0,003031932 | -2,59 | D7Ertd715e | DNA segment, Chr 7, ERATO Doi 715, ex | NO | --- |
| 1419959_s_at | 0,000823195 | -2,56 | C330003B14Rik | RIKEN cDNA C330003B14 gene | NO | 6355 // regulation of transcription, DNA-dependent // inferred from sequence or structural similarity |
| 1436082_at | 0,002787121 | -2,54 | Slc24a5 | Solute carrier family 24, member 5 | NO | --- |
| 1416638_at | 0,002121067 | -2,52 | Sall2 | sal-like 2 (Drosophila) | YES | 6355 // regulation of transcription, DNA-dependent // inferred from electronic annotation |
| 1431633_x_at | 0,001918816 | -2,52 | 4930526L06Rik | RIKEN cDNA 4930526L06 gene | NO | --- |
| 1419638_at | 0,001241028 | -2,52 | Efnb2 | ephrin B2 | YES | 7275 // development // inferred from electronic annotation /// 7399 // neurogenesis // inferred from ele |
| 1439998_at | 0,001823303 | -2,50 | Jmjd1C | Jumonji domain containing 1C, chromatin | NO | --- |
| 1445710_x_at | 0,001143813 | -2,50 | 1110051B16Rik | RIKEN cDNA 1110051B16 gene | NO | --- |
| 1456960_at | 0,007543746 | -2,48 | Adk | Adenosine kinase | NO | 6166 // purine ribonucleoside salvage // inferred from direct assay |
| 1459973_x_at | 0,001467471 | -2,46 | Dpp4 | Dipeptidylpeptidase 4 | NO | --- |
| 1439665_at | 0,009035862 | -2,42 | Gpr23 | G protein-coupled receptor 23 | YES | --- |
| 1454617_at | 0,001761097 | -2,42 | Arrdc3 | arrestin domain containing 3 | NO | --- |
| 1456159_at | 0,008150022 | -2,41 | 2900045N06Rik | RIKEN cDNA 2900045N06 gene | NO | --- |
| 1456862_at | 0,004851524 | -2,40 | Six4 | Sine oculis-related homeobox 4 homolog | NO | Transcription factor/ Development/ muscle/ migration myoblast |
| 1440896_at | 0,001652504 | -2,36 | AA517739 | Expressed sequence AA517739 | NO | --- |
| 1423064_at | 0,000422429 | -2,36 | Dnmt3a | DNA methyltransferase 3A | YES | 6306 // DNA methylation // inferred from direct assay /// 6349 // imprinting // inferred from mutant phe |
| 1454806_at | 0,002106775 | -2,33 | D12Ertd553e | DNA segment, Chr 12, ERATO Doi 553, e | NO | --- |
| 1444320_at | 0,002429669 | -2,33 | Ddhd2 | DDHD domain containing 2 | NO | --- |
| 1455121_at | 0,007693128 | -2,32 | Mlr2** | Mblk1-related protein-2 | NO | 6366 // transcription from Pol II promoter // inferred from direct assay |
| 1440011_at | 0,001286189 | -2,32 | Ext1 | Exostoses (multiple) 1 | NO | 8151 // cell growth and/or maintenance // inferred from electronic annotation /// 7492 // endoderm de |
| 1438802_at | 0,000235708 | -2,32 | Foxp1 | Forkhead box-1 | NO | --- |
| 1418911_s_at | 0,006289806 | -2,32 | Acsl4 | acyl-CoA synthetase long-chain family me | NO | 6631 // fatty acid metabolism // inferred from electronic annotation /// 8152 // metabolism // inferred fr |
| 1426043_a_at | 0,002100261 | -2,32 | Capn3 | calpain 3 | NO | 6508 // proteolysis and peptidolysis // inferred from electronic annotation |
| 1439753_x_at | 0,003818434 | -2,31 | Six4 | Sine oculis-related homeobox 4 homolog | NO | --- |
| 1448733_at | 0,004360239 | -2,31 | Bmi1 | B lymphoma Mo-MLV insertion region 1 | NO | 7420 // brain development // inferred from mutant phenotype /// 8151 // cell growth and/or maintenan |
| 1427242_at | 0,008070486 | -2,30 | Ddx4 | DEAD (Asp-Glu-Ala-Asp) box polypeptide | NO | 7275 // development // inferred from electronic annotation |
| 1435952_at | 0,004658253 | -2,29 | --- | --- | NO | --- |
| 1457072_at | 0,003396674 | -2,28 | Bcl11A | B cell lymphoma 11A | NO | B and T cell differentiation |
| 1458031_at | 0,002796464 | -2,28 | B230315F11Rik | RIKEN cDNA B230315F11 gene | NO | 6810 // transport // inferred from sequence or structural similarity |
| 1427046_at | 0,009888514 | -2,27 | Tcfcp2l3 | transcription factor CP2-like 3 | NO | 45449 // regulation of transcription // inferred from sequence or structural similarity |
| 1441243_at | 0,001147825 | -2,26 | Zfp532 | Zinc finger protein 532 | NO | --- |
| 1418345_at | 0,003981161 | -2,26 | Tnfsf13 | tumor necrosis factor (ligand) superfamily | NO | 16064 // humoral defense mechanism (sensu Vertebrata) // inferred from mutant phenotype /// 6955 |

| | | | | | | |
|---|---|---|---|---|---|---|
| 1451687_a_at | 0,009269916 | -2,25 | Tcf2 | Transcription factor 2 | NO | --- |
| 1442744_at | 0,006993936 | -2,23 | C79248 | expressed sequence C79248 | NO | --- |
| 1452142_at | 0,009383967 | -2,23 | Slc6a1 | solute carrier family 6 (neurotransmitter tr | NO | 6836 // neurotransmitter transport // inferred from electronic annotation /// 6810 // transport // inferred |
| 1447360_at | 0,001298136 | -2,23 | Tsc22d1 | Tsc 22 domain family, member 1 | NO | --- |
| 1427427_at | 0,000729202 | -2,22 | Ryr3 | ryanodine receptor 3 | NO | 6811 // ion transport // inferred from electronic annotation /// 6810 // transport // inferred from electron |
| 1454138_a_at | 0,004823638 | -2,22 | Stk31 | serine threonine kinase 31 | NO | 6468 // protein amino acid phosphorylation // inferred from electronic annotation |
| 1444851_at | 0,002998057 | -2,21 | Zfp532 | Zinc finger protein 532 | NO | --- |
| 1450044_at | 0,000755881 | -2,20 | Fzd7 | frizzled homolog 7 (Drosophila) | YES | 7186 // G-protein coupled receptor protein signaling pathway // inferred from electronic annotation /// |
| 1437217_at | 0,001058179 | -2,20 | Ankrd6 | Ankyrin repeat domain 6 | NO | --- |
| 1455355_at | 0,004336498 | -2,19 | 6030408C04Rik | RIKEN cDNA 6030408C04 gene | NO | --- |
| 1423065_at | 0,001315052 | -2,18 | Dnmt3a | DNA methyltransferase 3A | YES | 6306 // DNA methylation // inferred from direct assay /// 6349 // imprinting // inferred from mutant phe |
| 1456632_at | 0,004211458 | -2,18 | Bcl11A | B cell lymphoma 11A | YES | B and T cell differentiation |
| 1438130_at | 0,008941028 | -2,15 | Taf15 | TAF15 RNA polymerase II, TATA box bin | NO | --- |
| 1452384_at | 0,000843063 | -2,15 | Enpp3 | ectonucleotide pyrophosphatase/phospho | NO | 9117 // nucleotide metabolism // inferred from sequence or structural similarity |
| 1437372_at | 0,004264305 | -2,15 | Cpsf6 | Cleavage and polyA specific factor 6 | NO | --- |
| 1442959_at | 0,005438897 | -2,14 | Birc6 | baculoviral IAP repeat-containing 6 | NO | 6916 // anti-apoptosis // inferred from sequence or structural similarity /// 6915 // apoptosis // inferred |
| 1446550_at | 0,009820767 | -2,14 | Gspt1 | G1 to S phase transition 1 | NO | 82 // G1/S transition of mitotic cell cycle // inferred from sequence or structural similarity /// 8283 // ce |
| 1444615_x_at | 0,000131032 | -2,13 | Cbfa2t1h | CBFA2T1 identified gene homolog (huma | NO | 6355 // regulation of transcription, DNA-dependent // inferred from electronic annotation |
| 1437660_at | 0,006631425 | -2,12 | Nktr | Natural killer tumor recognition sequence | NO | 6457 // protein folding // inferred from electronic annotation |
| 1443729_at | 0,004190799 | -2,12 | Mtss1 | metastasis suppressor 1 | NO | 7015 // actin filament organization // inferred from direct assay /// 30041 // actin filament polymerizati |
| 1427193_at | 0,003225889 | -2,12 | Brd8 ** | bromodomain containing 8 | NO | --- |
| 1459384_at | 0,002721374 | -2,12 | Dnmt3b | DNA methyltransferase 3B | YES | --- |
| 1438701_at | 0,000881439 | -2,11 | B830009D06Rik | RIKEN cDNA B830009D06 gene | NO | --- |
| 1438542_at | 0,008859682 | -2,11 | --- | --- | NO | --- |
| 1427202_at | 0,002113065 | -2,11 | AI256744 | RIKEN cDNA 4833442J19 gene | NO | --- |
| 1456670_at | 0,002007733 | -2,10 | A930007A09Rik | RIKEN cDNA A930007A09 gene | NO | --- |
| 1440910_at | 0,003312345 | -2,10 | Bicd1 | Bicaudal D homolog 1 | NO | --- |
| 1443305_at | 0,00163503 | -2,09 | Sall1 | Sal-like 1 (Drosophila) | NO | 9887 // organogenesis // inferred from mutant phenotype /// 6355 // regulation of transcription, DNA-c |
| 1442671_at | 0,007115851 | -2,08 | Hip2 | Huntingtin-interacting protein 2 | NO | --- |
| 1449947_s_at | 0,006366194 | -2,08 | Atbf1 | AT motif binding factor 1 | NO | 6355 // regulation of transcription, DNA-dependent // inferred from electronic annotation |
| 1437127_at | 0,008074357 | -2,08 | C730040L01Rik | RIKEN cDNA C730040L01 gene | NO | --- |
| 1453133_at | 0,002144548 | -2,08 | Slc25a31 | Solute carrier family 25, member 31 | NO | 6810 // transport // inferred from sequence or structural similarity |
| 1439138_at | 0,005736702 | -2,08 | --- | --- | NO | --- |
| 1419241_a_at | 0,003684106 | -2,06 | Aire ** | autoimmune regulator (autoimmune polye | NO | 6959 // humoral immune response // inferred from mutant phenotype /// 6355 // regulation of transcrip |

| | | | | | | |
|---|---|---|---|---|---|---|
| 1431632_at | 0,004187513 | -2,06 | 4930526L06Rik | RIKEN cDNA 4930526L06 gene | NO | --- |
| 1450292_a_at | 0,000824094 | -2,06 | Hormad1 | Horma domain containing 1 | NO | --- |
| 1430526_a_at | 0,000161766 | -2,05 | Smarca2 ** | SWI/SNF related, matrix associated, actin | NO | 6325 // establishment and/or maintenance of chromatin architecture // traceable author statement /// |
| 1458687_at | 0,006861406 | -2,04 | --- | Gene model 177, (NCBI) | NO | --- |
| 1440806_x_at | 0,003813544 | -2,04 | 5730526G10Rik | RIKEN cDNA 5730526G10 gene | NO | --- |
| 1438349_at | 0,009701756 | -2,04 | BC043476 | CDNA sequence BC043476 | NO | --- |
| 1434558_at | 0,000662873 | -2,04 | Wdr47 | WD repeat domain 47 | NO | --- |
| 1438675_at | 0,000440531 | -2,04 | Sfrs8 | Splicing factor, arginine/serine-rich 8 | NO | 6376 // mRNA splice site selection // inferred from electronic annotation |
| 1454809_at | 0,000571753 | -2,04 | Ncoa7 | Nuclear receptor coactivator 7 | NO | --- |
| 1436203_a_at | 0,009386969 | -2,03 | 1110059G02Rik | RIKEN cDNA 1110059G02 gene | NO | --- |
| 1440764_at | 0,001711533 | -2,03 | Araf | V-ras sarcoma 3611 viral oncogen homolo | NO | --- |
| 1439775_at | 0,005594815 | -2,03 | Brwd3 | Bromo domain and WD repeat domain 3 | NO | --- |
| 1437766_at | 0,008660672 | -2,03 | --- | --- | NO | --- |
| 1456266_at | 0,006943412 | -2,03 | Rpl30 | Ribosomal protein like 30 | NO | 6412 // protein biosynthesis // inferred from electronic annotation |
| 1452309_at | 0,001045822 | -2,02 | Cgnl1 | Cingulin like 1 | NO | --- |
| 1425035_s_at | 0,00280301 | -2,02 | Dnmt3l | DNA (cytosine-5-)-methyltransferase 3-lik | NO | 1701 // embryonic development (sensu Mammalia) // inferred from mutant phenotype /// 6349 // impr |
| 1448665_at | 0,003233149 | -2,02 | Dmd | dystrophin, muscular dystrophy | NO | 7517 // muscle development // inferred from mutant phenotype |
| 1437495_at | 0,005405981 | -2,02 | Mbtps2 | membrane-bound transcription factor prot | NO | 6508 // proteolysis and peptidolysis // inferred from sequence or structural similarity |
| 1436386_x_at | 0,001413203 | -2,01 | --- | --- | NO | --- |
| 1431830_at | 0,006254673 | -2,00 | Zfp329 | RIKEN cDNA 4632409L22 gene | NO | --- |
| 1434272_at | 0,001507326 | -1,99 | Cpeb2 | cytoplasmic polyadenylation element bind | NO | --- |
| 1435222_at | 0,002079249 | -1,99 | Foxp1 | Forkhead box P1 | NO | 16481 // negative regulation of transcription // inferred from direct assay /// 6355 // regulation of trans |
| 1457883_at | 0,009791883 | -1,99 | --- | Adult male aorta and vein cDNA, RIKEN f | YES | --- |
| 1440095_at | 0,009608755 | -1,99 | --- | --- | NO | --- |
| 1438215_at | 0,001332298 | -1,98 | Sfrs3 | splicing factor, arginine/serine-rich 3 (SRp | NO | 6376 // mRNA splice site selection // inferred from electronic annotation /// 398 // nuclear mRNA splic |
| 1455340_at | 0,003087599 | -1,97 | AI852444 | Expressed sequence AI852444 | NO | --- |
| 1438398_at | 0,003679596 | -1,97 | Rnpc2 | RNA binding protein containing 2 | NO | 6397 // mRNA processing // inferred from electronic annotation /// 398 // nuclear mRNA splicing, via s |
| 1438234_at | 0,004270877 | -1,97 | Wdr26 | WD repeat domain 26 | NO | --- |
| 1442566_at | 0,005116018 | -1,97 | --- | --- | NO | --- |
| 1438084_at | 0,003915902 | -1,97 | Adam23 | --- | NO | --- |
| 1447624_s_at | 0,001426344 | -1,97 | Stox2 | Storkhead box2 | NO | --- |
| 1440929_at | 0,007529099 | -1,96 | Ggnbp2 | RIKEN cDNA D330017P12 gene | NO | --- |
| 1450853_at | 0,000558409 | -1,96 | Tle4 ** | transducin-like enhancer of split 4, homolo | NO | 16055 // Wnt receptor signaling pathway // inferred from direct assay /// 7222 // frizzled signaling path |
| 1443088_at | 0,004066847 | -1,95 | 9930031P18Rik | RIKEN cDNA 9930031P18 gene | NO | --- |

| | | | | | | |
|---|---|---|---|---|---|---|
| 1439852_at | 0,004793771 | -1,95 | --- | --- | NO | --- |
| 1435931_at | 0,003178361 | -1,95 | --- | --- | NO | --- |
| 1457712_at | 0,009868308 | -1,94 | Chd8 | chromodomain helicase DNA binding prot | NO | --- |
| 1436153_a_at | 0,003185974 | -1,93 | Zmynd11 | Zinc finger, MYND domain containing 11 | NO | 8151 // cell growth and/or maintenance // inferred from sequence or structural similarity /// 8283 // ce |
| 1440847_at | 0,002446985 | -1,93 | Mtss1 | metastasis suppressor 1 | NO | 7015 // actin filament organization // inferred from direct assay /// 30041 // actin filament polymerizati |
| 1443527_at | 0,009612528 | -1,93 | Terf1 | telomeric repeat binding factor 1 | NO | 7049 // cell cycle // inferred from electronic annotation /// 7001 // chromosome organization and biog |
| 1436240_at | 0,00878518 | -1,93 | Sost | Sclerotin | NO | Ossification/ repressor of BMP pathway |
| 1424458_at | 0,005107134 | -1,92 | Jmjd2c | jumonji domain containing 2C | NO | 6355 // regulation of transcription, DNA-dependent // inferred from sequence or structural similarity |
| 1423066_at | 0,002710222 | -1,92 | Dnmt3a | DNA methyltransferase 3A | YES | 6306 // DNA methylation // inferred from direct assay /// 6349 // imprinting // inferred from mutant phe |
| 1428467_at | 0,008493213 | -1,91 | 1190002A23Rik | RIKEN cDNA 1190002A23 gene | NO | --- |
| 1417850_at | 0,00137721 | -1,91 | Rb1 | retinoblastoma 1 | NO | 45786 // negative regulation of cell cycle // inferred from mutant phenotype /// 122 // negative regulat |
| 1435436_at | 0,003535318 | -1,91 | Epas1 | Endothelial PAS domain binding 1 | NO | --- |
| 1425087_at | 0,004814494 | -1,91 | 2310003F16Rik | RIKEN cDNA 2310003F16 gene | NO | --- |
| 1441429_at | 0,00392271 | -1,91 | Irs4 | Insulin receptor substrate 4 | YES | --- |
| 1455337_at | 0,003911565 | -1,90 | Fgd4 | Fyve, RhoGef and PH domain containing | YES | --- |
| 1457731_at | 0,002911932 | -1,90 | Snapc3 | small nuclear RNA activating complex, po | NO | --- |
| 1438980_x_at | 0,004523629 | -1,90 | 4732466D17Rik | RIKEN cDNA 4732466D17 gene | NO | 6508 // proteolysis and peptidolysis // inferred from sequence or structural similarity |
| | | | | | | |
| ** : | Genes with transient expression in reversibly committed cells | | | | | |
| | (Genes in Cluster 7, Figure 3A) | | | | | |

| Table 6 | | | | | |
|---|---|---|---|---|---|
| | | | | | |
| Probe set | pvalue | fc | Gene Symbol | Gene Title | Gene Ontology Biological Process |
| | **Samples 3-5** | | | | |
| 1438883_at | 0,004777313 | 4,61 | Fgf5 | fibroblast growth factor 5 | 8151 // cell growth and/or maintenance // inferred from electronic annotation /// 8283 // cell prolifera |
| 1419700_a_at | 0,000434875 | 3,33 | Prom1 | prominin 1 | 7602 // phototransduction // inferred from electronic annotation |
| 1416846_a_at | 0,005570535 | 3,21 | Pdzrn3 | PDZ domain containing RING finger 3 | 7242 // intracellular signaling cascade // inferred from sequence or structural similarity |
| 1418094_s_at | 0,000904162 | 3,14 | Car4 | carbonic anhydrase 4 | 6730 // one-carbon compound metabolism // inferred from electronic annotation |
| 1456329_at | 0,006459913 | 3,12 | Prtg | Protogenin | --- |
| 1456326_at | 0,00198988 | 3,12 | --- | Gene model 784, (NCBI) | --- |
| 1417216_at | 0,00314975 | 3,07 | Pim2 | proviral integration site 2 | 6916 // anti-apoptosis // inferred from direct assay /// 8637 // apoptotic mitochondrial changes // inf |
| 1416454_s_at | 0,005101781 | 3,04 | Acta2 | actin, alpha 2, smooth muscle, aorta /// RIKEN cDNA | 7010 // cytoskeleton organization and biogenesis // inferred from electronic annotation /// 7517 // m |
| 1448949_at | 0,006911907 | 2,95 | Car4 | carbonic anhydrase 4 | 6730 // one-carbon compound metabolism // inferred from electronic annotation |
| 1450047_at | 0,000956516 | 2,84 | Hs6st2 | heparan sulfate 6-O-sulfotransferase 2 | --- |
| 1426186_a_at | 0,001978461 | 2,83 | Fgf5 | fibroblast growth factor 5 | 8151 // cell growth and/or maintenance // inferred from electronic annotation /// 8283 // cell prolifera |
| 1452294_at | 0,000394643 | 2,77 | Pcdh1 | Protocadherin 1 | 7156 // homophilic cell adhesion // inferred from sequence or structural similarity /// 6355 // regulati |
| 1452249_at | 0,005064851 | 2,76 | Prickle1 | prickle like 1 (Drosophila) | --- |
| 1436512_at | 0,001222169 | 2,63 | Arl4c | ADP-ribosylation factor-like 4C | 6886 // intracellular protein transport // inferred from sequence or structural similarity /// 7264 // sma |
| 1454838_s_at | 0,004982912 | 2,58 | AW548124 | expressed sequence AW548124 | --- |
| 1421053_at | 0,002870865 | 2,53 | Kif1a | kinesin family member 1A | 7017 // microtubule-based process // inferred from electronic annotation |
| 1442434_at | 0,000100762 | 2,53 | D8Ertd82e * | DNA segment, Chr 8, ERATO Doi 82, expressed | --- |
| 1448182_a_at | 0,005939422 | 2,51 | Cd24a | CD24a antigen | --- |
| 1423104_at | 0,003558124 | 2,47 | Irs1 | insulin receptor substrate 1 | --- |
| 1447623_s_at | 0,000340965 | 2,39 | --- | --- | --- |
| 1460444_at | 0,002379187 | 2,36 | Arrb1 | arrestin, beta 1 | 8277 // regulation of G-protein coupled receptor protein signaling pathway // inferred from mutant p |
| 1433770_at | 0,007863733 | 2,17 | Dpysl2 | dihydropyrimidinase-like 2 | --- |
| 1431057_a_at | 0,002609858 | 2,13 | Prss23 | Protease, serine 23 | 6508 // proteolysis and peptidolysis // inferred from electronic annotation |
| 1452968_at | 0,004169029 | 2,13 | Cthrc1 | collagen triple helix repeat containing 1 | --- |
| 1434442_at | 0,004052645 | 2,12 | D5Ertd593e | DNA segment, Chr 5, ERATO Doi 593, expressed | --- |
| 1417895_a_at | 0,002598594 | 2,11 | Tmem54 | Transmembrane protein 54 | --- |
| 1454788_at | 0,004726676 | 2,11 | Arl7 | ADP-ribosylation factor-like 7 | 6886 // intracellular protein transport // inferred from sequence or structural similarity /// 7264 // sma |
| 1434822_at | 0,000720744 | 2,07 | Pphln1 | periphilin 1 | --- |
| 1420928_at | 0,003888065 | 2,06 | St6gal1 | beta galactoside alpha 2,6 sialyltransferase 1 | 6486 // protein amino acid glycosylation // inferred from electronic annotation |
| 1422962_a_at | 0,006706184 | 2,06 | Psmb8 | proteosome (prosome, macropain) subunit, beta type | 6955 // immune response // inferred from electronic annotation /// 6511 // ubiquitin-dependent prote |

| | | | | | |
|---|---|---|---|---|---|
| 1434909_at | 0,002403773 | 2,06 | Rragd | Ras-related GTP binding D | --- |
| 1417962_s_at | 0,004429903 | 2,03 | Ghr | growth hormone receptor | 6897 // endocytosis // inferred from electronic annotation |
| 1439485_at | 0,009695658 | 1,99 | --- | --- | --- |
| 1445503_at | 0,000634457 | 1,97 | --- | Gene model 715, (NCBI) | --- |
| 1460411_s_at | 0,005482096 | 1,97 | AW548124 | expressed sequence AW548124 | --- |
| 1429051_s_at | 0,000758536 | 1,96 | Sox11 | SRY-box containing gene 11 | --- |
| 1449022_at | 0,003243992 | 1,96 | Nes | nestin | 7399 // neurogenesis // inferred from direct assay |
| 1452127_a_at | 0,002918082 | 1,94 | Ptpn13 | protein tyrosine phosphatase, non-receptor type 13 | 7242 // intracellular signaling cascade // inferred from sequence or structural similarity /// 6470 // pr |
| 1434112_at | 0,001929385 | 1,93 | Lphn2 | latrophilin 2 | 7186 // G-protein coupled receptor protein signaling pathway // traceable author statement |
| 1422917_at | 0,001713479 | 1,91 | Epha1 | Eph receptor A1 | 6468 // protein amino acid phosphorylation // inferred from electronic annotation /// 7165 // signal tr |
| 1429315_at | 0,00231076 | 1,91 | Syt11 | synaptotagmin 11 | 6810 // transport // inferred from electronic annotation |
| 1425884_at | 0,002977836 | 1,91 | Bxdc1 | brix domain containing 1 | --- |
| | | | | | |
| * : | Genes with transient repression in Rev. Com. Cells | | | | |
| | (Genes in Cluster 2, Figure 3A) | | | | |

| Table 7 | | | | | |
|---|---|---|---|---|---|
| | | | | | |
| Probe set | pvalue | fc | Gene Symbol | Gene Title | Gene Ontology Biological Process |
| | Samples1-5 | | | | |
| 1438883_at | 0,001926601 | 11,34 | Fgf5 | fibroblast growth factor 5 | 8151 // cell growth and/or maintenance // inferred from electronic annotation /// 8283 // cell proliferation // in |
| 1448949_at | 4,37912E-05 | 6,05 | Car4 | carbonic anhydrase 4 | 6730 // one-carbon compound metabolism // inferred from electronic annotation |
| 1460038_at | 0,000995487 | 4,69 | Oct6/pou3f1*** | POU domain, class 3, transcription factor 1 | 6355 // regulation of transcription, DNA-dependent // inferred from electronic annotation |
| 1418094_s_at | 1,27225E-05 | 4,51 | Car4 | carbonic anhydrase 4 | 6730 // one-carbon compound metabolism // inferred from electronic annotation |
| 1417216_at | 0,000171305 | 4,47 | Pim2 | proviral integration site 2 | 6916 // anti-apoptosis // inferred from direct assay /// 8637 // apoptotic mitochondrial changes // inferred fro |
| 1425995_s_at | 0,000900575 | 4,32 | Wt1*** | Wilms tumor homolog | 30855 // epithelial cell differentiation // inferred from mutant phenotype /// 1654 // eye morphogenesis // infe |
| 1422068_at | 3,20269E-06 | 3,93 | Oct6/pou3f1*** | POU domain, class 3, transcription factor 1 | 6355 // regulation of transcription, DNA-dependent // inferred from electronic annotation |
| 1442655_at | 0,00187938 | 3,76 | Dnmt3b*** | DNA methyltransferase 3B | Imprinting |
| 1441429_at | 0,001057555 | 3,75 | Irs4*** | Insulin receptor substrate 4 | Insulin signaling |
| 1455029_at | 0,000343138 | 3,45 | Kif21a*** | Kinesin family member 21A | Kinesin complex, microtubule associated |
| 1452294_at | 0,000252338 | 3,44 | Pcdh1 | Protocadherin 1 | 7156 // homophilic cell adhesion // inferred from sequence or structural similarity /// 6355 // regulation of tra |
| 1416638_at | 0,000218472 | 3,44 | Sall2*** | sal-like 2 (Drosophila) | 6355 // regulation of transcription, DNA-dependent // inferred from electronic annotation |
| 1418351_a_at | 4,96538E-05 | 3,28 | Dnmt3b*** | DNA methyltransferase 3B | 6306 // DNA methylation // inferred from electronic annotation /// 6349 // imprinting // inferred from mutant p |
| 1449052_a_at | 0,000127272 | 3,09 | Dnmt3b*** | DNA methyltransferase 3B | 6306 // DNA methylation // inferred from electronic annotation /// 6349 // imprinting // inferred from mutant p |
| 1454681_at | 0,004696315 | 3,06 | Rbm35a | RNA binding motif protein 35A | RNA binding |
| 1418376_at | 0,000272991 | 3,06 | Fgf15 | fibroblast growth factor 15 | 7165 // signal transduction // inferred from electronic annotation |
| 1426186_a_at | 0,000815148 | 3,03 | Fgf5 | fibroblast growth factor 5 | 8151 // cell growth and/or maintenance // inferred from electronic annotation /// 8283 // cell proliferation // in |
| 1418517_at | 0,000177847 | 3,02 | Irx3 | Iroquois related homeobox 3 (Drosophila) | 6355 // regulation of transcription, DNA-dependent // inferred from electronic annotation |
| 1447825_x_at | 0,00049708 | 3,01 | Pcdh8 | protocadherin 8 | 16331 // morphogenesis of embryonic epithelium // inferred from mutant phenotype /// 1756 // somitogenes |
| 1419638_at | 0,000115998 | 3,01 | Efnb2*** | ephrin B2 | 7275 // development // inferred from electronic annotation /// 7399 // neurogenesis // inferred from electroni |
| 1425926_a_at | 7,82871E-05 | 2,96 | Otx2*** | orthodenticle homolog 2 (Drosophila) | 9952 // anterior/posterior pattern formation // inferred from mutant phenotype /// 45165 // cell fate commitme |
| 1452127_a_at | 0,001601088 | 2,94 | Ptpn13 | protein tyrosine phosphatase, non-receptor type 1 | 7242 // intracellular signaling cascade // inferred from sequence or structural similarity /// 6470 // protein am |
| 1450738_at | 0,000215887 | 2,94 | Kif21a | Kinesin family member 21A | Kinesin complex, microtubule associated |
| 1436600_at | 0,000748192 | 2,78 | Tnrc9 | trinucleotide repeat containing 9 | --- |
| 1440542_at | 0,00090133 | 2,78 | --- | RIKEN cDNA 7420416P09 | --- |
| 1435192_at | 0,001352773 | 2,70 | Sox3 | SRY-box containing gene 3 | establishment and maintenance of chromatin structure |
| 1419639_at | 0,000290409 | 2,68 | Efnb2*** | Ephrin B2 | Develpment/ Lymph vessel/ Integral to membrane |
| 1436398_at | 0,003952368 | 2,60 | Lef1*** | Lymphoid enhancer binding factor 1 | 16055 // Wnt receptor signaling pathway // inferred from electronic annotation /// 42475 // odontogenesis (s |
| 1436512_at | 0,001526224 | 2,56 | Arl4c | ADP-ribosylation factor-like 4C | GTPase activity/ rRNA processing |
| 1455114_at | 0,002037889 | 2,55 | Ung2 | Uracil DNA glycosylase 2 | Regulation of progression through cell cycle |

| | | | | | |
|---|---|---|---|---|---|
| 1418533_s_at | 4,6606E-06 | 2,54 | Fzd2 | frizzled homolog 2 (Drosophila) | 7186 // G-protein coupled receptor protein signaling pathway // inferred from electronic annotation /// 16055 |
| 1419700_a_at | 0,00203052 | 2,53 | Prom1 | prominin 1 | 7602 // phototransduction // inferred from electronic annotation |
| 1434909_at | 0,001751451 | 2,48 | Rragd | Ras-related GTP binding D | --- |
| 1421072_at | 0,000277559 | 2,48 | Irx5 | Iroquois related homeobox 5 (Drosophila) | 6355 // regulation of transcription, DNA-dependent // inferred from electronic annotation |
| 1415856_at | 0,000601252 | 2,47 | Emb | embigin | --- |
| 1448690_at | 0,003942414 | 2,46 | Kcnk1 | potassium channel, subfamily K, member 1 | 6811 // ion transport // inferred from electronic annotation /// 6813 // potassium ion transport // inferred from |
| 1454877_at | 0,00093237 | 2,45 | Sertad4 | SERTA domain containing 4 | --- |
| 1450047_at | 0,000423416 | 2,45 | Hs6st2 | heparan sulfate 6-O-sulfotransferase 2 | --- |
| 1421299_a_at | 0,002904802 | 2,42 | Lef1*** | lymphoid enhancer binding factor 1 | 16055 // Wnt receptor signaling pathway // inferred from electronic annotation /// 42475 // odontogenesis (s |
| 1439015_at | 0,000510657 | 2,41 | Gfra1 | glial cell line derived neurotrophic factor family rec | 9653 // morphogenesis // traceable author statement /// 7399 // neurogenesis // inferred from mutant pheno |
| 1418532_at | 4,17886E-05 | 2,40 | Fzd2 | frizzled homolog 2 (Drosophila) | 7186 // G-protein coupled receptor protein signaling pathway // inferred from electronic annotation /// 16055 |
| 1419204_at | 0,00156238 | 2,40 | Dll1*** | delta-like 1 (Drosophila) | 7154 // cell communication // inferred from electronic annotation /// 30154 // cell differentiation // inferred fro |
| 1452320_at | 7,86496E-05 | 2,40 | Lrp2*** | Low density lipoprotein receptor-related protein 2 | 6898 // receptor mediated endocytosis // inferred from mutant phenotype /// 6766 // vitamin metabolism // in |
| 1435437_at | 0,000101004 | 2,39 | Set7 | SET domain-containing protein 7 | 16568 // chromatin modification // inferred from electronic annotation |
| 1439665_at | 0,005832407 | 2,38 | Gpr23*** | G protein-coupled receptor 23 | --- |
| 1415857_at | 0,0004022 | 2,36 | Emb | embigin | --- |
| 1436959_x_at | 0,009948701 | 2,35 | Nelf | nasal embryonic LHRH factor | --- |
| 1452270_s_at | 0,000200308 | 2,34 | Cubn | Cubilin (intrinsic factor-cobalamin receptor) | 6898 // receptor mediated endocytosis // traceable author statement /// 6418 // tRNA aminoacylation for pro |
| 1448182_a_at | 0,000559301 | 2,33 | Cd24a | CD24a antigen | --- |
| 1457883_at | 0,004982177 | 2,32 | *** | Adult male aorta and vein cDNA, RIKEN full-lengt | --- |
| 1434362_at | 0,000562069 | 2,30 | --- | --- | --- |
| 1433983_at | 0,001672966 | 2,30 | Magi1 | membrane associated guanylate kinase interactin | --- |
| 1417574_at | 0,000748404 | 2,27 | Cxcl12 | chemokine (C-X-C motif) ligand 12 | 42098 // T-cell proliferation // inferred from mutant phenotype /// 7420 // brain development // inferred from |
| 1456632_at | 0,001544229 | 2,26 | Bcl11A*** | B cell lymphoma 11A | --- |
| 1427133_s_at | 0,000283254 | 2,26 | Lrp2*** | Low density lipoprotein receptor-related protein 2 | 6898 // receptor mediated endocytosis // inferred from mutant phenotype /// 6766 // vitamin metabolism // in |
| 1454788_at | 0,000640249 | 2,22 | Arl7 | ADP-ribosylation factor-like 7 | 6886 // intracellular protein transport // inferred from sequence or structural similarity /// 7264 // small GTPa |
| 1434822_at | 0,001698319 | 2,22 | Pphln1 | periphilin 1 | --- |
| 1416846_a_at | 0,003448645 | 2,20 | Pdzrn3 | PDZ domain containing RING finger 3 | 7242 // intracellular signaling cascade // inferred from sequence or structural similarity |
| 1456329_at | 0,000132902 | 2,19 | A230098A12Rik | RIKEN cDNA A230098A12 gene | --- |
| 1423064_at | 0,004126925 | 2,17 | Dnmt3a*** | DNA methyltransferase 3A | 6306 // DNA methylation // inferred from direct assay /// 6349 // imprinting // inferred from mutant phenotype |
| 1456326_at | 0,00580299 | 2,16 | Gm784 | Gene model 784, (NCBI) | Membrane |
| 1426990_at | 0,000121874 | 2,14 | Cubn | Cubilin (intrinsic factor-cobalamin receptor) | 6898 // receptor mediated endocytosis // traceable author statement /// 6418 // tRNA aminoacylation for pro |
| 1436030_at | 0,000359697 | 2,13 | Cachd1 | Cache domain containing 1 | Membrane |
| 1429690_at | 0,001592878 | 2,12 | 1300003B13Rik | RIKEN cDNA 1300003B13 gene | --- |

| | | | | | |
|---|---|---|---|---|---|
| 1433939_at | 0,004522205 | 2,12 | A730046J16 | hypothetical protein A730046J16 | --- |
| 1429372_at | 0,007349799 | 2,12 | Sox11 | SRY-box containing gene 11 | Transcription factor |
| 1423470_at | 0,001571354 | 2,12 | Ptbp2 | polypyrimidine tract binding protein 2 | 6376 // mRNA splice site selection // inferred from direct assay |
| 1433575_at | 0,002631655 | 2,11 | Sox4 | SRY-box containing gene 4 | 6355 // regulation of transcription, DNA-dependent // inferred from electronic annotation |
| 1448566_at | 0,002717423 | 2,11 | Slc40a1 | solute carrier family 40 (iron-regulated transporter | 6826 // iron ion transport // inferred from direct assay /// 6810 // transport // inferred from sequence or struct |
| 1440206_at | 0,00153026 | 2,11 | A930024E05Rik | RIKEN cDNA A930024E05 gene | --- |
| 1455374_at | 0,001166749 | 2,11 | --- | --- | --- |
| 1426614_at | 0,001177913 | 2,10 | Prkcbp1 | protein kinase C binding protein 1 | --- |
| 1451589_at | 0,006781723 | 2,08 | Gats | opposite strand transcription unit to Stag3 | --- |
| 1422631_at | 0,000762828 | 2,08 | Ahr | aryl-hydrocarbon receptor | 7049 // cell cycle // inferred from electronic annotation /// 6355 // regulation of transcription, DNA-dependen |
| 1423065_at | 0,000309895 | 2,08 | Dnmt3a*** | DNA methyltransferase 3A | 6306 // DNA methylation // inferred from direct assay /// 6349 // imprinting // inferred from mutant phenotype |
| 1420928_at | 0,004967884 | 2,08 | St6gal1 | beta galactoside alpha 2,6 sialyltransferase 1 | 6486 // protein amino acid glycosylation // inferred from electronic annotation |
| 1438200_at | 0,002274333 | 2,08 | Sulf1 | sulfatase 1 | 6915 // apoptosis // inferred from electronic annotation /// 8152 // metabolism // inferred from electronic ann |
| 1436031_at | 0,000738994 | 2,07 | Cachd1 | Cache domain containing 1 | Membrane |
| 1450070_s_at | 0,000996867 | 2,07 | Pak1 | P21 (CDKN1A)-activated kinase 1 | 16358 // dendrite morphogenesis // inferred from direct assay /// 6468 // protein amino acid phosphorylation |
| 1449147_at | 0,001736324 | 2,07 | Chst1 | carbohydrate (keratan sulfate Gal-6) sulfotransfer | --- |
| 1438454_at | 0,00491412 | 2,06 | B430203M17Rik*** | RIKEN cDNA B430203M17 gene | --- |
| 1416840_at | 0,002542451 | 2,06 | Mid1ip1 | Mid1 interacting protein 1 (gastrulation specific G1 | 7026 // microtubule stabilization // inferred from genetic interaction |
| 1417051_at | 0,001439205 | 2,05 | Pcdh8 | protocadherin 8 | 16331 // morphogenesis of embryonic epithelium // inferred from mutant phenotype /// 1756 // somitogenes |
| 1447623_s_at | 0,00441528 | 2,05 | --- | --- | --- |
| 1416034_at | 0,008998349 | 2,05 | Cd24a | CD24a antigen | --- |
| 1439485_at | 0,008190403 | 2,05 | Zfp608 | Zinc finger protein 608 | --- |
| 1448393_at | 0,004570949 | 2,04 | Cldn7 | claudin 7 | --- |
| 1417965_at | 0,000103836 | 2,03 | Plekha1 | Pleckstrin homology domain A1 | Lipid binding |
| 1454890_at | 0,003302907 | 2,03 | Amot | angiomotin | 6935 // chemotaxis // inferred from mutant phenotype /// 1570 // vasculogenesis // inferred from mutant phe |
| 1427912_at | 0,002270304 | 2,02 | Cbr3 | carbonyl reductase 3 | 8152 // metabolism // inferred from sequence or structural similarity |
| 1420650_at | 0,001734803 | 2,02 | Atbf1 | AT motif binding factor 1 | Transcription factor |
| 1457402_at | 0,004084036 | 2,02 | Sulf1 | Sulfatase 1 | 6915 // apoptosis // inferred from electronic annotation /// 8152 // metabolism // inferred from electronic ann |
| 1433745_at | 0,000444489 | 2,00 | Trio | triple functional domain (PTPRF interacting) | --- |
| 1421973_at | 0,001038046 | 2,00 | Gfra1 | glial cell line derived neurotrophic factor family rec | 9653 // morphogenesis // traceable author statement /// 7399 // neurogenesis // inferred from mutant pheno |
| 1443221_at | 0,002606729 | 2,00 | Wt1*** | Wilms tumor homolog | 30855 // epithelial cell differentiation // inferred from mutant phenotype /// 1654 // eye morphogenesis // infe |
| 1436319_at | 0,00366735 | 1,99 | Sulf1 | sulfatase 1 | 6915 // apoptosis // inferred from electronic annotation /// 8152 // metabolism // inferred from electronic ann |
| 1450779_at | 0,002761892 | 1,99 | Fabp7 | fatty acid binding protein 7, brain | 6810 // transport // inferred from electronic annotation |
| 1434699_at | 0,001092619 | 1,98 | 6030408C04Rik | RIKEN cDNA 6030408C04 gene | --- |

| | | | | | |
|---|---|---|---|---|---|
| 1454734_at | 0,0019642 | 1,98 | Lef1*** | lymphoid enhancer binding factor 1 | 16055 // Wnt receptor signaling pathway // inferred from electronic annotation /// 42475 // odontogenesis (s |
| 1437181_at | 4,0939E-05 | 1,97 | Peli2 | pellino 2 | 8063 // Toll signaling pathway // inferred from mutant phenotype |
| 1426926_at | 0,003934178 | 1,97 | Plcg2 | phospholipase C, gamma 2 | --- |
| 1434967_at | 0,001155773 | 1,96 | Zswim6 | zinc finger, SWIM domain containing 6 | --- |
| 1428647_at | 0,00064212 | 1,95 | 2310056B04Rik | RIKEN cDNA 2310056B04 gene | --- |
| 1421053_at | 0,001172874 | 1,95 | Kif1a | kinesin family member 1A | 7017 // microtubule-based process // inferred from electronic annotation |
| 1418534_at | 0,000611859 | 1,94 | --- | --- | --- |
| 1416407_at | 0,004228778 | 1,94 | Pea15 | phosphoprotein enriched in astrocytes 15 | 6915 // apoptosis // inferred from sequence or structural similarity /// 7242 // intracellular signaling cascade |
| 1419550_a_at | 0,003471077 | 1,94 | Stk39 | serine/threonine kinase 39, STE20/SPS1 homolog | 6468 // protein amino acid phosphorylation // inferred from electronic annotation |
| 1456377_x_at | 0,003643206 | 1,94 | Limd2 | LIM domain containing 2 | Metal ion binding |
| 1448747_at | 0,001526431 | 1,94 | Fbxo32 | F-box only protein 32 | 6810 // transport // inferred from electronic annotation /// 6512 // ubiquitin cycle // inferred from electronic an |
| 1419355_at | 0,004046662 | 1,94 | Klf7 | Kruppel-like factor 7 (ubiquitous) | 6355 // regulation of transcription, DNA-dependent // inferred from electronic annotation |
| 1428826_at | 0,000813379 | 1,93 | Nr6a1 | nuclear receptor subfamily 6, group A, member 1 | 122 // negative regulation of transcription from Pol II promoter // inferred from mutant phenotype /// 6355 // |
| 1417217_at | 0,000196783 | 1,93 | Magel2 | melanoma antigen, family L, 2 | 45449 // regulation of transcription // inferred from direct assay |
| 1434570_at | 0,000508411 | 1,93 | AK122525 | cDNA sequence AK122525 | --- |
| 1459722_at | 0,002840789 | 1,93 | Zswim6*** | zinc finger, SWIM domain containing 6 | Zinc ion binding |
| 1436925_at | 0,003986037 | 1,93 | Ches1 | checkpoint supressor 1 | 77 // DNA damage checkpoint // inferred from sequence or structural similarity /// 85 // G2 phase of mitotic c |
| 1460006_at | 0,009918604 | 1,92 | Atbf1*** | AT motif binding factor 1 | 6355 // regulation of transcription, DNA-dependent // inferred from electronic annotation |
| 1428738_a_at | 0,00050716 | 1,92 | D14Ertd449e | DNA segment, Chr 14, ERATO Doi 449, expresse | --- |
| 1435554_at | 0,003573525 | 1,92 | Tmcc3 | Transmembrane and coiled domains 3 | --- |
| 1450044_at | 0,009316424 | 1,91 | Fzd7*** | frizzled homolog 7 (Drosophila) | 7186 // G-protein coupled receptor protein signaling pathway // inferred from electronic annotation /// 16055 |
| 1448688_at | 0,000170745 | 1,91 | Podxl | podocalyxin-like | 8151 // cell growth and/or maintenance // inferred from electronic annotation |
| 1434709_at | 0,000880605 | 1,91 | C130076O07Rik | RIKEN cDNA C130076O07 gene | 7155 // cell adhesion // inferred from electronic annotation |
| 1438410_at | 0,001657393 | 1,90 | Prtg | Protogenin homolog | Striated muscle/ Thick filament membrane |
| 1452670_at | 0,000454139 | 1,90 | Myl9 | Myosin, light polypeptide 9, regulatory | 7517 // muscle development // inferred from sequence or structural similarity /// 6937 // regulation of muscle |
| 1417612_at | 0,005223441 | 1,90 | Ier5 | immediate early response 5 | --- |

# Additional file 1

**Tables 1 to 7 and legends**

For all tables, the Affymetrix probe set numbers, genes annotation (from NetAffx data base, last release from 21 July 2008) and fold change (fc) in expression are documented. Genes have been classified from the highest to the lowest fc. Cut off parameters have been chosen, for each table, as a way to include known regulated genes. Those ones served as positive controls for each analysis. More exhaustive analysis, with other parameter set up, could be done by using the complete data deposited at the GeneArray database under the accession number (pending). Sample numbers, 1 to 6, are referring to those indicated in Figure 1B, 1C, and 1D of the main text.

For all the Tables, multiple testing corrections have been performed using the Benjamini and Hochberg procedure and all genes in these tables have an FDR< 0,05.

**Table 1: Genes whose expression is induced (20 genes) or repressed (12 genes) by LIF at 24h upon LIF withdrawal:** Pair wise comparisons of samples 3 versus 4 after a student t-test and the following cut off parameters : pvalue < or equal to 0,05 and fc > or equal to 1,5 (for the induced genes) and < or equal to -1,5 (for the repressed genes).

**Table 2: Genes whose expression is induced (12 genes) or repressed (no genes) by LIF at 48h upon LIF withdrawal:** Pair wise comparisons of samples 5 versus 6 after a student t-test and the following cut off parameters : pvalue < or equal to 0,05 and fc > or equal to 1,5 (for the induced genes) and < or equal to -1,5 (for the repressed genes).

**Table 3: Genes whose expression is induced in pluripotent cells versus cells in which LIF has been withdrawn for 24h (59 genes):** Pair wise comparisons of samples 3 versus 1 after a student t-test and the following cut off parameters : pvalue < or equal to 0,017 and fc > or equal to 1,75. Known pluripotent markers are in bold and *Lifind* genes (see table 1) are in bold, italique.

**Table 4: Genes whose expression is repressed at 48h upon LIF withdrawal versus pluripotent cells (143 genes):** Pair wise comparisons of samples 5 versus 1 after a student t-test and the following cut off parameters : pvalue < or equal to 0,01 and fc < or equal to -1,9. Known pluripotent markers are in bold and *Lifind* genes (from Table 1) are in bold, italique.

**Table 5: Genes whose expression is repressed in pluripotent cells versus cells grown 24h without LIF (140 genes):** Pair wise comparisons of samples 3 versus 1 after a student t-test and the following cut off parameters : pvalue < or equal to 0,01 and fc < or equal to -1,9. Informations regarding profiling of these genes at 48h upon LIF withdrawal versus pluripotent cells are also documented. The genes quoted « NO » correspond to a new category of genes whose expression is transiently induced 24h after LIF withdrawal (reversible commitment state). Genes present in Cluster 7 (Figure 3A of the main text) are indicated by a double asterisk (**) and known differentiation markers are bolded.

**Table 6: Genes whose expression is induced at 48h upon LIF withdrawal versus 24h upon LIF withdrawal (41 genes):** Pair wise comparisons of samples 3 versus 5 after a student t-test and the following cut off parameters : pvalue < or equal to 0,01 and fc > or equal to 1,9.

**Table 7: Genes whose expression is induced at 48h upon LIF withdrawal versus pluripotent cells (114 genes):** Pair wise comparisons of samples 1 versus 5 after a student t-test and the following cut off parameters : pvalue < or equal to 0,01 and fc > or equal to 1,9. Genes induced at 24h of LIF withdrawal and whose expression was sustained at 48h are quoted by a triple asterisk (***). Known differentiation markers are bolded.
