# Supplementary material for: Three LIF-dependent signatures and gene clusters with atypical expression profiles, identified by transcriptome studies in mouse ES cells and early derivatives
Source: BMC Genomics. 2009 Feb 9;10:73. doi: 10.1186/1471-2164-10-73 (PMC2674464; doi:10.1186/1471-2164-10-73)
Supplement: Additional file 2 — Characterisation of Stat3Loc: probe set number: 1459961_a_at. Localisation of the sequence corresponding to this probe set, on the mouse genome. [file 1471-2164-10-73-S2.pdf]

Additional file 2

Characterisation of *Stat3Loc*: probe set n°: 1459961_a_at

<u>Sequence of the probe set :</u>
atgcacacatgctagacggatgaatggtgctggcggcacatgcccttaattccagcacttggaaggcagcagcagtggatctctaa
gaattaagggccagctggtctacaaattgagttccaggccagctaagaccctggcttaagatgaacaggaacaaaacactaagcagaa
gctggccatggatgtttcatgtctgcag

This sequence is complementary to the BAC where are located the *Stat3* and *Stat5a* loci as defined by BLAST search.

LOCUS AF246978 : 83848 bp

DEFINITION: Mus musculus transcription factor Stat5a (Stat5a) and transcription factor Stat3 (Stat3) genes, complete cds.

ACCESSION AF246978

VERSION AF246978.2 GI:18087725

78371 ctgcagacat gaaacatcca tggccagctt ctgcttagtg ttttgttcct
78421 gttcatctta agccagggtc ttagctggcc tggaactcaa tttgtagacc agctggccct
78481 taattcttag agatccactg ctgctgcctt ccaagtgctg gaattaaggg catgtgccgc
78541 cagcaccatt catccgtcta **gcatgtgtgc at (78572)**

The sequence corresponding to probe set 1459961_a_at (*Stat3Loc*) is located 5' to the ATG codon of *Stat3* on mouse chromosome 11 and therefore does not correspond to *Stat3* gene. Numbers in parenthesis correspond to nucleotide positions in the BAC sequence.
We renamed this gene *Stat3Loc*. to indicate the proximity of this unknown gene with the *Stat3* locus.

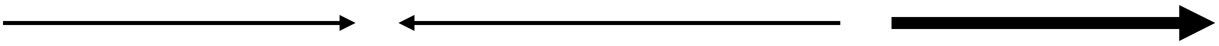

Gene *Stat5a* — **ATG** (3689) — Stop (26349)

Gene *Stat3* — Stop (30576) — **ATG** (53716)

1459961_a_at: Stat3Loc. — (78371) — (78572)
