# Supplementary material for: Three LIF-dependent signatures and gene clusters with atypical expression profiles, identified by transcriptome studies in mouse ES cells and early derivatives
Source: BMC Genomics. 2009 Feb 9;10:73. doi: 10.1186/1471-2164-10-73 (PMC2674464; doi:10.1186/1471-2164-10-73)
Supplement: Additional file 3 — Complete list of genes shown in the Figure 3A. [file 1471-2164-10-73-S3.pdf]

	**Additional file 3**		
	**Complete list of genes in Figure 3**		
	Lanes 15 to 74:	Cluster 1	"Com. and Dif." genes
	Lanes 75 to 86:	Cluster 2	Transient repression
	Lanes 87 to 130:	Cluster 3	"Dif." Markers
	Lanes 131 to 160:	Cluster 4	"Pluri" genes
	Lanes 161 to 222:	Cluster 5	"Pluri" genes
	Lanes 223 to 248:	Cluster 6	"Pluri and Com." genes
	Lanes 249 to 291:	Cluster 7	Transient induction
	Lanes 292 to 306:	Cluster 8	"Lifind" genes
	Pluri: Pluripotent	Com.: Commitment	Dif. Differentiation
**Lane 15**	1426043_a_at: Capn3; calpain 3		
	1436266_x_at: Cbx1 /// E430007M08Rik; chromobox homolog 1 Drosophila		
	1428647_at: 2310056B04Rik; RIKEN cDNA 2310056B04 gene		
	1435221_at: Foxp1; Forkhead box P1		
	1435222_at: Foxp1; Forkhead box P1		
	1444679_at: Bhc80; BRAF35/HDAC2 complex		
	1420965_a_at: Enc1; ectodermal-neural cortex 1		
	1427046_at: Tcfcp2l3; transcription factor CP2-like 3		
	1440542_at: ---; Similar to Protein C14orf115		
	1434853_x_at: Mkrn1; makorin, ring finger protein, 1		
	1426401_at: Ppp3ca; protein phosphatase 3, catalytic subunit, alpha isoform		
	1435434_at: Braf; Braf transforming gene		
	1449947_s_at: Atbf1; AT motif binding factor 1		
	1427302_at: Enpp3; ectonucleotide pyrophosphatase/phosphodiesterase 3		
	1452384_at: Enpp3; ectonucleotide pyrophosphatase/phosphodiesterase 3		
	1425926_a_at: Otx2; orthodenticle homolog 2 Drosophila		
	1420980_at: Pak1; P21 CDKN1A-activated kinase 1		
	1458218_s_at: Pde7a; phosphodiesterase 7A		
	1417051_at: Pcdh8; protocadherin 8		
	1447825_x_at: Pcdh8; protocadherin 8		
	1450738_at: ---; ---		
	1425995_s_at: Wt1; Wilms tumor homolog		
	1456632_at: ---; ---		
	1455029_at: ---; ---		
	1452214_at: 9130011J04Rik; RIKEN cDNA 9130011J04 gene		
	1424008_a_at: Rbpms2; RNA binding protein with multiple splicing 2		
	1419638_at: Efnb2; ephrin B2		
	1419639_at: ---; ---		
	1438973_x_at: Gja1; gap junction membrane channel protein alpha 1		
	1438650_x_at: Gja1; gap junction membrane channel protein alpha 1		
	1460038_at: ---; ---		
	1416638_at: Sall2; sal-like 2 Drosophila		
	1440206_at: A930024E05Rik; RIKEN cDNA A930024E05 gene		
	1420979_at: Pak1; P21 CDKN1A-activated kinase 1		

	1445710_x_at: 1110051B16Rik; RIKEN cDNA 1110051B16 gene
	1455114_at: C86987; expressed sequence C86987
	1434967_at: Zswim6; zinc finger, SWIM domain containing 6
	1422553_at: Pten; phosphatase and tensin homolog
	1454990_at: Arid2; AT rich interactive domain 2 Arid-rfx like
	1420649_at: Atbf1; AT motif binding factor 1
	1442655_at: ---; ---
	1455087_at: D7Ertd715e; DNA segment, Chr 7, ERATO Doi 715, expressed
	1436964_at: D7Ertd715e; DNA segment, Chr 7, ERATO Doi 715, expressed
	1437217_at: ---; ---
	1426722_at: Slc38a2; solute carrier family 38, member 2
	1454734_at: Lef1; lymphoid enhancer binding factor 1
	1418390_at: Bhc80; BRAF35/HDAC2 complex
	1418391_at: ---; ---
	1436398_at: Lef1; Lymphoid enhancer binding factor 1
	1450044_at: Fzd7; frizzled homolog 7 Drosophila
	1452309_at: 4933421H10Rik; RIKEN cDNA 4933421H10 gene
	1450655_at: Herc1; hect homologous to the E6-AP UBE3A carboxyl termin
	1454722_at: Herc1; hect homologous to the E6-AP UBE3A carboxyl termin
	1423064_at: Dnmt3a; DNA methyltransferase 3A
	1423065_at: Dnmt3a; DNA methyltransferase 3A
	1428843_at: Rnf153; ring finger protein 153
	1417392_a_at: Slc7a7; solute carrier family 7 cationic amino acid transporte
	1421299_a_at: Lef1; lymphoid enhancer binding factor 1
	1450396_at: Stag2; stromal antigen 2
**Lane 74**	1435603_at: Sned1; sushi, nidogen and EGF-like domains 1
**Lane 75**	1454729_at: B130017P16Rik; RIKEN cDNA B130017P16 gene
	1448871_at: Mapk13; mitogen activated protein kinase 13
	1442434_at: D8Ertd82e; DNA segment, Chr 8, ERATO Doi 82, expressed
	1437760_at: Galnt12; UDP-N-acetyl-alpha-D-galactosamine:polypeptide N-
	1449752_at: ---; ---
	1449198_a_at: Siat9; sialyltransferase 9 CMP-NeuAc:lactosylceramide alph
	1436865_at: Slc26a11; solute carrier family 26, member 11
	1456434_x_at: Hspb8; heat shock 27kDa protein 8
	1438118_x_at: Vim; vimentin
	1450350_a_at: Jundm2; Jun dimerization protein 2
	1450641_at: Vim; vimentin
**Lane 86**	1436141_at: ---; ---
**Lane 87**	1460556_at: D15Mit260; DNA Segment, Chr 15 Massachusetts Institute of
	1420611_at: Prkacb; protein kinase, cAMP dependent, catalytic, beta
	1448747_at: Fbxo32; F-box only protein 32
	1448949_at: Car4; carbonic anhydrase 4
	1418094_s_at: Car4; carbonic anhydrase 4
	1416407_at: Pea15; phosphoprotein enriched in astrocytes 15
	1417829_a_at: Rab15; RAB15, member RAS oncogene family
	1451589_at: Gats; opposite strand transcription unit to Stag3
	1434822_at: Pphln1; periphilin 1
	1426926_at: Plcg2; phospholipase C, gamma 2

	1438883_at: Fgf5; fibroblast growth factor 5
	1415856_at: Emb; embigin
	1415857_at: Emb; embigin
	1434362_at: ---; ---
	1452127_a_at: Ptpn13; protein tyrosine phosphatase, non-receptor type 13
	1417965_at: AA960558; expressed sequence AA960558
	1423272_at: Polg; polymerase DNA directed, gamma
	1425940_a_at: Ssbp3; single-stranded DNA binding protein 3
	1420842_at: Ptprf; protein tyrosine phosphatase, receptor type, F
	1426794_at: Ptprs; protein tyrosine phosphatase, receptor type, S
	1418517_at: Irx3; Iroquois related homeobox 3 Drosophila
	1427917_s_at: Ssbp3; single-stranded DNA binding protein 3
	1417379_at: Iqgap1; IQ motif containing GTPase activating protein 1
	1426186_a_at: Fgf5; fibroblast growth factor 5
	1452294_at: 2010005A06Rik; RIKEN cDNA 2010005A06 gene
	1437064_at: AW320017; expressed sequence AW320017
	1425266_a_at: Rap1gds1; RAP1, GTP-GDP dissociation stimulator 1
	1449368_at: Dcn; decorin
	1451912_a_at: Fgfrl1; fibroblast growth factor receptor-like 1
	1445503_at: ---; Gene model 715, NCBI
	1456603_at: 1500005K14Rik; RIKEN cDNA 1500005K14 gene
	1427894_at: Slitl2; Slit-like 2 Drosophila
	1417389_at: Gpc1; glypican 1
	1435256_at: AI844915; RIKEN cDNA 1500005P14 gene
	1418445_at: ---; ---
	1425784_a_at: Olfm1; olfactomedin 1
	1434442_at: D5Ertd593e; DNA segment, Chr 5, ERATO Doi 593, expressed
	1417189_at: Psme2; proteasome prosome, macropain 28 subunit, beta
	1417056_at: Psme1; proteasome prosome, macropain 28 subunit, alpha
	1431008_at: 0610037M15Rik; RIKEN cDNA 0610037M15 gene
	1422673_at: Prkcm; protein kinase C, mu
	1417978_at: Eif4e3; eukaryotic translation initiation factor 4E member 3
	1449461_at: Rbp7; retinol binding protein 7, cellular
**Lane 130**	1448436_a_at: Irf1; interferon regulatory factor 1
**Lane 131**	1448113_at: Stmn1; stathmin 1
	1431979_at: 4930444M15Rik; RIKEN cDNA 4930444M15 gene
	1455604_at: ---; ---
	1449090_a_at: Yes; Yamaguchi sarcoma viral v-yes oncogene homolog
	1417976_at: Ada; adenosine deaminase
	1454704_at: 9330185J12Rik; RIKEN cDNA 9330185J12 gene
	1439947_at: Cyp11a1; cytochrome P450, family 11, subfamily a, polypeptid
	1452076_at: 4633402N23Rik; RIKEN cDNA 4633402N23 gene
	1423281_at: Stmn2; stathmin-like 2
	1460235_at: Scarb2; scavenger receptor class B, member 2
	1430125_s_at: Pqlc1; PQ loop repeat containing 1
	1460227_at: Timp1; tissue inhibitor of metalloproteinase 1
	1437165_a_at: Pcolce; procollagen C-proteinase enhancer protein
	1416529_at: Emp1; epithelial membrane protein 1

	1448433_a_at: Pcolce; procollagen C-proteinase enhancer protein
	1448491_at: Ech1; enoyl coenzyme A hydratase 1, peroxisomal
	1422458_at: Tcl1; T-cell lymphoma breakpoint 1
	1434025_at: ---; ---
	1429399_at: Rnf125; ring finger protein 125
	1456242_at: ---; LOC433110
	1436905_x_at: Laptm5; lysosomal-associated protein transmembrane 5
	1433596_at: Dnajc6; DnaJ Hsp40 homolog, subfamily C, member 6
	1420410_at: Nr5a2; nuclear receptor subfamily 5, group A, member 2
	1425538_x_at: Ceacam1; CEA-related cell adhesion molecule 1
	1427630_x_at: Ceacam1; CEA-related cell adhesion molecule 1
	1460682_s_at: Ceacam2; CEA-related cell adhesion molecule 2
	1426858_at: Inhbb; Inhibin beta-B
	1449530_at: Trps1; trichorhinophalangeal syndrome I human
	1438214_at: Trps1; trichorhinophalangeal syndrome I human
**Lane 160**	1449706_s_at: ---; ---
**Lane 161**	1433657_at: A130092J06Rik; RIKEN cDNA A130092J06 gene
	1437100_x_at: Pim3; proviral integration site 3
	1425991_a_at: Ankrd25; ankyrin repeat domain 25
	1430781_at: Ak7; adenylate kinase 7
	1426025_s_at: Laptm5; lysosomal-associated protein transmembrane 5
	1453063_at: Cltb; clathrin, light polypeptide Lcb
	1454974_at: Ntn1; netrin 1
	1449064_at: Tdh; L-threonine dehydrogenase
	1460681_at: Ceacam2; CEA-related cell adhesion molecule 2
	1422937_at: Fzd5; frizzled homolog 5 Drosophila
	1422566_at: Tcfeb; transcription factor EB
	1435195_at: D930046M13Rik; RIKEN cDNA D930046M13 gene
	1420361_at: Slc11a1; solute carrier family 11 proton-coupled divalent metal
	1448610_a_at: Sod2; superoxide dismutase 2, mitochondrial
	1460429_at: Cdc5l; cell division cycle 5-like S. pombe
	1436926_at: Esrrb; estrogen related receptor, beta
	1418091_at: Tcfcp2l1; transcription factor CP2-like 1
	1434283_at: ---; ---
	1421840_at: Abca1; ATP-binding cassette, sub-family A ABC1, member 1
	1435040_at: Irak3; interleukin-1 receptor-associated kinase 3
	1426511_at: Susd2; sushi domain containing 2
	1449590_a_at: Mras; muscle and microspikes RAS
	1452532_x_at: Ceacam1; CEA-related cell adhesion molecule 1
	1422123_s_at: Ceacam2 /// Ceacam1; CEA-related cell adhesion molecule 2
	1450494_x_at: Ceacam1; CEA-related cell adhesion molecule 1
	1423686_a_at: 1110020C13Rik; RIKEN cDNA 1110020C13 gene
	1423280_at: Stmn2; stathmin-like 2
	1429377_at: 2410004A20Rik; RIKEN cDNA 2410004A20 gene
	1450860_at: Lap3; leucine aminopeptidase 3
	1437435_at: 1700061G19Rik; RIKEN cDNA 1700061G19 gene
	1423786_at: 8430410A17Rik; RIKEN cDNA 8430410A17 gene
	1422965_at: Agtrap; angiotensin II, type I receptor-associated protein

	1416715_at: Gjb3; gap junction membrane channel protein beta 3
	1418569_at: 2410043F08Rik; RIKEN cDNA 2410043F08 gene
	1449141_at: 2410043F08Rik; RIKEN cDNA 2410043F08 gene
	1451123_at: C330016O10Rik; RIKEN cDNA C330016O10 gene
	1449204_at: Gjb5; gap junction membrane channel protein beta 5
	1418133_at: Bcl3; B-cell leukemia/lymphoma 3
	1460700_at: Stat3; signal transducer and activator of transcription 3
	1417193_at: Sod2; superoxide dismutase 2, mitochondrial
	1453419_at: 2900078C09Rik; RIKEN cDNA 2900078C09 gene
	1429833_at: Ly6g6e; lymphocyte antigen 6 complex, locus G6E
	1418467_at: Smarcd3; SWI/SNF related, matrix associated, actin dependent
	1460596_at: Agtrap; angiotensin II, type I receptor-associated protein
	1418417_at: Msc; musculin
	1434500_at: ---; ---
	1417013_at: Hspb8; heat shock 27kDa protein 8
	1430208_at: 2410039E07Rik; RIKEN cDNA 2410039E07 gene
	1436164_at: Slc30a1; solute carrier family 30 zinc transporter, member 1
	1455229_x_at: 4933424M23Rik /// LOC260345; RIKEN cDNA 4933424M2
	1454045_a_at: 4933424M23Rik /// LOC260345; RIKEN cDNA 4933424M2
	1454046_x_at: 4933424M23Rik /// LOC260345; RIKEN cDNA 4933424M2
	1433645_at: 2210409B22Rik; RIKEN cDNA 2210409B22 gene
	1424719_a_at: Mapt; microtubule-associated protein tau
	1418470_at: Yes; Yamaguchi sarcoma viral v-yes oncogene homolog
	1460253_at: Cklfsf7; chemokine-like factor super family 7
	1426733_at: Itpk1; inositol 1,3,4-triphosphate 5/6 kinase
	1416066_at: Cd9; CD9 antigen
	1460454_at: 2010001H14Rik; RIKEN cDNA 2010001H14 gene
	1453442_at: 2310043M15Rik; RIKEN cDNA 2310043M15 gene
	1421839_at: Abca1; ATP-binding cassette, sub-family A ABC1, member 1
**Lane 222**	1425396_a_at: Lck; lymphocyte protein tyrosine kinase
**Lane 223**	1433436_s_at: Thtpa; thiamine triphosphatase
	1417460_at: Ifitm2; interferon induced transmembrane protein 2
	1436155_at: Nmnat2; nicotinamide nucleotide adenylyltransferase 2
	1454991_at: Slc7a1; Solute carrier family 7 cationic amino acid transporter,
	1452093_at: 2500001K11Rik; RIKEN cDNA 2500001K11 gene
	1417804_at: Rasgrp2; RAS, guanyl releasing protein 2
	1447678_at: ---; ---
	1426808_at: Lgals3; lectin, galactose binding, soluble 3
	1455333_at: BC023928; cDNA sequence BC023928
	1434917_at: Cobl; cordon-bleu
	1455300_at: E130014J05Rik; RIKEN cDNA E130014J05 gene
	1419417_at: Vegfc; vascular endothelial growth factor C
	1440739_at: Vegfc; vascular endothelial growth factor C
	1438781_at: ---; Adult male aorta and vein cDNA, RIKEN full-length enrich
	1454984_at: ---; ---
	1438872_at: BC050188; cDNA sequence BC050188
	1423465_at: Sdfr2; stromal cell derived factor receptor 2
	1417234_at: Mmp11; matrix metalloproteinase 11

	1439766_x_at: Vegfc; vascular endothelial growth factor C
	1415849_s_at: Stmn1; stathmin 1
	1453230_at: Zfp74; zinc finger protein 74
	1427140_at: Pvt1; plasmacytoma variant translocation 1
	1458295_at: BC038331; cDNA sequence BC038331
	1427087_at: Luc7l2; LUC7-like 2 S. cerevisiae
	1436994_a_at: Hist1h1c; histone 1, H1c
**Lane 248**	1456546_at: 1700097N02Rik; RIKEN cDNA 1700097N02 gene
**Lane 249**	1438802_at: ---; ---
	1419537_at: Tcfec; transcription factor EC
	1451687_a_at: ---; ---
	1435436_at: ---; ---
	1439123_at: Bhc80; BRAF35/HDAC2 complex
	1443526_at: Bhc80; BRAF35/HDAC2 complex
	1453976_at: 4432414F05Rik; RIKEN cDNA 4432414F05 gene
	1439582_at: ---; ---
	1423277_at: Ptprk; protein tyrosine phosphatase, receptor type, K
	1452940_x_at: Pitpnc1; phosphatidylinositol transfer protein, cytoplasmic 1
	1459973_x_at: ---; ---
	1456670_at: A930007A09Rik; RIKEN cDNA A930007A09 gene
	1417822_at: D17H6S56E-5; DNA segment, Chr 17, human D6S56E 5
	1454617_at: Arrdc3; arrestin domain containing 3
	1453013_at: 1110034O07Rik; RIKEN cDNA 1110034O07 gene
	1438084_at: ---; ---
	1444851_at: ---; ---
	1457314_at: ---; Similar to hypothetical protein FLJ10884
	1441243_at: ---; ---
	1430798_x_at: Mrpl15; mitochondrial ribosomal protein L15
	1437984_x_at: Bat1a; HLA-B-associated transcript 1A
	1457113_at: ---; ---
	1456705_at: C530030I18Rik; RIKEN cDNA C530030I18 gene
	1435669_at: Zfp532; zinc finger protein 532
	1438107_x_at: ---; ---
	1427193_at: Brd8; bromodomain containing 8
	1460600_at: ---; Hypothetical LOC245350
	1452657_at: Ap1s2; adaptor-related protein complex 1, sigma 2 subunit
	1430526_a_at: Smarca2; SWI/SNF related, matrix associated, actin depende
	1444426_at: F730031O20Rik; RIKEN cDNA F730031O20 gene
	1455544_at: Zranb3; zinc finger, RAN-binding domain containing 3
	1451559_a_at: Dhrs4; Dehydrogenase/reductase SDR family member 4
	1440522_at: B930075F07; hypothetical protein B930075F07
	1418388_s_at: 4930548G07Rik; RIKEN cDNA 4930548G07 gene
	1450853_at: Tle4; transducin-like enhancer of split 4, homolog of Drosophil
	1453208_at: 2700089E24Rik; RIKEN cDNA 2700089E24 gene
	1460588_at: ---; ---
	1455121_at: Mlr2; Mblk1-related protein-2
	1434004_at: Dhps; deoxyhypusine synthase
	1460490_at: Mrpl15; mitochondrial ribosomal protein L15

	1419241_a_at: Aire; autoimmune regulator autoimmune polyendocrinopathy				
	1452340_at: 6820424L24Rik; RIKEN cDNA 6820424L24 gene				
**Lane 291**	1443167_at: Rnf12; Ring finger protein 12				
**Lane 292**	1446583_at: ---; ---				
	1438331_at: ---; ---				
	1439349_at: BC019206; cDNA sequence BC019206				
	1458308_at: BC019206; cDNA sequence BC019206				
	1443721_x_at: BC019206; cDNA sequence BC019206				
	1457824_at: ---; ---				
	1447337_at: ---; ---				
	1423619_at: Rasd1; RAS, dexamethasone-induced 1				
	1416576_at: Socs3; suppressor of cytokine signaling 3				
	1456212_x_at: Socs3; suppressor of cytokine signaling 3				
	1455899_x_at: Socs3; suppressor of cytokine signaling 3				
	1415899_at: Junb; Jun-B oncogene				
	1459961_a_at: ---; ---				
	1452519_a_at: Zfp36; zinc finger protein 36				
**Lane 306**	1423100 at: Fos; FBJ osteosarcoma oncogene				
