# Supplementary material for: Three LIF-dependent signatures and gene clusters with atypical expression profiles, identified by transcriptome studies in mouse ES cells and early derivatives
Source: BMC Genomics. 2009 Feb 9;10:73. doi: 10.1186/1471-2164-10-73 (PMC2674464; doi:10.1186/1471-2164-10-73)
Supplement: Additional file 4 — Morphological changes of Alkaline phosphatase stained cells in various cell growth conditions (LIF withdrawal kinetic and knock-down of Oct4, Stat3 and Ier3 in the presence of LIF). [file 1471-2164-10-73-S4.pdf]

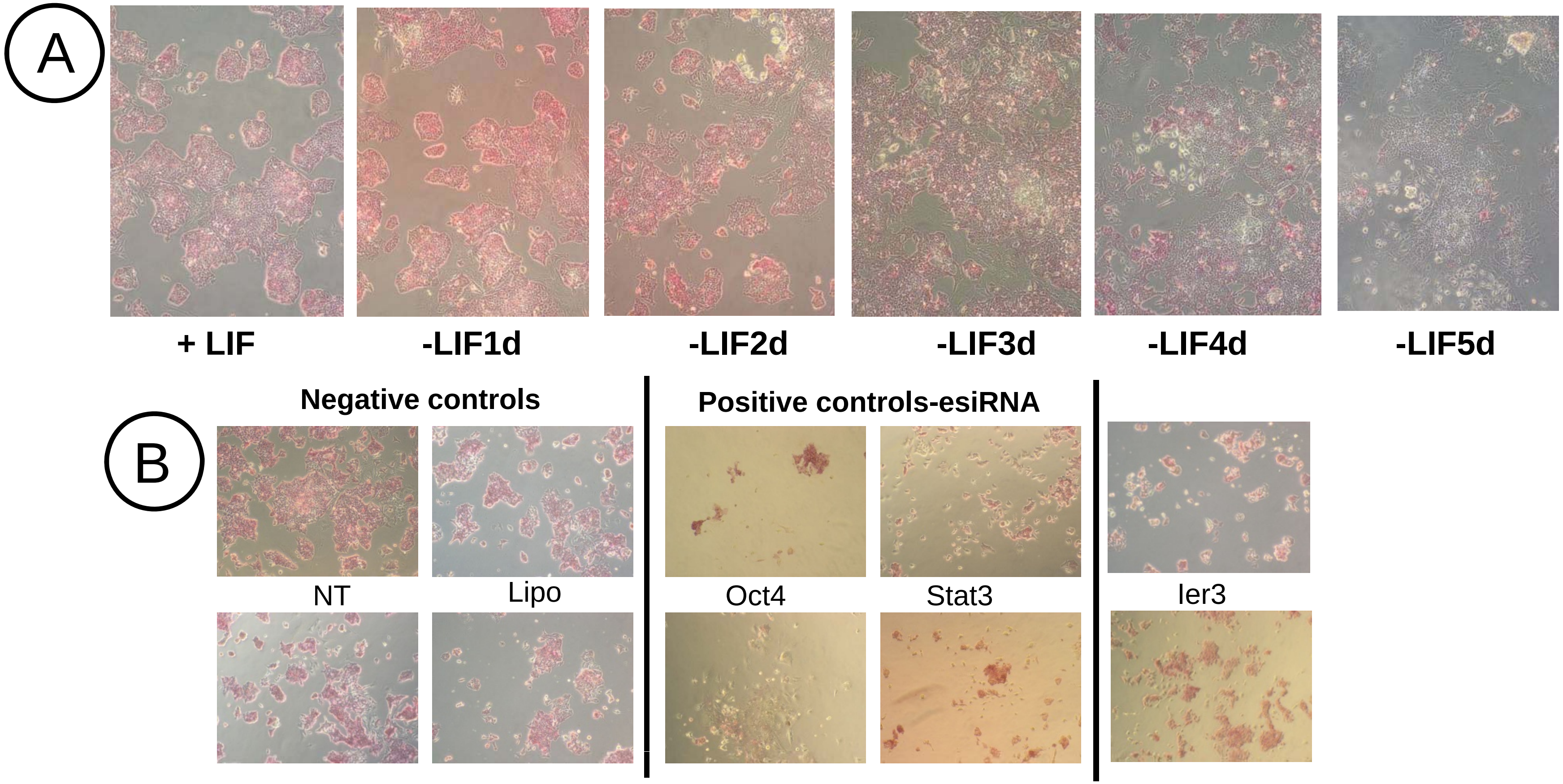

Additional file 4

Morphological changes of Alkaline phosphatase stained cells in various cell growth conditions

Pictures of Alkaline Phosphatase (ALP) staining of ES cells **A)** grown with LIF or depleted of LIF for the indicated times (d=day) or **B)** transfected twice with *Oct4*, *Stat3* or *Ier3* esiRNA. Fixation and ALP staining have been performed at day 5 after the first round of transfection. NT: Non Transfected cells, Lipo: Lipofectamine mocked transfected cells.

Significant fields of two independent experiments are shown. Magnication is X400 in **A)** and X100 in **B)**.
