# Supplementary material for: Three LIF-dependent signatures and gene clusters with atypical expression profiles, identified by transcriptome studies in mouse ES cells and early derivatives
Source: BMC Genomics. 2009 Feb 9;10:73. doi: 10.1186/1471-2164-10-73 (PMC2674464; doi:10.1186/1471-2164-10-73)
Supplement: Additional file 5 — Effects of the knock-down of a selection of Lifind and Pluri genes on the expression level of various committed and differentiation markers. [file 1471-2164-10-73-S5.pdf]

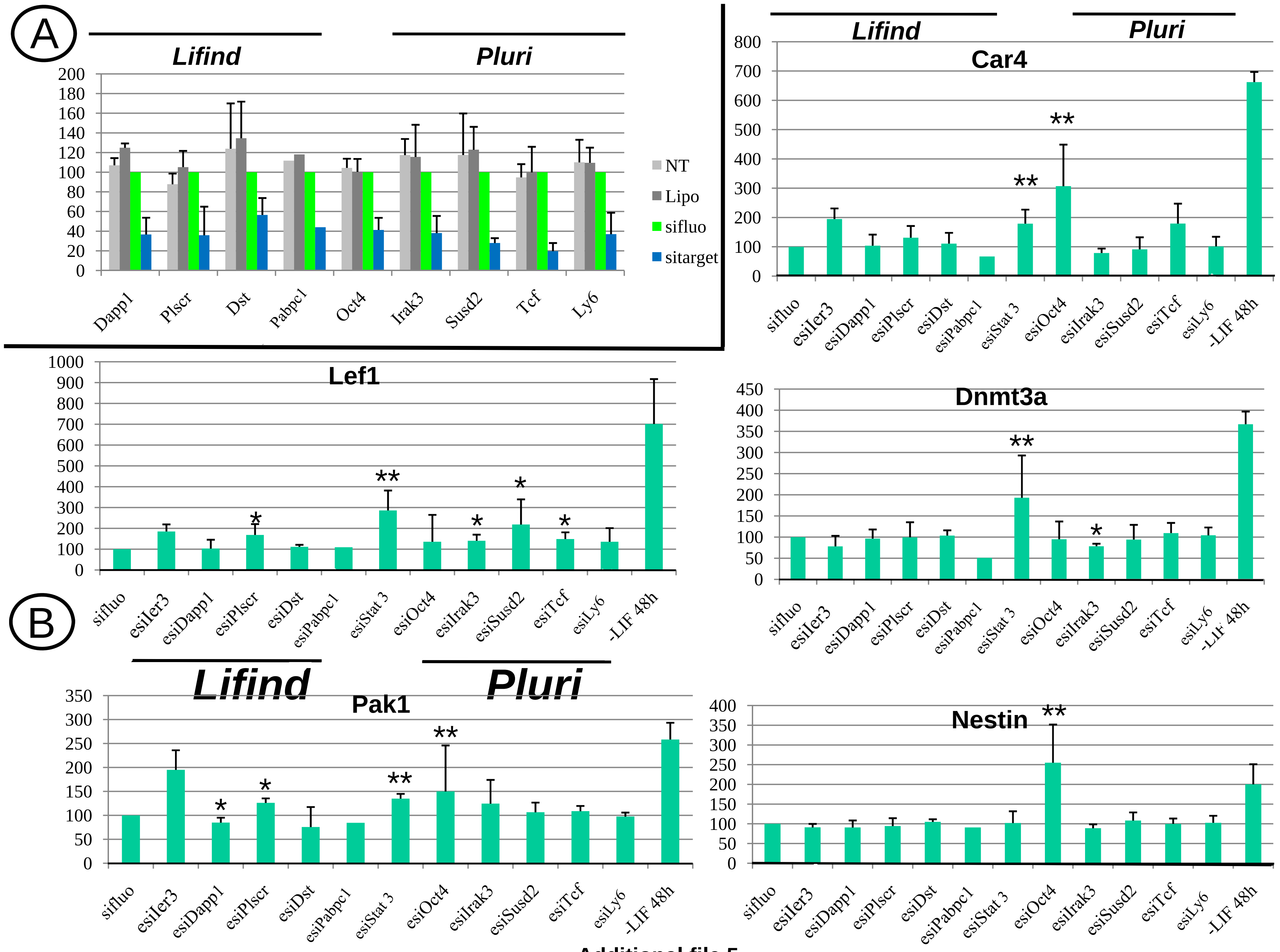

Additional file 5

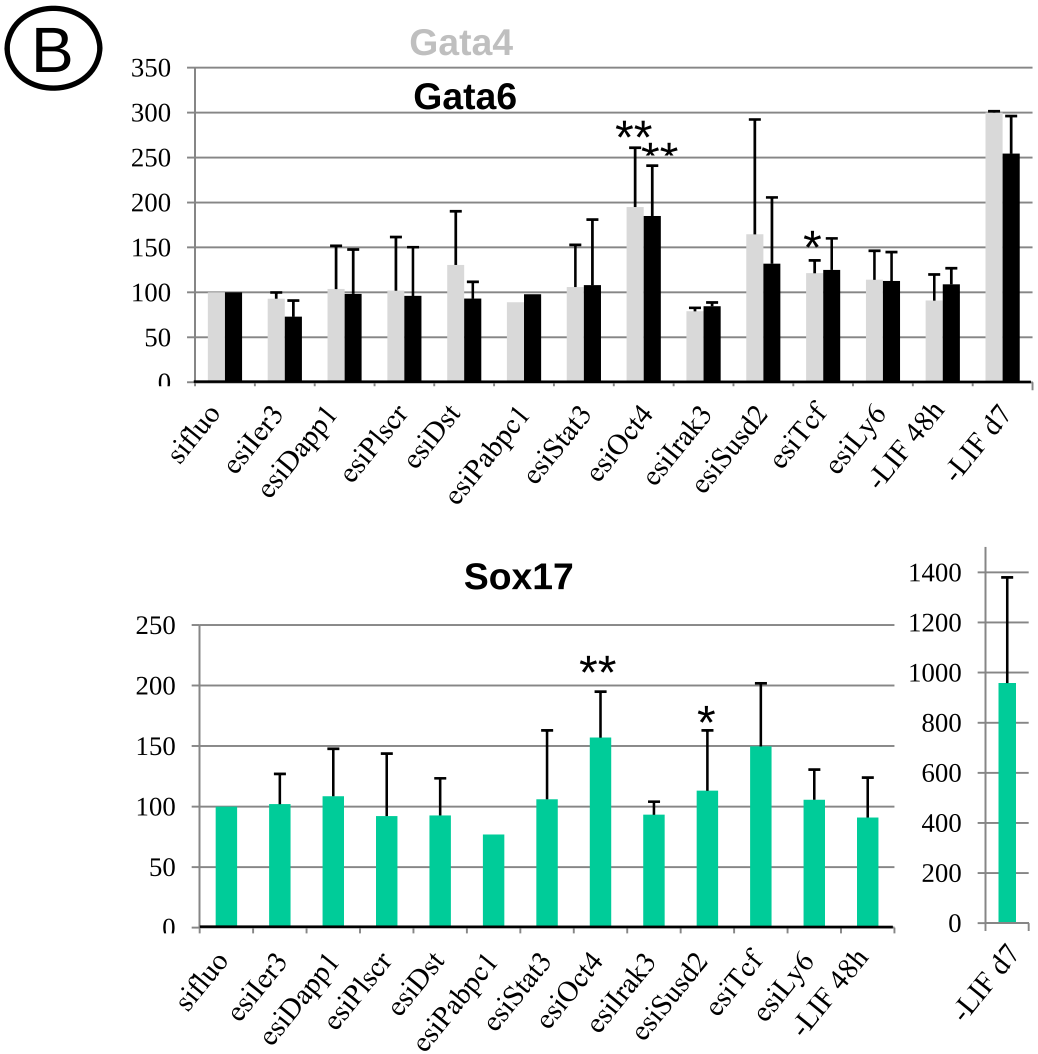

Additional file 5: Knock-down of a selection of *Lifind* and *Pluri* genes by the esiRNA strategy leads to minor changes in expression of early differentiation markers : A) Histogram representation of expression levels of a selection of *Lifind* and *Pluri* genes following RT-qPCR performed from ES cells transfected with the indicated esiRNAs (si target). NT and Lipo are as in Additional file 4. Sifluo: ES cells transfected with a fluorescent siRNA used as the base line control. **B)** Histogram representation of the expression levels of the indicated genes following transfection with the esiRNAs. The expression level of each marker, in cells grown without LIF for 48h (-LIF 48h) or for 7 days (-LIF d7), has also been included. The average of a minimum of three independent experiments has been plotted with standard deviations. Experiments with esi*Pabpc1* have been done twice. The estimated pvalue between samples was assessed by randomisation test according to Pfaffl. et al. (2002), Nucleic Acids Research, **30**, p36.

*: pvalue = 0.1; **: pvalue < or equal to 0,05.
