# Supplementary material for: Three LIF-dependent signatures and gene clusters with atypical expression profiles, identified by transcriptome studies in mouse ES cells and early derivatives
Source: BMC Genomics. 2009 Feb 9;10:73. doi: 10.1186/1471-2164-10-73 (PMC2674464; doi:10.1186/1471-2164-10-73)
Supplement: Additional file 6 — List of primers used for semi-quantitative RT-PCR analysis. [file 1471-2164-10-73-S6.pdf]

## Additional file 6

List of primers used for semi-quantitative RT-PCR analyses.

| Genes | Forward | reverse | PCR Products |
|---|---|---|---|
| Pak1 | TGCAACTGGACGAGTAATCC | GGGATGGCTGAGATTCTGTT | 136bp |
| Lef1 | AAGAAATGAGAGCGAATGTCGT | TTCTGGGACCTGTACCTGAAGT | 249bp |
| Fzd7 | TGTGTCTCTCTTTCGCATCC | GGAAGGCCTGCTCATAAAAG | 155bp |
| Dnmt3a | GCAGTCTCAACAGCACCATT | AGGTTTCCTGTGTGGTAGGC | 123bp |
| Mapk13 | TGACCCAGATCCTGAAAGTG | GTGGGAAGAGCTGTGTGAAA | 132bp |
| D8Ertd82e | GGGATGTCATTTCACACACG | AACTTGGTTCTGTCGGGATG | 132bp |
| Hspb8 | TCATTGGCTCACCTCAGTGT | CTTGGTGGTGATGGTTTGAG | 153bp |
| Car4 | GTAATGGCCCACTTCTGGAT | AGGCTGGGATTAAAGGTGTG | 145bp |
| embigin | CTCAGTGACGGAAAAGTCCA | GGGCTCATCATCTTTCTTCC | 142bp |
| Ptpn13 | CCTCACGTGCCATCAGTATT | CCATGAACCTCTGGCTGATA | 141bp |
| Irx3 | GACGACGAGGATGAGGAGAT | GTCGTCCGAGTCGCTAGTTT | 144 bp |
| 1434025_at | ACTGACTCGTCTGCCTTTGA | TTTGCTTGTGGTTTCTGTCC | 113bp |
| Ceacam1 | ACTTCAGCCCCTTCTTCTCC | AGACCCTCCAGTGAGGAACA | 137bp |
| Ceacam2 | GCACTCTCAGGAAACCTTGG | AGCACTGGTCCACTGGAAGT | 136bp |
| Inhbb | GAGACAGGTGGAATGTGGTG | CAGCTGTCACACTGCACATC | 139bp |
| Mras | ACAGGGCTACAGCTTCCAAA | GCGTTGTATGTGGGTAAGCA | 125bp |
| 1429377_at | TGTCCCAGAAGTGTCCTGAG | TATCCTGGCTCACACCAGAG | 140bp |
| 1423786_at | GCAGCAGTCAAAGGATGATG | TGGGCTCTTCTGGAGAAACT | 125bp |
| Gjb3 | ACTGCTGCTTGAGACACACC | CCGGGGATATGACAGGATAC | 140bp |
| Gjb5 | CCCTTCCCTGATTCTGACAT | ACACTCCAGTTCATGGTGGA | 137bp |
| Bcl3 | GAGAGCAGCAGTCGTCTCAG | GGCAGGTGTAGATGTTGTGG | 138bp |
| Vegfc | AAGACCGTGTGCGAATCGA | CACAGCGGCATACTTCTTC | 70bp |
| 1438781_at | CCGTACGTTATTGCTGGGTA | TGTCAGGAGAACCTTCGTGT | 158bp |
| 1454984_at | GGATCGGTTATGATGTGCAA | TTTTGAGTCGGAGTGAATCG | 124bp |
| Zfp74 | AGCACCAGAAAATCCAGGAC | GACTGTGCATGGAAGGAATG | 129bp |
| Smarca2 | TAAAGACCCCGTGAAGGAAG | CGCCCTGAACTGTCTTTGTA | 150bp |
| Tle4 | GGCTGTTTTGTGTCATGCTC | GGCTCTTCTTTGTGGCTTTC | 143bp |
| Aire | ACACCACCCTTCTCTTCCTG | GCCAGCTCTTGGTCCTAATC | 137bp |
| 1443167_at | AATGAGTGACCAGAGGCTTT | TCTTCATGGCCTAGCTTTCT | 138bp |
| Sbno2 | TATGAGATCAAGGCCACAGG | TGGAAGGCTGAGTCATGTTC | 130bp |
| Plscr1 | TGGGCTTTGGTGTGTTACAT | CAGCTCATTTGCATAGCTTC | 134bp |
| Dapp1 | AGAGCTTAGCCTTTCCCACA | GCTCTCACTTGGGTTCACCT | 130bp |

| | | | |
|---|---|---|---|
| Rasd1 | AATCGGATTCCTGGACTAGC | TTCCTTTCACAGCAGGTGAC | 122bp |
| Socs3 | CGTTGTGAAGAGGCAGTAGC | ACAAAGCAGAACCCTCCATC | 147bp |
| JunB | CCATCAACATGGAAGACCAG | CGTTCTCAGCCTTGAGTGTC | 144bp |
| 1459961_a_at | CATGCTAGACGGATGAATGG | TAGCTGGCCTGGAACTCAAT | 124bp |
| Zfp36 | CTTCACGACACACCAGATCC | TGGGAGTGCTGTAGTTGAGC | 136bp |
| Fos | GGCTCTCCTGTCAACACACA | CCGCTTGGAGTGTATCTGTC | 382bp |
| Nfkbiz | TGATAATGCCCGACAAGAAT | AATCCAATCCAATCCAAAGG | 160bp |
| Klf5 | ATTATTTGGGCCTGAGGTTG | TCTTCTGGCACCTGTCAACT | 123bp |
| Klf4 | TATACATTCCGCCACAGCAG | TCTGGGCTTCCTTTGCTAAC | 136bp |
| Egr1 | GAGCAAAAACGAGGAAGAGG | CCACAACACTCCAACTCCTG | 151bp |
| Egr2 | GTTCTGAACCTTCGGGAAAA | CACAAGGCACAGAGGACACT | 138bp |
| Egr3 | GATCCACCTCAAGCAAAAGG | AGGGAAAAGAAGGGATCTGG | 137bp |
| Ier2 | GTGAGCCTGAACTGAACCAA | GTCTACGGCAGCAACTACGA | 139bp |
| Ier3 | GGCAACGCTAACTCAGAACA | CATACACCCCTCCTTCACCT | 143bp |
| Hprt | TACAGGCCAGACTTTGTTGG | CAACTTGCGCTCATCTTAGG | 153bp |
| Ypel2 | GCTGTGCTGTGTGGTGTCTT | GCAATGCGATAGCCTTATCA | 153bp |
