# Supplementary material for: Three LIF-dependent signatures and gene clusters with atypical expression profiles, identified by transcriptome studies in mouse ES cells and early derivatives
Source: BMC Genomics. 2009 Feb 9;10:73. doi: 10.1186/1471-2164-10-73 (PMC2674464; doi:10.1186/1471-2164-10-73)
Supplement: Additional file 7 — List of primers used for RT-qPCR experiments. [file 1471-2164-10-73-S7.pdf]

Additional file 7

List of primers used for RT-qPCR experiments

nm_013556	HPRT F	tcagtcaacgggggacataaa
	HPRT R	ggggctgtactgcttaaccag
nm_010703	Lef1 F	aagaaatgagagagcgaatgtcgt
	Lef1 R	ttctgggacctgtacctgaagt
nm_011035	Pak1 F	tgcaactggacgagtaatcc
	Pak1 R	gggatggctgagattctgtt
nm_007607	Car4 F	gacaacggttcagagcacag
	Car4 R	aagcccttgttcaccttgtc
nm_007872	Dnmt3a F	gcagtctcaacagcaccatt
	Dnmt3a R	aggtttcctgtgtggtaggc
nm_016701	Nestin F	agaatgtgcagtcaccaagg
	Nestin R	gggtctcattttcaggtgggt
nm_008092	Gata4 F	ccctacccagcctacatgg
	Gata4 R	acatatcgagattggggtgtct
nm_010258	Gata6 F	ttgctccggtaacagcagtg
	Gata6 R	gtggtcgcttgtgtagaagga
nm_011441	Sox17 F	gatgcgggatacgccagtg
	Sox17 R	ccaccacctcgcctttcac
nm_0133662	Ier3 F	gctctggtcccgagattttca
	Ier3 R	agatgatggcgaacaggagaa
nm_011932	Dapp1 F	gcagaacttctaggagggaaca
	Dapp1 R	ccgtgataccaccccaaatc
nm_011636	Plscr1 F	ttctggaagtcttagcaggct
	Plscr1 R	cagtttcgggtacagcagtca
nnm_133833	Dst F	agaacggaacctatttgcatgaa
	Dst R	ctggtgctgagccatgatctt
nm_008774	Pabpc1 F	caagccagtacgcatcatgtg
	Pabpc1 R	tgcttcctgtgtttcaaagtgt
nm_013633	Oct4 F	gaggagtcccaggacatgaa
	Oct4 R	agatggtggtctggctgaac
nm_213659	Stat3 F	caacatcctggtgtctccac
	Stat3 R	caatggtattgctgcaggtc
nm_028679	Irak3 F	acatggggcatcaacgagc
	Irak3 R	ggaagctgataggggttttctg
nm_027890	Susd2 F	tgatgggtggcaaagacttcg
	Susd2 R	agcaagggtgatatgcagtgc
nm_023755	Tcfcp2l1 F	gaatcggaagctaggggactt
	Tcfcp2l1 R	ccctggggtccaagataccaa
nm_027366	Ly6g6e F	tactggtcacggtcctactct
	Ly6g6e R	ggcagcattgcataggtcct
